# Degranulation enhances presynaptic membrane packing, which protects NK cells from perforin-mediated autolysis

**DOI:** 10.1371/journal.pbio.3001328

**Published:** 2021-08-03

**Authors:** Yu Li, Jordan S. Orange

**Affiliations:** 1 Department of Pathology and Immunology, Baylor College of Medicine, Houston, Texas, United States of America; 2 Department of Pediatrics, Vagelos College of Physicians and Surgeons, Columbia University Irving Medical Center, New York, New York, United States of America; University of Bern, SWITZERLAND

## Abstract

Natural killer (NK) cells kill a target cell by secreting perforin into the lytic immunological synapse, a specialized interface formed between the NK cell and its target. Perforin creates pores in target cell membranes allowing delivery of proapoptotic enzymes. Despite the fact that secreted perforin is in close range to both the NK and target cell membranes, the NK cell typically survives while the target cell does not. How NK cells preferentially avoid death during the secretion of perforin via the degranulation of their perforin-containing organelles (lytic granules) is perplexing. Here, we demonstrate that NK cells are protected from perforin-mediated autolysis by densely packed and highly ordered presynaptic lipid membranes, which increase packing upon synapse formation. When treated with 7-ketocholesterol, lipid packing is reduced in NK cells making them susceptible to perforin-mediated lysis after degranulation. Using high-resolution imaging and lipidomics, we identified lytic granules themselves as having endogenously densely packed lipid membranes. During degranulation, lytic granule–cell membrane fusion thereby further augments presynaptic membrane packing, enhancing membrane protection at the specific sites where NK cells would face maximum concentrations of secreted perforin. Additionally, we found that an aggressive breast cancer cell line is perforin resistant and evades NK cell–mediated killing owing to a densely packed postsynaptic membrane. By disrupting membrane packing, these cells were switched to an NK-susceptible state, which could suggest strategies for improving cytotoxic cell-based cancer therapies. Thus, lipid membranes serve an unexpected role in NK cell functionality protecting them from autolysis, while degranulation allows for the inherent lytic granule membrane properties to create local ordered lipid “shields” against self-destruction.

## Abstract 2

Natural killer cells mediate largely unidirectional potent cytotoxicity against diseased cells while sparing themselves. The authors show that the NK cell membrane contains and focuses lipids of high density which shield against self-destruction, and a similar densely packed postsynaptic membrane is responsible for the perforin resistance and NK cell-mediated killing evasion of an aggressive breast cancer cell line.

## Introduction

Immune defense against intracellular pathogens and tumors by cytotoxic effector cells, which include natural killer (NK) cells and cytotoxic T lymphocytes (CTLs), is fundamental to human health. NK cells in particular have an inherent ability to recognize and destroy infected and transformed cells. They express high levels of cytolytic effector molecules without the need for clonal expansion and use these to kill diseased cells. Upon recognition of a target, an NK cell establishes an immunological synapse to initiate a series of tightly regulated downstream events [1]. These include the mobilization of lysosome related organelles, known as lytic granules that are specialized for secretion. During the progression of the synapse, these lytic granules initially converge to the microtubule organizing center (MTOC) and then polarize along with the MTOC to the synapse formed with the target cell [2]. Once delivered to the synapse, the lytic granules navigate a meshwork of filamentous actin and find openings through which they may access the NK cell plasma membrane [3]. This allows the lytic granules to dock and fuse with the plasma membrane and then utilize myosin-II to extrude their cytotoxic contents into a protected synaptic cleft between the NK cell and target cell [4,5]. Therein, the lytic effector molecules can affect the destruction of the target cell.

Among the destructive molecules contained within the lytic granules, perforin serves a critical nonredundant function and is an absolute requirement for NK cell cytotoxicity [6]. Perforin is a pore-forming protein that penetrates the target cell membrane and allows for the uptake of proapoptotic serine proteases, such as granzymes A and B that are also extruded into the synaptic cleft from the lytic granule. Although perforin is a potent pore-forming effector, its activity is strictly inhibited inside the lytic granule owing to the fact that the internal environment of the granule is both acidic and hypocalcemic [7]. Once secreted into the nonacidic synaptic cleft, perforin attains its functionality in membrane attack via its calcium-activated C2 domain, which mediates the hydrophobic interaction between the perforin molecule and a target membrane [8].

Due to the nonspecific nature of the hydrophobic interaction between perforin and a target cell membrane, the NK cell membrane theoretically shares the same vulnerability as that of target cell. That said, NK cells are generally not susceptible to the released cytotoxic contents of their lytic granules. Even though both the NK and the target cell membranes are exposed to perforin within the confined space of synaptic cleft, less than 5% of NK cells undergo autolysis during target engagement [9]. This unidirectional killing is presumably necessary for the immune system to preserve NK cells within a diseased environment for additional killing and host defense. NK cells are quite effective in serial killing and on average can mediate approximately 6 separate kills within a 24-h period [10]. It is presently unclear, however, as to why NK cells maintain a survival advantage upon their degranulation relative to that of the target cells. That said, perforin-mediated pore formation does occur predominantly in the target cell membrane [11]. Thus, there must be a membrane-protective mechanism of some kind to ensure the resistance of NK cells to their own perforin upon its release into the synaptic cleft.

Protection from perforin permeabilization is not appreciated as an intrinsic property of NK cell membranes. Early studies have demonstrated fratricide between NK cells, implying that protection from perforin is not a default but is potentially acquired during the target killing process [12]. Several hypotheses have been proposed to explain this relative and potentially augmented resistance of NK cells during target engagement. These include lytic granule–derived cell surface cathepsin B providing self-protection for cytotoxic lymphocytes [13]. However, the universal protective function of cathepsin B is questionable since CTLs from cathepsin B knockout mice have normal survival post-degranulation, indicating that this protease is not exclusive for perforin inactivation [14]. Another hypothesis has involved a protective role for LAMP-1, frequently used as a marker for degranulation, which has been shown to facilitate the delivery of perforin to the target cell membrane [15]. It was proposed that the steric hindrance and negative charges of heavily glycosylated LAMP-1 could prevent the binding of perforin to the membrane [16]. Recent work [17], however, has demonstrated that under some circumstances, LAMP-1 is dispensable for protection against perforin-mediated autolysis and has shown the relevance of the cytotoxic T cell membranes themselves. Thus, a protective mechanism relying on membrane-bound proteins might be contributory to resistance in some cases but possibly locally overcome by secreted perforin under other circumstances.

Despite there being a number of potential mechanisms contributing to NK cell resistance to perforin, the question as to how they are protected from autolysis upon degranulation remains unanswered. In light of the charge-sensitive nature of hydrophobic interactions, we considered particular properties of the presynaptic membrane that might make NK cells more resistant to perforin upon degranulation. We demonstrate that perforin binding to NK cell membranes is inhibited by them having high lipid ordering caused by the coalescence of high order lipid membrane domains (or rafts—a known property of the presynaptic membrane) [18–20]. Furthermore, we identify a specific feature of lytic granules in that they have endogenously densely packed membranes. This further enhances lipid packing and potential protection against perforin at the specific sites of degranulation where NK cells face the highest perforin concentrations. Additionally, we find that an aggressive breast cancer cell line utilizes the same strategy to evade NK cell–mediated killing by assembling a densely packed postsynaptic membrane to block perforin attack. Disrupting these protective membrane packing mechanisms reverses NK cell protection against autolysis and, in the case of the breast cancer cell line, effectively switches it to an NK-susceptible state.

## Results

### NK cells assemble presynaptic membranes with high lipid order during target cell engagement

NK cells form immunological synapses with target cells to initiate a series of tightly regulated downstream events that eventually release perforin and other cytolytic molecules into synaptic clefts [21]. Despite the close range between NK and target cell membranes defining the synaptic cleft, perforin-mediated pore formation predominantly occurs in the target cell membrane [11]. Ultimately, this results in the death of the target and survival of the NK cell upon its degranulation. In considering this unidirectional attack of perforin, it is interesting that the membrane targeting of perforin to the target cell does not rely on any particular receiving ligand and is solely mediated by hydrophobic interaction [22]. Since these hydrophobic interactions are charge sensitive, we postulated that coalescence of high order lipid membrane domains, which can greatly affect the charge distribution on presynaptic membrane [23], might prevent perforin binding. If true, NK cells could form high lipid order membranes to inhibit perforin activity and allow for their unidirectional attack against target cells.

**Fig 1:**
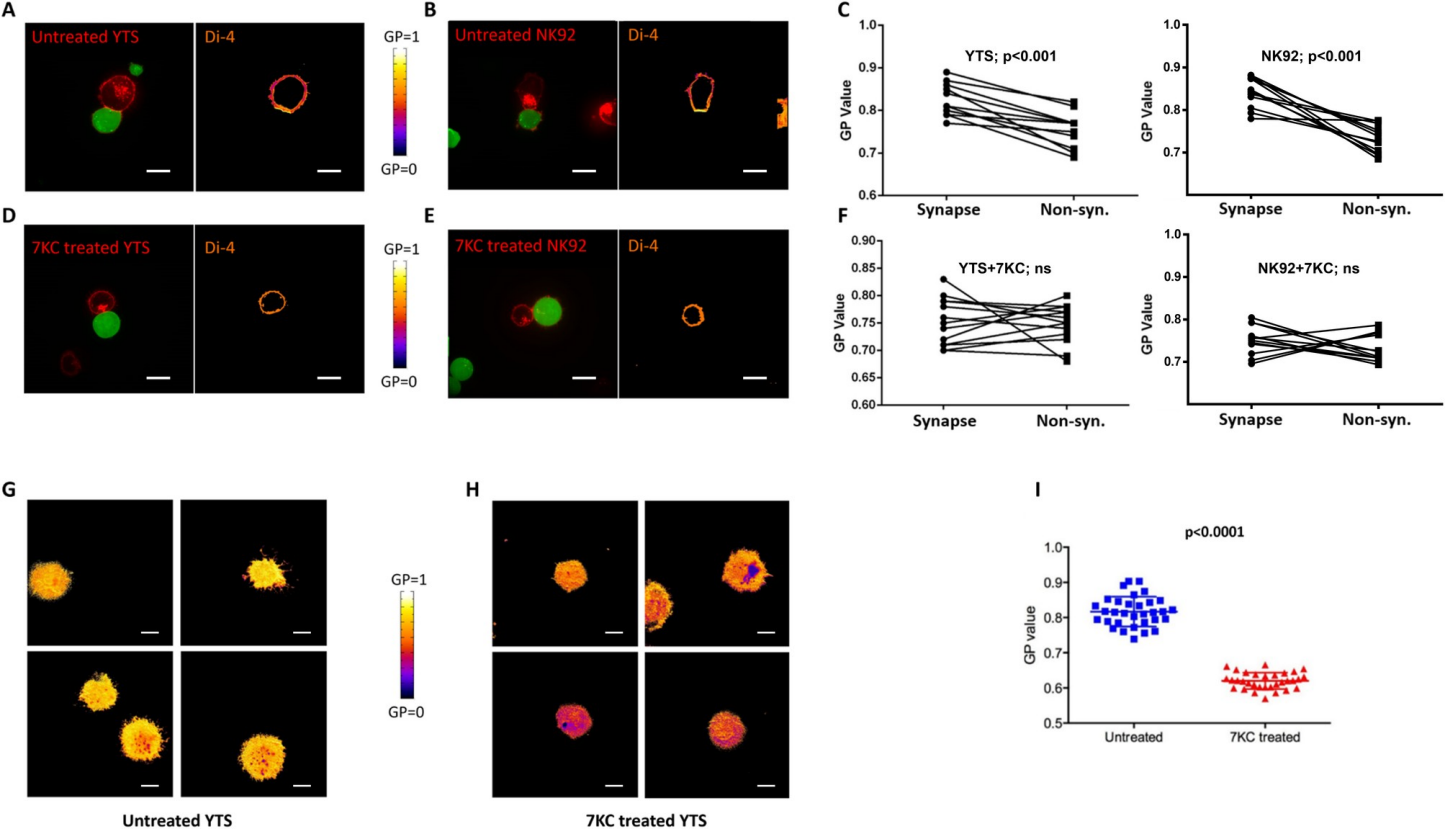
NK cells assemble presynaptic membranes with high lipid order during target cell engagement. The lipid packing of NK cell membranes in fluorescent packing sensor labeled YTS (A, D) and NK92 (B, E) conjugated with calcein green–loaded 721 or K562 target cells, respectively, was measured using live cell confocal microscopy (left image). 7KC-treated and untreated NK cells were overlaid with a pseudocolor scale (A, B, D, E–right image) to demonstrate differences in packing. Membrane lipid density was quantified as a GP value, which was measured in distinct conjugates (*n =* 12 for untreated YTS, *n* = 14 for 7KC-treated YTS, *n* = 12 for untreated NK92, *n* = 12 for 7KC-treated NK92) performed over 4 independent experiments and the images shown were representative. The individual GP values for synaptic and nonsynaptic regions were compared as paired observations from individual cells in aggregate for YTS (C left, *p* < 0.001) and NK92 (C right, *p* < 0.001) cells using a paired *t* test and the difference was significant, but not when YTS (F left, *p* > 0.05, ns) and NK92 (F right, *p* > 0.05, ns) cells were treated with 7KC. The lipid packing of the NK cell presynaptic membrane was further measured using TIRF microscopy on activating glass surfaces (anti-CD18 with anti-CD28 for YTS), and 4 representative images from untreated (G) and 7KC-treated YTS cells (H) were overlaid with a pseudocolor scale to allow visualization of packing differences (G vs H). Packing density was quantified as a GP value in 32 distinct cells from 4 independent experiments, and an unpaired *t* test was used for the comparison of the means (I) and the difference was significant *p* < 0.0001. Scale bar: 10 μm. GP, generalized polarization; NK, natural killer; ns, not significant; TIRF, total internal reflection fluorescence; 7KC, 7-ketocholesterol.

To test our hypothesis, we first attempted to measure the lipid ordering of the NK cell membrane in living cells using 2 fluorescent sensors: Di-4-ANEPPDHQ and Laurdan, whose emission spectra are dependent upon lipid packing (S1A and S1B Fig). Because these 2 sensors take advantage of different mechanisms to probe lipid packing density [24] and were applied across multiple NK–target cell pairs, our measurements should be technically reduced in bias and also not dependent upon any particular cell line. The lipid packing of NK cell membranes was quantified using previously established ratiometric analysis [25] and represented as generalized polarization (GP) values (S1C and S1D Fig). The lipid packing density of the entire NK cell plasma membrane was initially measured and specifically evaluated relative to the site of contact with target cells. NK cells (YTS and NK92) were prelabeled with sensors and then conjugated to target cells (721.221 and K562, respectively) and imaged using live cell confocal microscopy. In both YTS and NK92 cells, more densely packed membranes were observed at the immunological synapse, while the nonsynaptic regions displayed relatively lower packing (consistent with previous findings using indirect methods or in fixed cells) [20,26]. The increased membrane lipid packing at the synapse was significant for both YTS and NK92 cells across repeated experiments (Fig 1A–1C). Nonsynaptic regions had a GP value that was similar to that of the plasma membranes in unconjugated NK cells (S2A and S2B Fig), indicating that the uneven distributions of membrane packing in conjugated NK cells was likely due to an increase of lipid ordering at the synapse. This is also consistent with previously published results from CTLs and antigen-presenting cells (APCs) showing lipid raft aggregation at the immunological synapse [27]. Thus, during target cell engagement, NK cells form a presynaptic membrane characterized by having high lipid order.

To evaluate the potential role of increased lipid ordering in the NK cell presynaptic membrane, we utilized 7-ketocholesterol (7KC). 7KC is effective in disrupting lipid packing and functions by steric hindrance resulting from an additional ketone group it possesses. It has been shown to be effective in cytotoxic lymphocytes as it can disrupt lipid ordering in the plasma membrane of CTL cells without interfering other biological properties such as viability and proximal signaling [28]. In order to modulate membrane packing density in NK cells, we pretreated NK cells with 7KC, labeled them with lipid packing sensors, and then conjugated them to target cells. We then measured their membrane packing distribution during target engagement using live cell confocal microscopy. Using this approach, 7KC treatment reduced the presence of lipid ordering at the immunological synapse (Fig 1D–1F). Furthermore, the membranes of NK cells in conjugates displayed evenly distributed lipid packing after 7KC treatment.

To further study the lipid packing on the presynaptic membrane with higher spatial resolution, en face images of the immunological synapse were acquired using total internal reflection fluorescent (TIRF) microscopy. TIRF provides increased contrast by confining sample illumination to a depth of approximately 150 nm from the plane of the coverslip. NK cells were lipid sensor prelabeled and seeded on glass surfaces coated with antibodies against activating receptors to generate immunological synapses at the plane of the glass and visualized using TIRF imaging. NK cells had densely packed membranes at the glass surface, corresponding to the presynaptic membrane, indicated by the high GP values (Fig 1G and 1I). Importantly, this high degree of ordering was completely eliminated after 7KC pretreatment of the NK cells (Fig 1H and 1I). Thus, during target cell engagement, NK cells form a densely packed presynaptic membrane, which can be effectively disrupted by 7KC.

### 7-Ketocholesterol induces NK cell death during target killing

**Fig 2:**
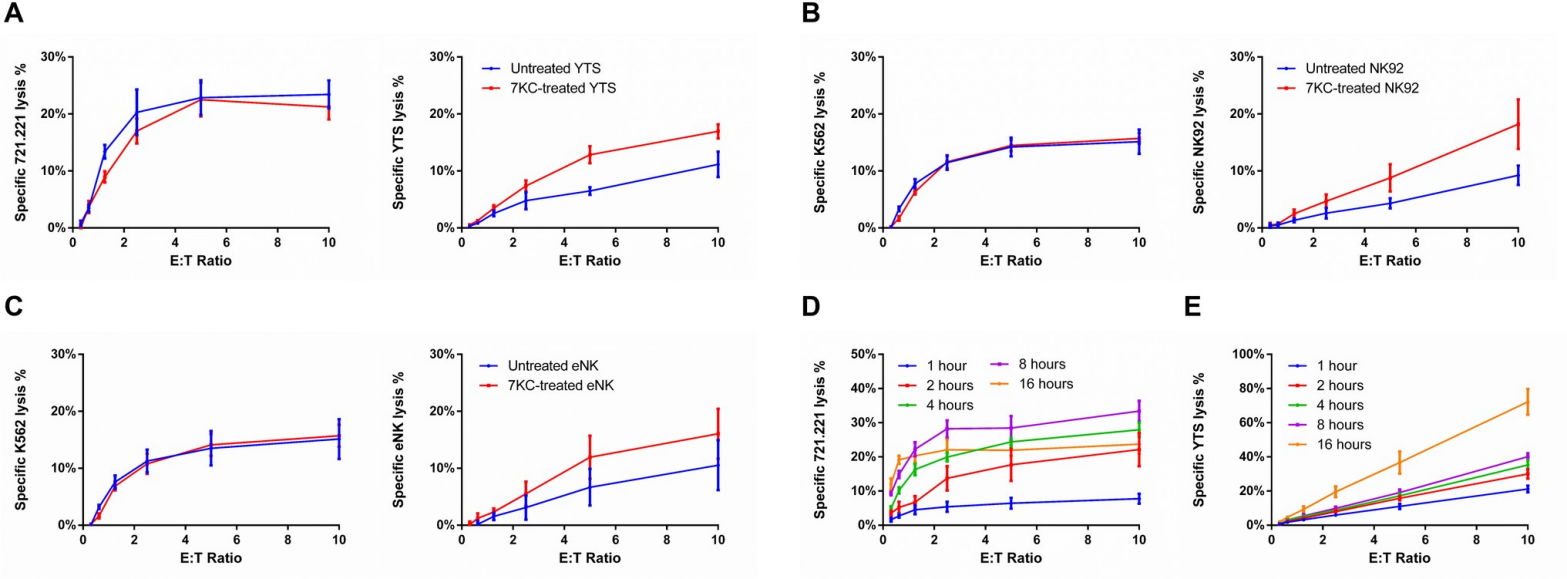
7KC treatment of NK cells induces NK cell death during target killing. Cytotoxic function (A, left, ns) and survival (A, right, *p* < 0.05) of untreated or 7KC-pretreated YTS cells incubated with 721.221 target cells were measured using 4-h ^51^Cr release assay. Similarly, cytotoxic function (B, left, ns) and survival (B, right, *p* < 0.05) of untreated or 7KC-pretreated NK92 cells incubated with K562 cells were measured by 4-h ^51^Cr release assay. To measure cytotoxic function in each case, target cells were ^51^Cr labeled (A, B, left), whereas to measure survival of NK cells, the NK cells themselves were ^51^Cr labeled (A, B, right), and for both, the ^51^Cr release was measured after 4 h of incubation. To validate the elevated death of 7KC-pretreated YTS cells, the cytotoxic function (C, left, ns) and survival (C, right, *p* < 0.05) of primary human purified eNK cells were measured after their labeling with ^51^Cr and when incubated with K562 cells with or without 7KC pretreatment. A correlation between NK cell survival and cytotoxicity after 7KC treatment was evaluated by varying the duration of incubation for assays measuring cytotoxicity (D) and survival (E) of 7KC-pretreated YTS cells incubated with 721.221 target cells using ^51^Cr-labeled NK or target cells, respectively. All values presented represent averages of 3 independent experiments (NK cell lines) or 5 distinct healthy donors and error bars display ± SD (A−E). Wilcoxon signed rank test was used for the comparison between curves. eNK, ex vivo NK; NK, natural killer; ns, not significant; 7KC, 7-ketocholesterol.

Since NK cells demonstrate a highly ordered synaptic lipid membrane, we asked if it could serve a role in NK cell survival during the cytolytic process. Thus, we next evaluated the influence of lipid packing on NK cell survival during cytotoxicity. NK cells were pretreated with or without 7KC, ^51^Cr labeled and incubated with paired target cells for 4 h, after which the survival of NK cells was evaluated via the release of ^51^Cr from dying NK cells. 7KC treatment of NK cells did not affect the lytic capability of NK cells, and both YTS and NK92 cells killed their respective target cells similarly to those not treated with 7KC (Fig 2A and 2B). 7KC-pretreated YTS and NK92 cells, however, had significantly elevated cell death rates compared to those in untreated cells (Fig 2A and 2B). The similarity of finding between 2 disparate NK cell lines suggests that the finding represents a consistent property of NK cells. That said, we also evaluated freshly isolated ex vivo human NK cells. As was found with the NK cell lines, 7KC pretreatment did not affect the lytic capability against, but did also lead to increased death of the NK cells during incubation with susceptible target cells (Fig 2C, individual donors shown separately in S3 Fig). Thus, 7KC induces NK cell death during target cell exposure, while not compromising the ability of NK cells to recognize and destroy a target cell. This suggests that lipid ordering in NK cells is required for their optimal survival during cytotoxicity.

Although the standard time frame used for an in vitro cytotoxicity assay is 4 h, target cell killing occurs within much shorter time frames and also continues after the 4-h time point. This represents a combination of NK cell access to target cells as well as eventually serial killing activity (which would theoretically benefit from NK cell survival). By varying the time of the cytotoxicity assay using 7KC-treated NK cells, we wanted to ask if the extent of target cell killing was related to that of autolysis. Thus, 7KC-pretreated YTS cells were incubated with 721.221 cells for different durations. The cytotoxicity and survival of YTS cells were measured based on the release of ^51^Cr from 721.221 cells and YTS cells, respectively. As expected, the killing of 721.221 target cells increased proportionally to the time of incubation with YTS cells in the cytotoxicity assay (Fig 2D; with the exception of the 16-h time point, which we presume to be a feature of extended ^51^Cr labeling of 721.221 cells). The death of NK cells, however, increased markedly during extended coincubations with target cells and was generally increased in concert with the time of exposure to 721.221 target cells (Fig 2E). The parallel increases in target cell exposure and NK cell death in these cytotoxicity assays could imply a degranulation-coupled mechanism underlying the 7KC-induced NK cell autolysis.

To validate the role of lipid packing and further rule out any unknown effect of 7KC, we also attempted an alternative experimental approach to manipulate membrane properties. We utilized Nystatin, which disrupts cholesterol-rich microdomains on plasma membrane without removing cholesterol from the membrane [29]. Thus, its effect would be expected to be distinct from 7KC in changing lipid composition. YTS cells were treated with Nystatin in varying concentrations and then evaluated for their ability to kill 721.221 target cells or for their survival when paired with 721.221 cells. Albeit with lower molar efficiency compared to 7KC, Nystatin treatment exhibited dose-dependent decreases in NK cell survival without affecting their ability to kill 721.221 target cells (S4A Fig). This extends the findings using 7KC and suggests a vital role for lipid packing in NK cell self-protection.

### 7KC-induced NK cell death requires target cell-induced NK cell degranulation

**Fig 3:**
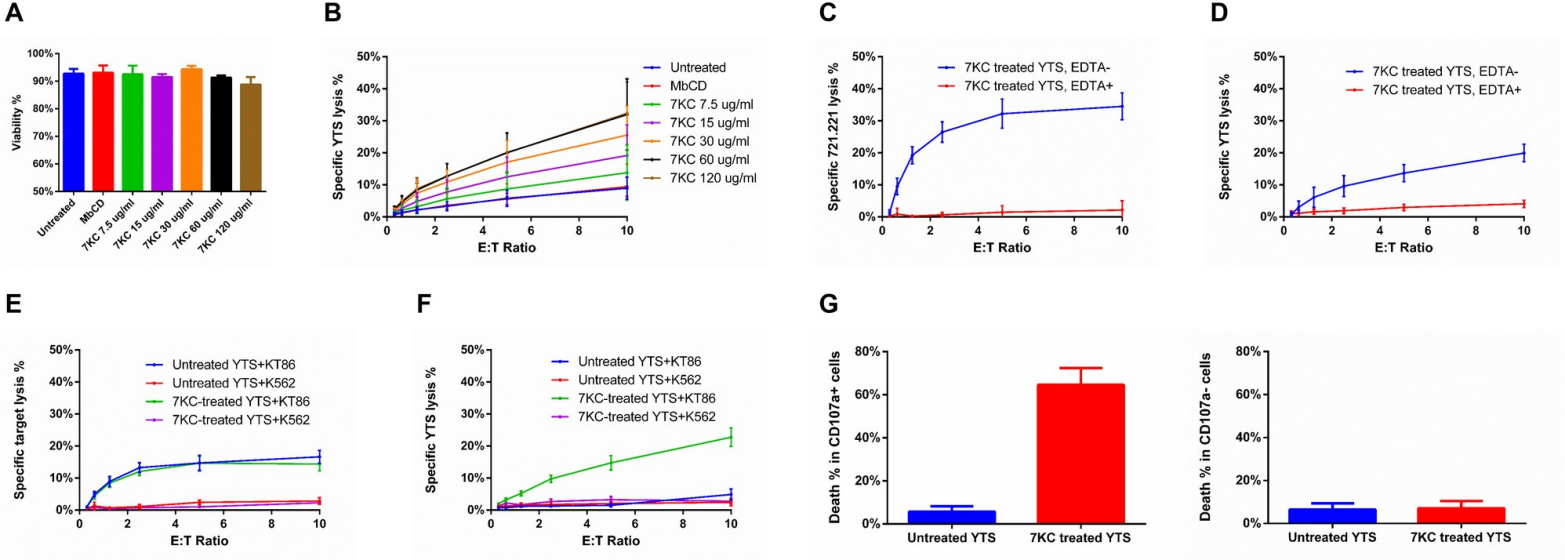
7KC-induced NK cell death requires target cell–induced NK cell degranulation. (A) Survival of YTS cells after a 4-h incubation in the absence of target cells over a range of 7KC concentrations was measured based on their uptake of PI by flow cytometry to evaluate any direct toxicity of 7KC. (B) The same range of 7KC concentrations was also used as pretreatment of ^51^Cr-labeled YTS cells that were incubated with 721.221 target cells over a range of effector to target cell ratios for 4 h. The release of ^51^Cr was measured in both sets of experiments at 4 h. Cytotoxicity (C, *p* < 0.05) and survival (D, *p* < 0.05) of 7KC-pretreated YTS cells, with or without EDTA (10 mM) calcium chelation, were measured after a 4-h incubation with ^51^Cr-labeled 721.221 target cells. Cytotoxicity mediated by (E, *p* < 0.05) and survival (F, *p* < 0.05) of 7KC-pretreated YTS cells were measured after a 4-h incubation with nontriggering K562 cells or triggering KT86 cells. Viability (G) of untreated and 7KC-pretreated YTS cells with or without up-regulated LAMP-1 after a 4-h incubation with 721.221 cells was evaluated using flow cytometry with PI used to detect cell death. All data represent averages from 3 independent experiments, and error bars show ± SD (A−E). Wilcoxon signed rank test was used for the comparison between curves. NK, natural killer; PI, propidium iodide; 7KC, 7-ketocholesterol.

Although 7KC has been reported to have no significant impact on T cell viability and signaling [28], we wanted to be certain that NK cell death was not due to any intrinsic toxicity of 7KC. Thus, YTS cells were pretreated with different concentrations of 7KC, and then cultured alone or with 721.221 cells for 4 h. Over a wide range of 7KC concentrations, the viability of YTS cells in the absence of target cells was unaffected by the drug or their activation state (Figs 3A, S4B and S4C). Other routine parameters such as cell size, number and performance in experiments were not affected by treatment. In the presence of target cells, however, 7KC caused a dose-dependent NK cell death (Fig 3B). Thus, it is likely that the completeness of blockade of lipid ordering in the NK cell presynaptic membrane is directly related to the degree of autolysis. Furthermore, this indicated that the death of 7KC-pretreated NK cells was linked to the presence of target cells and either to target cell-induced activation of NK cells or to the process of cytotoxicity itself.

To more specifically investigate the mechanism of 7KC-induced NK cell death, especially its linkage with the activation induced by target cells and ultimately degranulation, we measured the effect of 7KC treatment under calcium-depleted conditions. Calcium is critical in the signaling required for NK cell activation and is also necessary for lytic granule release as well as perforin activation and membrane binding [22]. Thus, we depleted calcium from medium using the chelator EDTA at concentrations previously defined as effective to inactivate perforin [30]. In the presence of EDTA, the cytotoxicity of YTS cells was completely abolished when evaluated by ^51^Cr release assays against 721.221 target cells, as has been previously reported for NK cells (Fig 3C). Interestingly, when YTS cells were labeled with ^51^Cr and combined with 721.221 target cells, EDTA also completely blocked the death of the 7KC-treated NK cells (Fig 3D). Thus, NK cell autolysis was prevented by chelation suggesting that NK cell activation, degranulation, or lytic molecule function is required for NK cell death in the presence of 7KC. This also largely excludes any direct NK cell toxicity of 7KC pretreatment as the autolysis of NK cells can be blocked by EDTA.

To further evaluate the dependency of 7KC-induced NK cell death upon activation by target cells, we utilized both triggering and nontriggering target cell pairs along with YTS cells. Unlike 721.221 target cells, K562 cells lack the surface expression of activation ligands needed by YTS cells to promote degranulation. YTS cells do conjugate with and adhere to K562 cells resulting in the generation of some signals, but the signals required for degranulation and cytotoxicity are not present. Thus, YTS cells form noncytolytic conjugates with K562 cells (S5 Fig) [31]. The expression in K562 cells of a ligand sufficient for triggering the degranulation of YTS, CD86, makes the K562 target cell susceptible to YTS killing. A K562 cell line stably expressing CD86 (KT86) are easily killed by cytotoxicity by YTS cells [31] and were used in comparison to K562 cells to further evaluate the mechanism of 7KC-induced NK cell autolysis. Cytotoxicity assays with ^51^Cr-labeled K562 or KT86 cells demonstrated the ability of YTS cells to kill the latter but not the former, neither of which were affected by 7KC pretreatment of the NK cells (Fig 3E). When the YTS cells were ^51^Cr labeled, only those pretreated with 7KC were killed and only when incubated with the KT86 cells (Fig 3F). This suggests that adhesion to a target cell was insufficient to cause NK cell autolysis in the presence of disrupted lipid ordering and that triggering for cytotoxicity was required. This, in concert with calcium chelation, suggests that active cytotoxicity with degranulation is prerequisite for autolysis and that lipid ordering might be protective. Furthermore, since the only difference between these experiments was the presence of the cytotoxicity triggering ligand, it is again unlikely that 7KC is in anyway intrinsically toxic to NK cells.

Since only a fraction of 7KC-pretreated NK cells underwent cell death when exposed to a susceptible target cell, we wanted to compare the dying NK cells to those that were still alive to try and gain insights into the mechanism underlying 7KC-induced NK cell death. Given that cytotoxicity and calcium were required for 7KC-induced NK cell autolysis, we speculated that NK cell degranulation would be associated. To assess this, we performed flow cytometry–based cytotoxicity assays in which 7KC-pretreated YTS cells were coincubated with 721.221 cells in the presence of medium containing propidium iodide (PI) as a viability indicator. After incubation, CD56^+^ cells were profiled via flow cytometry, and the percentage of cell surface LAMP-1, which denotes degranulation, was determined in the population of either viable or dying cells as marked by the absence or presence of PI, respectively. We found that when NK cells were pretreated with 7KC, those that expressed LAMP-1 were more frequently found to be dying (Fig 3G). In those that were not 7KC pretreated, LAMP-1^+^ NK cells were generally alive. As LAMP-1 is a marker of degranulation, and NK cells mediate cytotoxicity predominantly via directed secretion of perforin and proapoptotic proteases, it is likely that 7KC-induced NK cell death is dependent on the NK cell’s own killing machinery.

### 7KC-induced NK cell death is dependent on perforin

**Fig 4:**
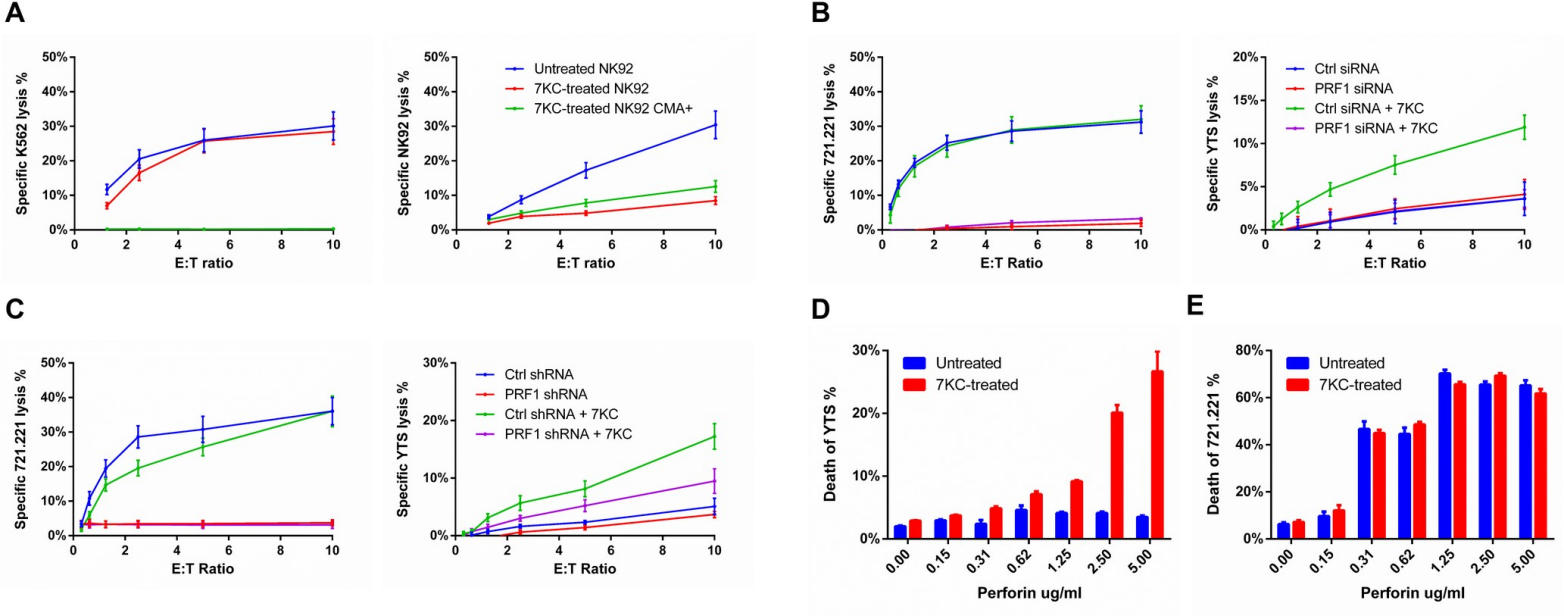
7KC-induced NK cell death is perforin dependent. Cytotoxic function mediated by (A, left) and survival of (A, right) untreated or CMA-treated NK92 cells, when incubated with K562 targets, were measured using 4-h ^51^Cr release assays. Cytotoxic function mediated by (B and C, left) and survival of (B and C, right) YTS cells, with or without knockdown of perforin induced by siRNA (B) or shRNA (C), were measured using 4-h ^51^Cr release assay. Sensitivity of untreated or 7KC-pretreated YTS cells (D) or 721.221 (E) to free perforin were compared based on the uptake of viability dye using flow cytometry. Data represent mean values from 3 independent experiments, and error bars display ± SD (A−E). CMA, Concanamycin A; NK, natural killer; shRNA, short hairpin RNA; siRNA, small interfering RNA; 7KC, 7-ketocholesterol.

The NK cell death resulting from 7KC pretreatment appears to be autolysis owing to its dependence upon both a cytotoxicity-triggering target cell and calcium as well as its correlation with LAMP-1 up-regulation. Therefore, we wanted to evaluate if it was indeed the NK cell lytic machinery that was responsible for autolysis after the blockade of presynaptic lipid ordering via 7KC. To evaluate this, we initially attempted to interfere with the function of the NK cell lytic granule by treating cells with Concanamycin A (CMA). CMA deacidifies the granules and depletes perforin by promoting its degradation [32]. Pretreatment of NK cells with CMA for 60 min depleted their perforin as demonstrated by the detection of protein via flow cytometry (S6 Fig). To determine if CMA treatment would have an impact on the 7KC-induced NK cell autolysis, we treated NK cells with CMA and measured cytotoxicity against ^51^Cr-labeled target cells, or NK cells that had also been treated with 7KC. As expected, CMA treatment of NK cells reduced target cell killing. There was also a similar reduction in 7KC-induced NK cell death after CMA treatment (Fig 4A). The incomplete reduction in cytotoxicity by CMA could be a feature of the residual, granule-independent perforin that would not be affected by granule deacidification [33]. CMA treatment, however, suggests a dependence of NK cell autolysis after 7KC treatment upon the lytic machinery itself.

Since perforin degradation via the deacidification of lytic granules may be associated with additional consequences [32], we utilized alternative approaches to consider a dependence of 7KC-induced autolysis upon the lytic machinery. We employed 2 different RNA interference techniques to specifically deplete perforin from NK cells and evaluate the impact of reducing perforin expression upon autolysis. PRF1-targeting small interfering RNA (siRNA) in YTS cells affected substantial decreases in perforin protein as measured by western blot and fluorescence microscopy analyses (S7A–S7D Fig). The same was found using PRF1-short hairpin RNA (shRNA)-transduced YTS cells when measured by flow cytometry and fluorescence microscopy analyses (S7E–S7H Fig). As would be expected, both of these approaches resulted in a complete loss of cytotoxicity against 721.221 target cells (Fig 4B and 4C). When YTS cells were ^51^Cr labeled and pretreated with 7KC, however, the perforin siRNA-treated or shRNA-transduced cells survived at levels similar to the YTS cells that were not 7KC treated (Fig 4B and 4C). Irrespective, 7KC-induced autolysis was dependent upon perforin and thus likely represents a susceptibility to perforin released during cytotoxicity mediated through the lytic synapse.

While perforin was necessary for 7KC-induced NK cell autolysis, we next asked if it was also sufficient on its own to kill NK cells having lipids that were disordered by 7KC treatment. To directly assess the impact of perforin upon the NK cell membrane and the effect of lipid ordering, we measured the sensitivity of NK cells to free perforin with or without 7KC pretreatment. YTS cells that were either 7KC pretreated or untreated were cultured in PI containing medium with various concentrations of perforin. Here, perforin-mediated transmembrane pore formation allows for the uptake of impermeable PI dye, and, thus, the sensitivity to perforin can be evaluated based on the percentage of PI-positive NK cells. When incubated with purified perforin, YTS cells demonstrated minimal PI uptake, which increased slightly with increasing perforin concentrations. When YTS cells were pretreated with 7KC, however, there was an increase in the perforin-induced PI uptake at all concentrations of perforin tested, which became increasingly noticeable at higher concentrations (Fig 4D). This difference was not observed in the same experiment performed with a susceptible target cell, 721.221 (Fig 4E), which demonstrated similar PI uptake at given concentrations of free perforin irrespective of 7KC treatment. Thus, 7KC treatment and presumably the resulting disordering of the otherwise increased ordered lipid in the NK cell membrane lead to a lack of protection against perforin.

### Membrane binding of perforin is blocked by densely packed lipid membranes

**Fig 5:**
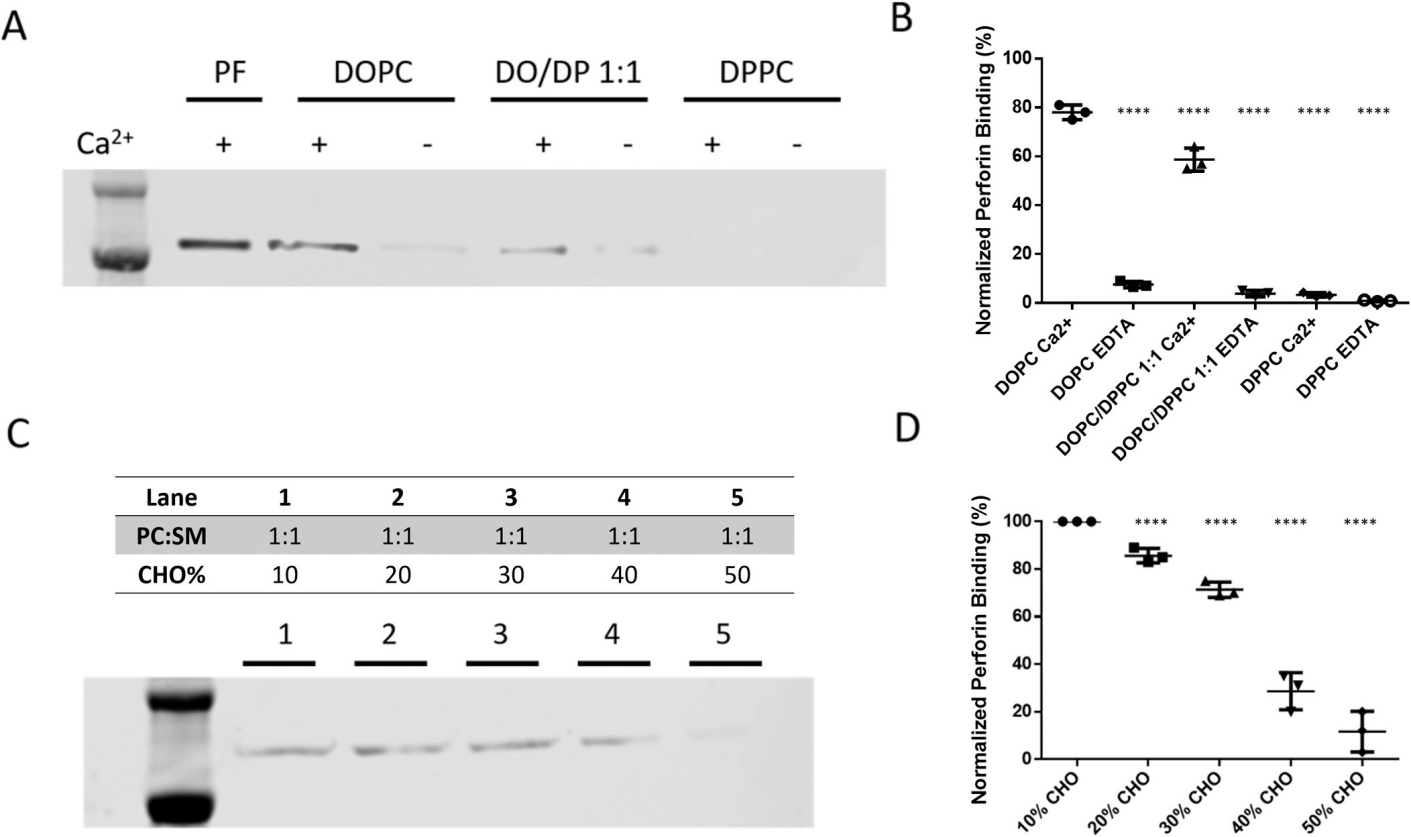
Perforin membrane binding is prevented by the presence of densely packed lipid. Differently packed liposomes were created by either pure or a ratio of DOPC and DPPC, which were coincubated with perforin. Levels of membrane-bound perforin were evaluated by western blot analysis (A, representative of 3 independent repeats). Since membrane binding of perforin requires the presence of Ca^++^, this was either added at 20 mM concentration or depleted as specified in the blot and quantification. As a control, pure perforin (PF) was evaluated without liposomes. Three independent experiments were performed, and the quantitative values for each via densitometry were plotted with mean ± SD shown (B). Using Student *t* test, the mean values were different from DOPC Ca^++^ *p* < 0.0001, **** (C) Differently packed PC:SM:CHO liposomes were created with 1:1 PC:SM and increasing percentages of CHO (as specified above each lane) and coincubated with perforin in the presence of Ca^++^. Levels of membrane-bound perforin were evaluated by western blot analysis using anti-perforin antibody (clone D48) (representative result shown). Three independent experiments were performed, and the quantitative values for each via densitometry were plotted with mean ± SD shown (D). Using Student *t* test, the mean values were different from 10% CHO *p* < 0.0001, ****. CHO, cholesterol; DOPC, dioleoyl phosphatidylcholine; DPPC, dipalmitoyl phosphatidylcholine; PC, phosphatidylcholine; SM, sphingomyelin.

To understand how 7KC treatment might contribute to sensitizing NK cells to perforin attack, we wanted to directly evaluate if the 7KC-induced disruption of densely packed lipid membranes could underlie the inhibition of perforin binding. Thus, we utilized a synthetic cell-free system and measured the binding of perforin to model membranes having different lipid packing densities. Liposomes were generated using dioleoyl phosphatidylcholine (DOPC) and dipalmitoyl phosphatidylcholine (DPPC). Both lipids share identical head groups, and, thus, any potential head group specificity for perforin binding can be excluded. The difference between the 2 lipids, however, is in their tails. DOPC contains 2 unsaturated fatty acid sidechains that generate a more fluid, disordered lipid membrane, and the saturated sidechains of DPPC tend to form densely packed, high lipid order membranes (at 37°C). By adjusting the ratio of DOPC/DPPC, liposomes with a range of packing densities were prepared, which were validated using fluorescent packing sensors (S8A and S8B Fig). To determine the impact of membrane lipid composition on perforin binding ability, differently packed liposomes were incubated with perforin, and then membranes were precipitated by centrifugation. These liposome preparations were then evaluated by western blot analysis to determine the amount of membrane-bound perforin. Because perforin activation and membrane binding is dependent upon calcium, it was intentionally depleted in control groups to assess any nonspecific binding of perforin. When compared to liposomes constructed of 100% DPPC, which had the most packed and ordered membrane, liposomes with higher percentages of DOPC exhibited higher perforin binding (Fig 5A and 5B). The pure DPPC membrane retained the least amount of perforin, and a 1:1 mixture of the 2 lipids was intermediate. Thus, highly ordered membranes tend to be resistant to perforin binding.

Although the identical polar head group is a benefit of the DOPC/DPPC model membranes system, these are physically quite distinct from cell membranes [34]. In order to better mimic the plasma membrane of NK cells, liposomes were constructed that consisted of phosphatidylcholine (PC), sphingomyelin (SM), and cholesterol. Using this more physiological system, the amount of cholesterol could be varied to change the degree of lipid ordering. To validate this approach, liposomes were generated using a constant amount of PC and SM, but with cholesterol ranging from 10% to 50%. The degree of lipid ordering was measured using a fluorescent membrane sensor and the degree of lipid packing correlated with the percentage of added cholesterol (S8C and S8D Fig). Perforin was then added to these liposomes, membranes precipitated by centrifugation, and the amount of perforin retained, evaluated by western blot analysis. The cholesterol concentration inversely correlated with the degree of perforin retention by the liposomes (Fig 5C and 5D). In concert with our findings from the DOPC/DPPC model membrane, this suggested that the degree of membrane lipid ordering governed the protection from perforin insertion. Thus, the mechanism of NK cell protection from perforin is likely due to a blockade of perforin binding by densely packed presynaptic membranes.

### Lytic granule membranes reinforce local membrane packing at sites of NK cell degranulation

Increased lipid ordering at the presynaptic membrane was found to be critical for NK cell protection against secreted perforin during degranulation. That said, the concentration of perforin is likely to be highest where the release of perforin occurs. Thus, we wanted to evaluate the lytic granule itself in consideration of special membrane properties it might possess that could potentially contribute to the protection of NK cells from autolysis. In particular, we wanted to consider the possibility that the previously described fusion of the lytic granule–cell membrane with that of NK cell membrane that occurs during degranulation [35] may itself alter the composition and packing status of presynaptic membrane.

**Fig 6:**
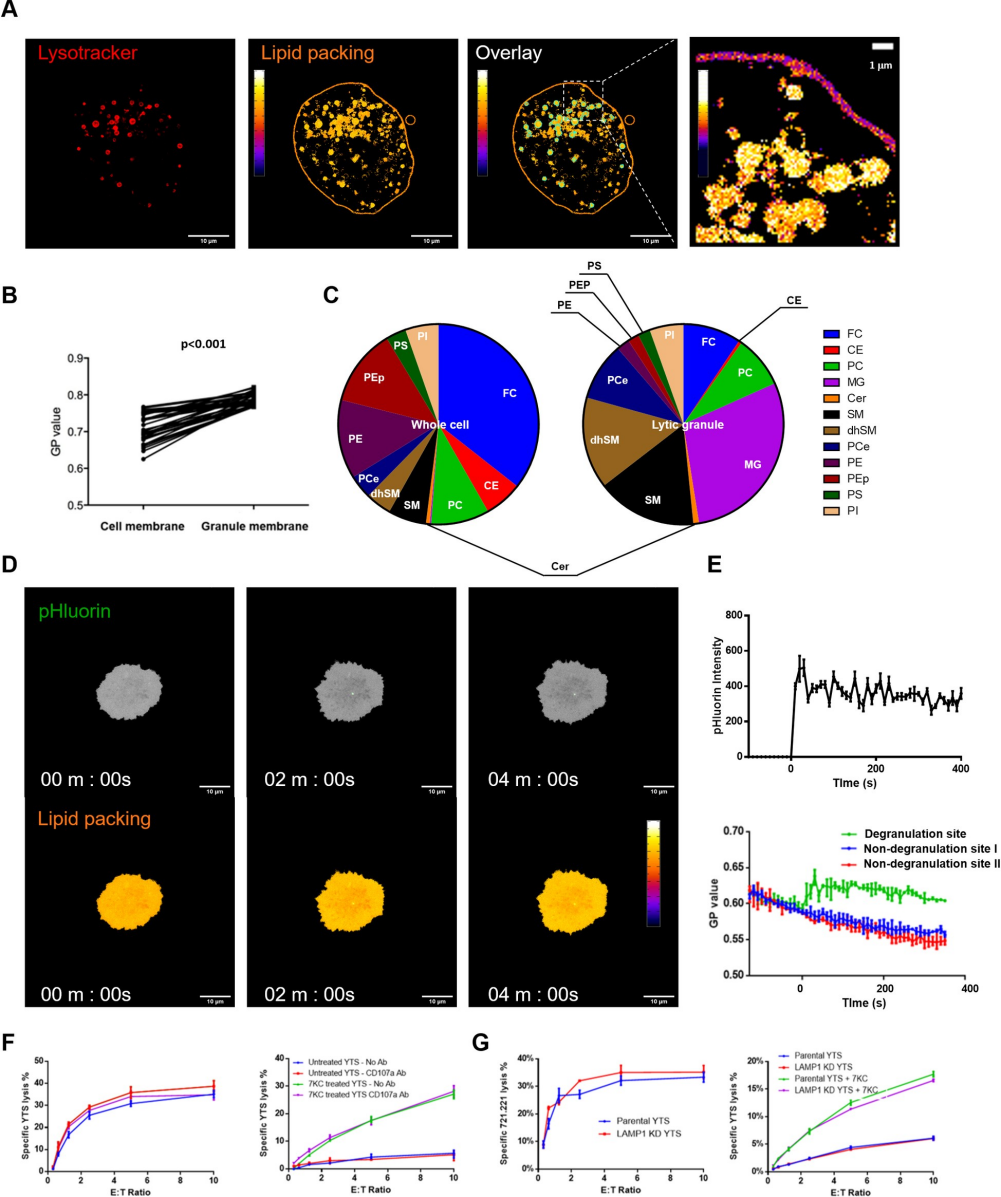
Lytic granule membranes are highly ordered and enhance local membrane packing at sites of NK cell degranulation. (A) Lipid packing densities of the cell membranes of nonstimulated YTS cell were measured with the fluorescent packing sensor Laurdan (pseudocolor scale, middle), and lytic granules were identified using lysotracker dye (red, left). The overlay of the 2 fluorescent channels was used to identify lytic granules (using an object identifier in ImageJ software–outlined in teal), which allowed for the measurement of lipid packing density at the location of the lytic granules (pseudocolor scale). An enlargement (right) shows the lipid packing of lytic granule membranes and cell membrane with an expanded pseudocolor scale using a more refined range to amplify lipid packing differences. (B) Lipid packing density was quantified in the region of the lytic granules as determined by lysotracker colocalization as the GP value of this colocalized region and was compared to the GP value of the entire cell membrane using a paired *t* test (*n =* 15 cells from 3 independent experiments, *p* < 0.001). (C) Lipid composition of whole YTS cell membranes and isolated YTS lytic granule membranes were determined by LC–MS and were plotted in pie charts. The results represent 2 independent experiments, and individual lipids are shown in different colors with the following major lipids depicted FC, PE, CE, SM, dhSM, MG, PEp, PC, PI, PCe, PS, and Cer. (D) YTS-LAMP1-pHluorin cells were used to identify the sites of degranulation at the NK cell membrane on an activating glass surface (coated with anti-CD18 and anti-CD28 antibodies) via live TIRF microscopy. Images from a representative time lapse were selected to denote the presence of pHluorin (green, top), which signifies the site of degranulation. For reference, the entirety of the cell on the glass is depicted in gray. The lipid packing signal using a pseudocolor scale is shown in the image below. (E) The emergence of pHluorin signal was monitored using TIRFm in cells that had been preloaded with the Laurdan lipid packing sensor, and local membrane packing at degranulation sites was quantified and compared with randomly selected non-degranulation sites. The mean values for 10 independently measured cells are shown ± SD. (F) Cytotoxicity mediated by and survival of 7KC-pretreated YTS cells as measured by Cr release assays in the presence of LAMP-1 blocking antibody. (G) Cytotoxicity mediated by and survival of 7KC-pretreated YTS cells in which LAMP-1 expression was reduced by prior siRNA introduction. Cytotoxicity data represent averages of 3 replicated wells (i.e., technical repeats), and error bars display ± SD of these technical replicates. (F, G). CE, cholesteryl ester; Cer, ceramide; dhSM, dihyodrosphingomyelin; FC, free cholesterol; GP, generalized polarization; LC–MS, liquid chromatography–mass spectrometry; MG, monoglycerol; NK, natural killer; PC, phosphatidylcholine; PCe, alkylacyl phosphatidylcholine; PE, phosphatidylethanolamine; PEp, phosphatidylethanolamine-based plasmalogen; PI, phosphatidylinositol; PS, phosphatidylserine; siRNA, small interfering RNA; SM, sphingomyelin; TIRF, total internal reflection fluorescent; 7KC, 7-ketocholesterol.

To investigate potential influence of lytic granule–cell membrane fusion on presynaptic membrane properties, we first evaluated the lipid properties of the lytic granule membranes themselves. We initially used live cell confocal microscopy and NK cells incubated with a cell permeable packing sensor (Laurdan) to define lipid packing along with lysotracker to identify lytic granules. Organelles that fluoresced in the presence of lysotracker interestingly appeared to have densely packed membranes (Fig 6A). When quantified, the lytic granule membranes had higher lipid order than the NK cell presynaptic membrane as indicated by their comparative GP values (Fig 6B). This suggested that the lytic granules of NK cells have endogenous highly ordered lipid. Since these analyses were performed in resting YTS cells, it suggests that the lytic granules might be characterized by an inherently distinct lipid composition. In order to evaluate this, we isolated lytic granules from YTS cells and compared those to isolated total YTS cell membranes (which also include those of lytic granules). Both membrane preparations were then analyzed with liquid chromatography–mass spectrometry. The lipid profiles from the membranes indicated a dramatic enrichment of SM and its derivatives as well as other liquid ordered membrane components in the lytic granules (Fig 6C, S1 Table). SM and derivatives are known to facilitate aliphatic tail alignment and thereby enhance the packing density of lipid membrane [36]. Of note, monosialodihexosylganglioside (GM3), a high melting point lipid enriched in lipid-ordered membranes [37,38], was increased over 20-fold in the granules (S1 Table). Thus, the lytic granule appears to be defined by a highly specialized lipid membrane.

Since the lytic granules had highly ordered membrane with SM-enriched composition, it was possible that lytic granule–cell membrane could reinforce the packing density of plasma membrane at sites of degranulation via membrane fusion. To determine if there were degranulation-induced changes in local synaptic membrane packing, we utilized NK cells expressing LAMP1–pHluorin as a degranulation indicator to define the precise site of lytic granule fusion [39] and prelabeled the cells with a lipid packing sensor. These NK cells were incubated on an activating glass surface containing antibodies against NK cell activation receptors that promote degranulation and imaged by TIRF microscopy. Local lipid packing changes were recorded at specific sites of lytic granule fusion as detected by the emergence of pHluorin fluorescence. At the time pHluorin signal was detected, a rapid rise of lipid order was also found at the degranulation site (Fig 6D). Interestingly, this increase of lipid ordering was sustained and spatially restricted to the degranulation site without a sign of diffusion over a 5-min time frame (Fig 6E). This may be explained by the low lateral diffusion rate of lipid molecules in a densely packed presynaptic membrane [40] and is consistent with the previous definition of prolonged granule transmembrane molecule patches in the NK cell synaptic membrane [39]. Given the protection provided by ordered membrane, this further increased ordering upon degranulation may provide enhanced defense at degranulation sites where NK cells face maximum local concentrations of secreted perforin. Thus, lytic granules themselves have an endogenously densely packed membrane, and after degranulation contribute to focal reinforcement of presynaptic membrane lipid packing through lytic granule–cell membrane fusion to potentially provide additional protection where it is likely needed most.

Given the enhanced protection against autolysis provided by degranulation itself, we wanted to evaluate the possibility that protective receptors externalized by lytic granules could be contributing. LAMP-1 comprises 50% of all lysosomal membrane proteins [41], is externalized upon degranulation, and has been previously proposed to provide protection against perforin attack under certain circumstances [16]. To consider an independent and protective role of LAMP-1 externalization, YTS cells were untreated or 7KC pretreated and coincubated with 721.221 cells in the presence of medium containing LAMP-1-neutralizing antibodies. Blockade of LAMP-1 did not interfere with NK cell killing of ^51^Cr-labeled 721.221 target cells, nor did it cause autolysis of ^51^Cr-labeled YTS cells in the presence of 721.221 target cells (Fig 6F). Pretreatment with 7KC did not change these results, suggesting that lipid order was the primary influence upon degranulation-induced autolysis. Since LAMP-1-bound antibodies could in theory dampen perforin binding by steric hindrance effects, we utilized siRNA as an alternative approach to substantially reduce LAMP-1 expression in NK cells globally and inside the lytic granules specifically (S9 Fig). Despite the reduction in LAMP-1 expression by siRNA treatment, there were no changes in NK cell survival by ^51^Cr release assay using labeled NK cells (Fig 6G). Furthermore, there was no amplification of autolysis in 7KC-treated YTS cells that had also been subject to LAMP-1 siRNA treatment. Thus, while LAMP-1 is abundant in lytic granules and is externalized in the region of a degranulation, the lipid ordering of the presynaptic membrane appears to be a more impactful protective mechanism utilized by NK cells against secreted perforin.

### Cancer cells can evade NK cell killing by generating densely packed postsynaptic lipid membranes

The breast cancer cell line MB-231 has been reported to evade NK cell killing via a rapid and massive accumulation of F-actin near the immunological synapse. This actin response was associated with a reduced uptake of granzyme B by the MB-231 cells and a lower rate of apoptosis [42]. Since in immune cells synaptic actin accumulation has been linked to lipid raft aggregation, and because perforin is required for efficient granzyme B delivery to target cells, we hypothesized that MB-231 cells form postsynaptic densely packed membranes and thereby evade NK cell killing by preventing perforin function.

**Fig 7:**
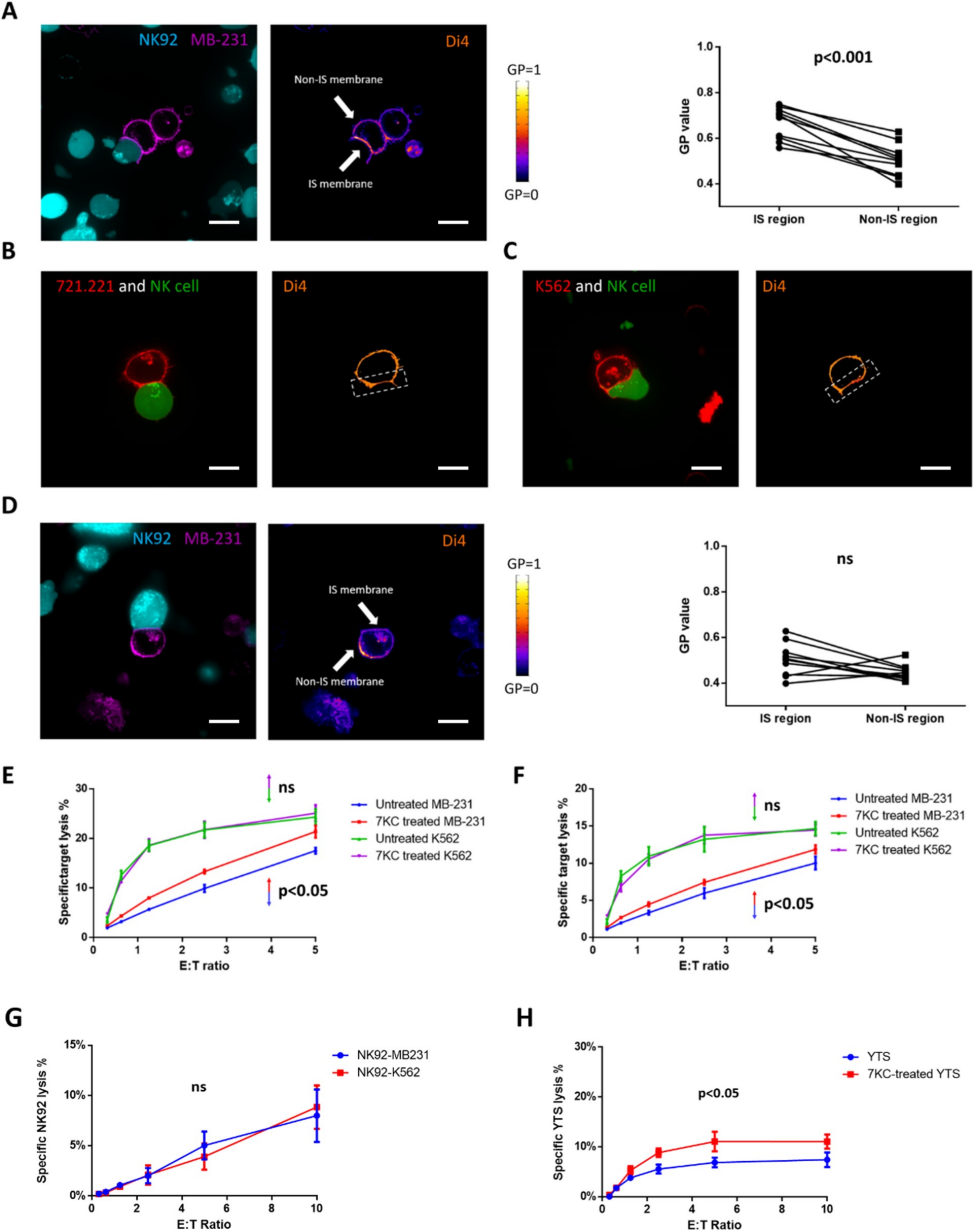
Cancer cells can evade NK cell killing by generating densely packed postsynaptic lipid membranes. The lipid packing of target cell membranes in Di4-ANEPPDHQ (Di4) fluorescent packing sensor labeled MB-231 cells (A, left), 721.221 cells (B), K562 cells (C), and 7KC-pretreated MB-231 cells (D, left) conjugated with calcein green–loaded YTS or NK92 cells, respectively, was measured using confocal microscopy. Representative images of 15–30 conjugates over 3 independent experiments are shown. In each image pair, the left image shows the effector cell demonstrating calcein green fluorescence (A, teal; B, green; C, green; D, teal) and target cell demonstrating cell mask fluorescence (A, purple; B, red; C, red; D, purple). The right image shows target cells are overlaid with a pseudocolor scale (A–D) to demonstrate differences in packing and density. Example synaptic regions were denoted by arrows (A) or boxes (B, C) in the images. The GP value was measured in independent experiments (*n =* 10 for untreated MB-231 and *n* = 10 for 7KC-pretreated MB-231), and a paired *t* test was used for statistical comparison between the individual values for synaptic and nonsynaptic regions (A, right and D, right). (E, F) 7KC was used to disrupt membrane packing in either K562 (purple line) or MB-231 (red line) target cells and were incubated with NK92 cells (E) or human NK cells (F) and evaluated in a 4-h ^51^Cr release cytotoxicity assay. The specific lysis of 7KC-treated target cells were compared to that of untreated MB-231 (blue line) or K562 (green line), and the *p*-value shown is for the MB-231 (*p* < 0.05) or K652 (*p* > 0.05, ns) target cells. (G) Survival of NK92 cells incubated with nonresistant targets K562 or resistant targets MB-231 was measured by 4-h ^51^Cr release assay. (H) Survival of untreated or 7KC-pretreated YTS cells incubated with NK92 cells was measured by 4-h ^51^Cr release assay in which the YTS cells were ^51^Cr labeled. Data represent results from 3 independent experiments using NK cell lines or eNK cells from 3 healthy donors and error bars show ± SD (E−H). Wilcoxon signed rank test was used for the comparison between curves. Scale bar: 10 μm. eNK, ex vivo NK; GP, generalized polarization; IS, immunological synapse; NK, natural killer; ns, not significant; 7KC, 7-ketocholesterol.

To evaluate this possibility, we first characterized the lipid packing density of the MB-231 cell membrane by prelabeling it with a lipid packing sensor and measuring the lipid density via live cell microscopy. To allow for the formation of synapses, lipid sensor prelabeled MB-231 cells were incubated with NK92 cells. An accumulation of densely packed MB-231 cell membranes was detected at the immunological synapse region formed with NK92 cells with there being relatively less packed membrane at the nonimmunological synapse region, as well as in unconjugated MB-231 cells (Fig 7A). This distribution of lipid ordering was at very similar levels to those we had observed with NK cells and thereby should provide some protection against perforin binding. Notably, this highly ordered postsynaptic membrane was not detected with other NK-susceptible target cell lines, such as 721.221 and K562 (Fig 7B and 7C). Thus, the active accumulation of highly ordered lipid membrane appeared to be a characteristic of the MB-231 NK cell resistant target.

To determine if the increased lipid packing at the postsynaptic membrane was relevant to protection of resistant target cells from perforin-mediated killing, we treated the MB-231 cells with 7KC and then evaluated their susceptibility to NK92 cells. 7KC pretreatment disrupted postsynaptic membrane packing of MB-231 cells, creating evenly distributed low lipid order in their cell membrane (Fig 7D). We then measured the ability of NK cells to kill ^51^Cr-labeled MB-231 cells with or without 7KC pretreatment. Only those that were 7KC pretreated had increased susceptibility to death mediated by either NK92 cells (Fig 7E) or primary human NK cells (Fig 7F). Thus, the same mechanism underlying protection against perforin-mediated death used by NK cells seems to also be employed by MB-231 cells to evade perforin attack. For the NK-susceptible target cell lines, K562, their susceptibilities to NK92 killing were not influenced by 7KC pretreatment (Fig 7E and 7F), which is consistent with their evenly distributed membrane packing. Interestingly, NK92 cells paired with MB-231 target cells did not have decreased survival as measured cytotoxicity assays in which the NK92 cells were ^51^Cr labeled instead of the MB-231 cells (Fig 7G). This suggests that despite the target cell being relatively resistant to NK cells and having a more highly ordered postsynaptic membrane that the resistance of the highly ordered presynaptic NK cell membrane remains intact.

Since our NK cell lines are also derivative from NK cell malignancies [43], and we have learned that they possess higher baseline lipid ordering like ex vivo NK cells do, we wanted to evaluate them as an additional malignant cell that might use lipid ordering to resist NK cell killing. It also provides some perspective into the concept of fratricide, where one NK cell kills another, although in this case, one NK cell line is likely recognizing the other as a target. When YTS cells were used as target cells and labeled with ^51^Cr in a 4-h assay, NK92 cells were able to mediate some killing (Fig 7H). When the YTS cells were treated with 7KC, however, there was a doubling of total percentage of YTS cells killed and a 2-fold increase in killing efficiency (the same percentage of 7KC-treated YTS cells were killed at an effector to target cell ratio of 1.25:1 as were untreated YTS cells at an effector to target cell ratio of 2.5:1). Therefore, lipid density represents a means for resistance to perforin: one that provides protection for NK cells from autolysis upon degranulation and potentially fratricide and one that can be adopted by NK cell resistant target cells to potentially contribute to their evasion of NK cell-mediated defense.

## Discussion

Cytotoxic lymphocytes including NK cells and CTLs are fundamental to immune defense in large part through their ability to mediate the killing of target cells identified as being dangerous. The cytolytic destruction of targets cells by NK cells is characterized by a series of tightly regulated stepwise cellular events creating a lytic immunological synapse [1]. This process allows for the creation of a synaptic cleft into which cytotoxic effector molecules are delivered by lytic granules. Within lytic granules, perforin is a pore-forming protein that is capable of inserting into target cell membranes to facilitate target killing. Perforin enables delivery of proapoptotic serine proteases to trigger target cell–programmed death [6]. After delivering a lethal hit, NK cells detach and can undergo sequential engagement with adjacent targets and even mediate serial killing. Due to their potent cytolytic capacity, precise regulation of the pore-forming activity of perforin is vital. Specifically, NK cells theoretically share the same vulnerability as their target cells but are seemingly resistant to the lytic molecules released upon degranulation [9]. Thus, NK cells must possess a robust protective mechanism against secreted perforin for them to sustain their survival as well as their known ability to affect serial killing (which may likely and directly benefit from increased protection from autolysis). Here, we propose that it is actually the membrane lipid ordering of NK cells that protect them from autolysis upon degranulation. Furthermore, we present experiments showing that lytic granules themselves contain highly ordered lipid and create local patches of lipid raft at the site of degranulation where this protection on the NK cell membrane is likely needed most. In other words, NK cell degranulation augments a perforin-resistant “shield.”

Although many biochemical and biophysical studies have been conducted to understand the inactivation of perforin inside of NK cells, it has remained unclear how NK cells protect themselves against the attack of secreted perforin that is released into the synaptic cleft. Firstly, it has been documented that both NK cells and CTLs do have relatively resistant membranes when compared to their common target cells. Specifically, an approximately 10-fold higher threshold of free perforin in resting NK and CTL membranes was observed using membrane patch clamp techniques [44]. We have also observed this phenomenon when comparing the baseline resistance to free perforin between the NK cell line YTS and the target cell line 721.221. Furthermore, a recent study reported a similar conclusion in CTLs and target cell lines EL4 and P815 [17]. Collectively, this suggests that NK cells do have intrinsically higher resistance to free perforin compared to their common target cells. Despite this, however, NK cells [45] and CTLs [12,46] are still susceptible to perforin attack from other effector cells, or fratricide. In a not exactly parallel experiment, we found that NK92 can kill YTS cells in coincubation experiments, and the activity is increased by pretreatment of the YTS cells with 7KC. This suggests that the inherent resistance of NK cells is intermediary between susceptible target cells and that which can be further induced via self-protective mechanisms against secreted perforin, specifically through additional lipid ordering at the synaptic platform and likely further still at sites of degranulation.

Several hypotheses have been proposed to explain an acquired resistance of NK cells to perforin-mediated autolysis during target engagement. It has been suggested that granule-derived cathepsin B brought to the cell surface provides self-protection for cytotoxic lymphocytes [13]. The protective function of cathepsin B, however, is questionable since CTLs from cathepsin B knockout mice have normal survival post-degranulation, indicating that this protease is at least dispensable for perforin reinsertion resulting in autolysis [14]. It has also been proposed that LAMP-1 might facilitate the directional delivery of perforin to target cells and protect an NK cell from perforin [16]. Recent work [17], however, and our findings (Fig 6) demonstrate that LAMP-1 can be dispensable for protection against perforin-mediated autolysis. Moreover, while a membrane-bound focal protein might protect during the initial degranulation event, it would be unlikely to be as helpful as the perforin is distributed throughout the synaptic cleft. As only less than 5% of NK cells and CTLs undergo autolysis during target engagement (Fig 2) [9], NK cells must utilize a robust, stable mechanism that both protects against the degranulation event as well as the presence of perforin in the synaptic cleft.

Since hydrophobic interactions are charge sensitive, we hypothesized a role for high lipid order membranes, also known as liquid-ordered membranes or lipid rafts, which have been previously shown to aggregate at the NK cell synapse [18–20,26]. Specifically, that as high lipid order membranes are known to affect charge distribution [23] on the presynaptic membrane, that perforin binding to the NK cell synaptic membrane with high lipid order would be inhibited. As a proof of concept, we used 7KC, a cholesterol derivative that disrupts lipid packing, to modulate membrane packing density in NK cells (we also validated the finding with Nystatin, which also disrupts lipid packing but via a different mechanism). Combined with high-resolution microscopy to measure NK cell lipid ordering, we demonstrated a role for local lipid environment change in protection against perforin. During target cell engagement, NK cells form a presynaptic membrane with high lipid order and 7KC-induced disruption of membrane packing sensitized NK cells to perforin-mediated autolysis after degranulation.

We also validated this concept using a reductionist model membrane system and observed that high lipid order liposome membranes were resistant to perforin binding. Thus, NK cell lipid membrane packing on the NK cell side of the synaptic cleft shields NK cells against perforin. This protection is augmented in several important ways, one of which is the coalescence of lipid rafts, which is otherwise essential in promoting downstream signaling for degranulation. Notably, this self-defense mechanism is temporally coupled with perforin secretion in that the local increase in these protective membranes is required for and thereby precedes degranulation. As such, this could guarantee that an NK cells is well prepared prior to degranulation for the potential consequences of exposure to perforin.

Another means through which NK cells augment their protection against their own perforin appears to be via the lytic granules themselves. The NK cell lytic granules harbor a highly cytotoxic molecule arsenal, which has historically been viewed as restrained via their unique chemical and physical environment. This includes a carefully maintained acidic pH to allow the safe storage of perforin and other cytotoxic cargoes [47]. As a deadly carrier, the maturation movement and release of lytic granules is tightly controlled [48]. That said, upon degranulation, the pH of the granule environment changes rapidly and the toxic molecules are activated. In theory, this puts the NK cell at risk of autolysis, especially at the site of degranulation where these molecules would be extremely close to the NK cell surface. We describe a feature of lytic granules that may help explain why NK cells do not die upon degranulation. Specifically, that NK cell lytic granules have endogenously densely packed lipid membranes, which may serve 2 purposes. First, it might provide additional capacity for safe storage of perforin inside lytic granule, especially as the granule matures or as pH fluctuates. Second, it provides a local and further increased lipid density at the site of degranulation, which might serve to additionally protect the NK cell from the release of perforin.

We analyzed lytic granules using lipidomics and defined an inherently distinct lipid composition, one characterized by dramatic enrichment of SM and its derivatives. These lipids are known to facilitate aliphatic tail alignment and thereby enhance the packing density of lipid membranes [36]. Since the lytic granule has a highly ordered membrane, we evaluated the possibility that lytic granule–cell membrane fusion itself reinforced the packing density of plasma membrane at the very sites of degranulation. By combining a degranulation indicator with a lipid packing sensor, we recorded a rapid rise of lipid order at the specific site of degranulation. Since perforin is concentrated in the lytic granule core and diffuses from it upon degranulation [49], a successful protective mechanism must be able to handle a local “hot spot” where NK cell faces maximum local concentrations of secreted and active perforin. Since the increase of lipid ordering was spatially restricted to the degranulation site without diffusion or diminution over a 5-min time frame, it serves the function of providing spatially stable, locally enhanced protection against perforin at the specific sites of degranulation where NK cells likely needed it most. In other words, degranulation results in super-ordering of lipid in the context of an already highly lipid-ordered presynaptic membrane.

The mechanisms identified in how NK cells protect themselves also have applicability to understanding how certain other cells may be “resistant” to NK cell killing. In particular, a variety of escape mechanisms have been identified in cancer cells that mediate their resistance to NK cell or CTL attack [50,51]. Here, we extend these to include the lipid density protection employed by NK cells themselves. The breast cancer cell line MB-231 has been described to evade NK cell killing via its own rapid and massive accumulation of F-actin at the immunological synapse [52]. Since in immune cells synaptic actin accumulation has been linked to lipid raft aggregation [53], we hypothesized that MB-231 cells form postsynaptic densely packed membranes and thereby evade NK cell killing by preventing perforin function. MB-231 cells accumulated densely packed membranes at the immunological synapse formed with NK cells measured using lipid sensors. This distribution and concentration of lipid ordering was similar to what we observed in NK cells and thus should be protective against perforin binding. Interestingly, the pairing of NK cells with these resistant more highly membrane lipid-ordered target cells did not result in a significant increase of NK cell death, suggesting that the self-protective mechanism of an NK cell remains effective even when facing a similarly dense postsynaptic membrane. In other words, there are likely some inherently perforin-resistant aspects of lipid-ordered membranes even in the face of exposure to perforin. Importantly, when treated with 7KC, MB-231 cells lost their postsynaptic lipid membrane packing and became susceptible to death mediated by NK92 cells. As the NK cell lines used in our work are derivative from human NK cell tumors, it also stood to reason that as target cells, they should also utilize this mechanism given what we had learned about NK cell lipid ordering [54]. When using the YTS NK cell line as a target cell, we found that it shared some of the lipid-dependent resistance properties of the MB-231 cells, thus extending the finding beyond just a single NK cell–resistant tumor.

## Materials and methods

### Ethics statement

All human samples were obtained and used in accordance with the Declaration of Helsinki with the written and informed consent of all participants under the guidance of the Baylor College of Medicine, or Columbia University Irving Medical Center Internal Review Boards for the Protection of Human Subjects.

### Cell lines, primary human NK cells, and lipid manipulation

The NK cell line YTS and target cell lines 721.221 and K562 were maintained in complete RPMI medium with 10% fetal calf serum (R10) [55]. The NK cell line NK92 was maintained in MyeloCult medium supplemented with 100 U/mL IL-2 (Roche, Basel, Switzerland). LAMP-1–pHluorin–transduced YTS cells were generated as described [39]. Human primary NK cells were isolated from healthy donor peripheral blood by Ficoll-Paque density centrifugation and RosetteSep human NK cell enrichment kit (Stemcell Technologies, Vancouver, Canada). For membrane packing manipulation, 15 mg/ml 7KC (Avanti Lipids, Alabaster, AL) or cholesterol (Avanti Lipids, Alabaster, AL) stock solution in ethanol was added to a methyl-β-cyclodextrin (Sigma-Aldrich Burlington, CA) solution with 50 mg/ml in PBS at 80°C to a final sterol concentration of 1.5 mg/ml. A volume of 15 μl of the lipid solution was then added to 1 ml of cell medium containing 5 × 10^5^ cells at 37°C for 30 min.

### Live cell microscopy

YTS and NK92 cells were incubated with 1 μM of the lipid packing sensors Laurdan or Di4-ANEPPDHQ (Di4, Thermo Fisher Scientific); and 721.221 and K562 target cells were stained with 1 μM of calcein green (Thermo Fisher Scientific, Carlsbad, CA) all for 30 min at 37°C and then washed twice with warm phenol red-free RPMI 1640 medium. For confocal microscopy, 8-well chamber slides were precoated with 5 mg/ml of mouse anti-human CD48 Ab (Clone TU145; BD Biosciences) for 60 min at 37°C and washed with warm phenol red-free RPMI 1640 medium to remove unbound Ab. The effector and target cells were mixed in 8-well chamber slides at a 2:1 ratio and immediately placed on prewarmed microenvironment chamber for confocal imaging. After 15 min of waiting for synapse formation, conjugates between the NK cells and target cells were imaged on a Zeiss Axio Observer Z1 inverted microscope outfitted with a Yokogawa CSU-X spinning disc with a 63×/1.4 NA objective. For TIRF microscopy, only NK cells were used. Here, 8-well chamber slides were precoated with 5 mg/ml of activating Ab (anti-CD28 and anti-CD18 for YTS cells, anti-NKp30 and anti-CD18 for NK92 cells) for 60 min at 37°C and washed with warm phenol red-free RPMI 1640 medium to remove unbound Ab. NK cells were seeded in these precoated 8-well chamber slides and immediately placed in a prewarmed microenvironment chamber for TIRF imaging using a GE DeltaVision OMX-SR microscope using a 60×/1.42 PlanApoN objective. After 5 min of waiting for cell spreading, presynaptic membranes of fully spread NK cells were imaged. Acquired images were analyzed and quantified using ImageJ or the Fiji version of ImageJ.

### ^51^Cr release assay

To measure cytotoxicity of NK cells, target (721.221 or K562) cells were utilized to perform ^51^Cr release assays as described [2]. Briefly, target cells were incubated with Na_2_^51^CrO_4_ at a dose of 100 μCi per 10^6^ cells at 37°C for 1 h. ^51^Cr-labeled cells were then washed 3 times and suspended in complete R10 medium at a concentration of 10^5^ cells/ml. 10^4^ Cr-labeled target cells were added into each well of V-bottomed 96-well plates, mixed with NK cells at various effector to target cell (E/T) ratios in triplicate, and incubated at 37°C for 4 h. 1% IGEPAL (v/v) (Millipore Sigma, Burlington, CA) was used to lyse all cells to determine the maximal release of ^51^Cr, and ^51^Cr-labeled cells were incubated with media alone to determine the amount spontaneously released without added detergent. Those targets incubated with effector cells were used to determine the experimental release of ^51^Cr. Plates were centrifuged and 100 μl of the supernatants were transferred into a LumaPlate-96 (PerkinElmer, Waltham, MA), dried, and then measured using a TopCount NXT detector (PerkinElmer, Waltham, MA). The percent lysis was calculated as 100 × [(experimental release − spontaneous release) / (maximal release − spontaneous release)]. Target cells were added to each column of the 96-well plate using an 8-channel pipette, and a total count and spontaneous release reading were within each column and used for the experimental release calculation of that column. Technical variation among triplicates (i.e., the SD of the mean of the triplicates) at any given E/T ratio in any assay included for subsequent analysis was less than 20% of the mean of the highest value of the highest E/T ratio. Error bars shown in graphed ^51^Cr release assays are provided for means of biological replicates only (not technical replicates). When measuring NK cell autolysis, NK cells were labeled with ^51^Cr instead of the target cells with the rest of the procedure as described above, and, thus, the measured release of ^51^Cr was that from the NK cells and not target cells.

### RNA interference

For siRNA-mediated perforin knockdown, a mixture of at least 4 siRNAs targeting different ORFs of target genes (Dharmacon siRNA SmartPool, Lafayette, CO) or scrambled siRNA controls (Dharmacon) were introduced into YTS cells using a Nucleofector 2b system and Cell Line Nucleofector Kit R (Lonza Bioscience, Basel, Switzerland). Briefly, 10^6^ YTS cells were mixed with 100 ng siRNA and added into a nucleofection cuvette and electrical pulses applied using program O-017 of the Nucleofector 2b system. After nucleofection, cells were cultured in 1:1 mixture of fresh R10 media and conditioned R10 media. After 48 to 72 h, perforin knockdown in nucleofected cells was validated using flow cytometry (with anti-perforin PE-conjugated clone dG9) and western blot (using anti-perforin clone D48).

For shRNA-mediated perforin knockdown, 4 lentiviral vectors carrying PRF1 gene targeting shRNAs (Dharmacon) were used to package shRNA carrying lentivirus. The vectors were introduced into HEK293T cells using Nucleofector 2b system and Cell Line Nucleofector Kit V (Lonza Bioscience). Briefly, 10^6^ HEK293T cells were mixed with 500 ng plasmid and added into a nucleofection cuvette. Electrical pulses were applied using program Q-001 on the Nucleofector 2b system. After nucleofection, the cells were cultured in fresh DMEM media for 72 h. Virus containing supernatants were collected and filtered using a 0.45-μm filter. Virus concentrations were measured using a p24 ELISA kit (Abcam, Cambridge MA). YTS cells were cultured in a 1:1 mixture of fresh R10 media and viral supernatants for 24 h to allow sufficient infection. After 48 to 72 h, shRNA expression in infected YTS cells were measured based on a coexpressed fluorescent marker (EGFP), and perforin knockdown in shRNA expressing cells was validated using flow cytometry and western blot as with the siRNA experiments.

### Preparation of liposomes and liposome binding assay

DOPC, DPPC, brain SM, and PC (16:0 to 18:1) used in this study were purchased from Avanti Polar Lipids and stored as chloroform solutions (10 mg/ml). To prepare large unilamellar vesicles (LUVs), lipids were mixed as chloroform solutions with intended ratios and were dried under a stream of argon gas and further dried under vacuum for at least 1 h. The resulting lipid films were hydrated in liposome hydration buffer (5 mM HEPES, 150 mM NaCl, 0.1 mM EDTA, adjust pH to 7.4) and subjected to 3 cycles of freezing at −196°C and thawing. To produce a unilamellar liposome population of a uniform size, the LUVs were repeatedly filtered for 25 times through a polycarbonate filter with a pore size of 200 nm (Nuclepore, Marlborough, MA) in a mini-extruder device (Avanti, Alabaster, AL).

For liposome-binding assays, pure protein of interest was incubated with prepared LUV liposomes as described in which the singular protein of interest was assessed for liposome integration [56]. Briefly, 10 μg perforin was incubated with 50 μL of unilamellar liposomes (1 μM) for 20 min at room temperature (RT). After the incubation, the membranes were pelleted by centrifugation at 15,000 × *g* for 30 min at 4°C in a Beckman Optima centrifuge using a SW 50.1 rotor and then washed 3 times with liposome binding buffer (5 mM HEPES, 150 mM NaCl, 20 mM CaCl_2_, adjust pH to 7.4). The liposome-bound perforins were then measured by western blotting using anti-perforin clone D48. Since our liposome-binding assays used a singular protein of interest, like others [56], we utilized the 2 distinct LUV constructs (DOPC/DPPC and PC/SM).

### Isolation of lytic granules from NK cells

Lytic granules were isolated from YTS NK cell lines for lipidomic analyses using the Thermo Fisher Scientific lysosome enrichment kit as previously described [57]. Briefly, 10^8^ YTS cells were harvested from culture and then mechanically lysed using a Dounce homogenizer. The homogenized lysates were centrifuged at 500 × *g* for 10 min to remove the nuclei. Supernatant was collected and carefully overlaid on an 8% to 27% Optiprep gradient and centrifuged at 145,000*g* for 2 h at 4°C in a Beckman Optima centrifuge using a SW 50.1 rotor. After centrifugation, the lysosome band was carefully removed from the Optiprep gradients and washed twice with PBS at 18,000 × *g* for 30 min at 4°C to remove the Optiprep in a Beckman Optima centrifuge using a SW 50.1 rotor. Purified lytic granules were kept in −80°C for future use.

### Lipidomic analysis of lytic granules and whole NK cells

Purified lytic granules and whole YTS cells were flash frozen and submitted to the Biomarkers Core Laboratory at Columbia University Irving Medical Center for targeted lipidomic analysis. A total of 593 individual lipid species were quantified using an LC–MS/MS platform employing an Agilent 6490 Triple Quadrupole (QQQ) MS integrated with an Agilent 1260 Infinity LC system as described.

### Western blot

For western blot analysis, cells or liposomes were removed from solution via centrifugation at 300*g* for 10 min or 10,000 × *g* for 20 min, respectively, supernatants aspirated and then pellets solubilized in 50 to 100 μl of CHAPS cell extract buffer (Cell Signaling Technologies, Danvers, MA) supplemented with Halt protease inhibitor mixture (Thermo Fisher Scientific) and incubated on ice for 20 min. Debris was removed by centrifugation at 20,000 × *g* for 20 min, and supernatants were collected. Polyacrylamide gel electrophoresis, transfer to nitrocellulose membranes, antibody incubation, and detection were performed as described using lysates from 1 × 10^6^ cell equivalents or 0.1 μmol total lipid equivalents of lysosomes [57].

### Flow cytometry–based degranulation and conjugation assays

To measure degranulation in NK cells undergoing 7KC-induced death, a modified previously described approach [2] was used. Briefly, 2 × 10^5^ 7KC-pretreated YTS cells and 2 × 10^5^ 721.221 target cells were coincubated in 200 μl complete RPMI with 10% FCS for 4 h at 37°C in the presence of PI and GolgiStop reagent (BD, Franklin Lakes, NJ). Cell mixtures were then labeled with anti-CD56 (Clone B159; BD Biosciences, Franklin Lakes, NJ) and anti-LAMP-1 (Clone H4A3; BD Biosciences) antibodies at RT for 30 min. After 3 washes with PBS, cells were fixed with 1% PFA in PBS and analyzed using an LSRFortessa flow cytometer (BD) in the Texas Children Hospital. Live and dead NK cell subsets were selected based upon PI signal and each evaluated for the presence of LAMP-1 as a surrogate for degranulation.

To measure conjugation between YTS and K562 cells, a previously described flow cytometry–based assay was used [2]. Briefly, 1 × 10^5^ YTS cells and 2 × 10^5^ K562 cells were coincubated in 200 μl complete R10 for various durations (0, 5, 10, 15, 20, and 30 min) at 37°C. Cell mixtures were then fixed with 2% paraformaldehyde in PBS for 20 min in the dark. After 3 washes, cells were further labeled with anti-CD14 (Clone M5E2; BD Biosciences) and anti-CD56 (Clone B159; BD Biosciences) antibodies for 30 min at RT. Cells were then analyzed using a FACSCelesta flow cytometer in the Stem Cell Core of Columbia Stem Cell Initiative at the Columbia University Irving Medical Center. An acquired cell was considered to be a conjugate between an YTS and K562 cell when it was positive for both CD14 and CD56. The percentage of conjugates was calculated as a feature of conjugated (double positive) YTS cells relative to the total, i.e., those conjugated (double positive) and unconjugated (single positive).

### Image analysis

To measure lipid ordering of presynaptic membranes, lytic granule membranes and membranes at the sites of degranulation the following analyses were conducted using the Fiji version of ImageJ. The lipid ordering of membranes of interest were acquired using 2 separate imaging channels as required by the specific fluorescent packing sensor (S1 Fig). Cell membrane region were distinguished from background using CellMask Red dye to generate binary masks via the Fiji “Threshold” function. Similarly, Lysotracker Red dye was used to generate binary masks of lytic granule via the “Threshold” function. More specifically, images of the 2 fluorescent channels characterizing optimal emission for each lipid sensor were imported to Fiji, and the “image calculator” function was utilized to calculate GP values using the formula shown in S1 Fig. The resulting images were then multiplied with corresponding binary masks to remove background and only preserve the GP values of the membranes of interest. To prepare images for presentation, the default lookup table “Fire” in Fiji was applied, and the scale of display was manually set to “0 to 1” in the “Brightness and Contrast” window. For the enlargements shown in Fig 6A, the scale of display was manually set to “0.2 to 0.8” to visually amplify lipid packing differences. Using resulting images, a quantitative analysis of GP values was obtained using the “Analyze-Measure” function in Fiji, and data were exported to Excel and PRISM for figure plotting and statistical comparisons. To measure and analyze the lipid ordering of lytic granule membranes and the membrane at degranulation sites, images were processed using the same procedure described above but were only quantified inside of the determined regions of interest (ROIs; see below). For time-lapse imaging series, this procedure was applied to each frame individually.

To determine the segregation of lytic granules and degranulation sites from background, ROIs were generated from images of Lysotracker Red dye and pHluorin, respectively, using “Analyze Particles” function in Fiji. The images of Lysotracker Red dye and pHluorin were thresholded using the default function in Fiji to generate binary masks. Then, the “Analyze Particles” function was applied to automatically define ROIs around masks of granules or pHluorin patches. The pHluorin patches at the membrane were used to define sites of degranulation as described previously [39]. The parameter settings used for the measurement of granules or pHluorin patches were “*Size*: *0*.*5-Infinity*, *Circularity*: *0–1” and “Size*: *0-Infinity*, *Circularity*: *0–1*”, respectively. The borders of the identified ROIs were saved and overlaid on top of the original image to highlight granules or degranulation sites, which were used in the images presented. GP values and pHluorin intensities were measured inside of ROIs using the Fiji “Analyze-Measure” function, and data were exported to Excel and PRISM for figure plotting and statistical comparison. For time-lapse imaging series, this procedure was applied to each frame individually.

Data deposited in the Dryad repository: https://doi.org/10.5061/dryad.hqbzkh1f4 [58].

## Supporting information

S1 Fig Fluorescence emission spectra and GP value calculation of Laurdan and Di-4-ANEPPDHQ in liposomes. Fluorescence emission spectra of Laurdan (A) and Di-4-ANEPPDHQ (Di-4) (B) in ordered (blue) and disordered (red) liposome membranes were measured. Representative results from 10 distinct liposomes were plotted to demonstrate shifts in their emission wavelength indicating different membrane packing densities. For quantitative analysis, the GP value of Laurdan or Di-4 stained membranes were measured via microscopy in 2 separate fluorescent channels and calculated using the formula shown in (C) and (D), respectively. GP, generalized polarization.
(TIF)

S2 Fig Membrane packing distribution in conjugated and unconjugated YTS cells and NK92 cells. The lipid packing of NK cell membranes in fluorescent packing sensor (Laurdan, top; Di-4, bottom) labeled resting YTS (A) and NK92 (B) cells were measured using confocal microscopy. Representative images (of 15 evaluations) were overlaid with a pseudocolor scale to visually demonstrate the distribution of membrane packing densities. In each pair of images, the left shows a DIC image and the right the pseudocolor florescence. Images include both conjugated and unconjugated cells for comparison. Scale bar: 10 μm. DIC, differential interference contrast; GP, generalized polarization; NK, natural killer.
(TIF)

S3 Fig Cytotoxicity and survival of ex vivo human NK cells. Cytotoxic function (A) and survival (B) of ex vivo human NK cells were measured after their labeling with ^51^Cr and when incubated with K562 cells with (red) or without (blue) 7KC pretreatment. Primary human NK cells were isolated from 5 distinct unrelated healthy donors (eNK cells) and used in these assays without additional propagation in vitro. The outcome of each assay across multiple NK cell to target cell ratios was plotted separately. All values presented represent averages of 3 replicated wells (i.e., technical repeats), and error bars display ± SD of these technical replicates. eNK, ex vivo NK; NK, natural killer; 7KC, 7-ketocholesterol.
(TIF)

S4 Fig Alternative approach to the use and specificity of 7KC in inducing NK lysis. YTS cells were pretreated with Nystatin in the specified concentrations or 30 ug/ml 7KC. The cytotoxic function of these YTS cells against ^51^Cr-labeled K562 cells (A, left) or survival of these YTS cells that had been ^51^Cr labeled and incubated with unlabeled K562 cells (A, right) was measured in a 4-h assay. (B) Survival of YTS cells after a 4-h incubation in the absence of target cells over a range of 7KC concentrations was measured based on their release of calcein green dye to evaluate any direct toxicity of 7KC (or MbCD vehicle) measured using a fluorescence cell counter. (C) YTS cells were pretreated with PMA (25 ng/ml) and ionomycin (250 ng/ml) to induce their activation and then evaluated for survival based on their release of calcein green dye after a 4-h incubation in the absence of target cells over a range of 7KC concentrations. In all cases, experiments are representative of 3 independent repeats, and error bars show ± SD of technical replicates of an individual experiment. NK, natural killer; 7KC, 7-ketocholesterol.
(TIF)

S5 Fig YTS cells conjugate with K562 cells. YTS cells were coincubated with K562 target cells for 0–30 min at 37°C and then stained with anti-CD14 and anti-CD56 antibodies prior to analysis via flow cytometry to measure conjugation as determined by double positivity (A). The gated region in each plot was selected to correspond to those dually positive for CD14 and CD56, and the percentage of total YTS cells in conjugates at each time was graphed (B) to demonstrate change with increasing effector–target cell incubation time. The data shown are from a single experiment but representative of 2 independent experiments.
(TIF)

S6 Fig Chemical depletion of perforin in NK cells by CMA. NK92 cells were treated with 100 nM CMA in media at 37°C for 1 h, fixed and permeabilized, and then stained with PE-conjugated anti-perforin antibody clone D48 (which recognizes total perforin as opposed to clones, which recognize more mature forms). Then, intracellular level of perforin was measured by flow cytometry. Data (left) results from 3 independent experiments from which mean values ± SD are shown (right, *p* < 0.0001, two-tailed *t* test). CMA, Concanamycin A; NK, natural killer; PE, phycoerythrin.
(TIF)

S7 Fig Validation of perforin knockdown. Perforin levels in scramble control siRNA- or PRF1 siRNA-transfected YTS cells were measured by western blot (A) using anti-perforin antibody (Clone D48) for detecting perforin, which was quantified using densitometry across independently repeated experiments using different cell preparations (B) in which means ± SD are shown (difference between means *p* < 0.0001 via Student *t* test). Levels of perforin were also measured using fixed cell confocal imaging (C) with anti-perforin antibody (clone δG9) for detecting perforin. The perforin fluorescence in individual cells was quantified and plotted (D) with the mean ± SD depicted (difference between means *p* < 0.0001 via Student *t* test). Perforin levels in shRNA-transfected and untransfected YTS cells were measured and quantified using fixed cell imaging (E and F) as they were for the siRNA. They were additionally evaluated for perforin levels by flow cytometry (G) after staining with PE-conjugated anti-perforin antibody (clone δG9) where the parental YTS cells are represented in pink, and each of the other histograms represents a different shRNA-transfected YTS cell culture (representative of 3 independent experiments of shRNA). Scale bar: 10 μm. MFI, mean fluorescence intensity; PE, phycoerythrin; shRNA, short hairpin RNA; siRNA, small interfering RNA.
(TIF)

S8 Fig Validation of liposome membrane packing. Fluorescence emission spectra of Di-4-ANEPPDHQ in different DOPC:DPPC liposomes (A) were measured. Results representative of 3 independent experiments were plotted as histograms to demonstrate the shifts of their emission wavelength for different membrane packing densities. (B) GP values of DOPC:DPPC liposome membranes were measured with Di-4-ANEPPDHQ by dual channel microscopy from 3 individual experiments and the mean ± SD plotted. (C) Fluorescence emission spectra of Di-4-ANEPPDHQ in different PC:SM:CHO liposomes are shown for a representative experiment of 3 and (D) the mean GP values of PC:SM:CHO liposome membranes from the independent experiments +SD graphed. CHO, cholesterol; DOPC, dioleoyl phosphatidylcholine; DPPC, dipalmitoyl phosphatidylcholine; GP, generalized polarization; PC, phosphatidylcholine; SM, sphingomyelin.
(TIF)

S9 Fig Validation of LAMP-1 knockdown by siRNA. (A) Evaluation of siRNA-mediated knockdown of LAMP-1 in YTS cells from whole cell lysates of untransfected (Lane 1), scrambled control siRNA-transfected YTS cells (Lane 2), or LAMP-1 siRNA-transfected YTS cells (Lane 3) by western blot analysis. A representative blot (left) using the lysate from 2 × 10^5^ cells per lane was quantified via densitometry for LAMP1 and β-actin signal and plotted (right) with the former normalized to the latter. (B) Evaluation of siRNA-mediated knockdown of LAMP-1 in lytic granule regions of YTS cells by quantitative confocal imaging. Representative images of either control siRNA- (left) or LAMP-1 siRNA-treated (right) YTS cells costained with LAMP-1 (red), β-actin (yellow), and perforin (green) demonstrate an overall reduction of LAMP-1 in the LAMP-1 siRNA-treated cells. Notably, there is also reduction of LAMP-1 in the regions of perforin, which would correspond to the lytic granules since here peforin was stained using antibody clone δG9 recognizing mature perforin. (Right) Measurements of the LAMP-1 fluorescence intensity were taken in the regions of perforin fluorescence (lytic granules) within an individual cell and normalized to the fluorescence intensity of β-actin in that cell. Quantifications from cells derivative from 3 biological repeats were pooled and plotted together (*n =* 79 for scramble siRNA and *n* = 84 for LAMP-1 siRNA-transfected YTS). An unpaired *t* test was used for the comparison of the means, and the difference was significant *p* < 0.0001. Scale bar: 10 μm. siRNA, small interfering RNA.
(TIF)

S1 Table Lipid composition of whole YTS cell membranes and isolated YTS lytic granule membranes. (DOCX)

S1 Raw Images (PDF)

## Abbreviations

APC: antigen-presenting cell
CMA: Concanamycin A
CTL: cytotoxic T lymphocyte
DOPC: dioleoyl phosphatidylcholine
DPPC: dipalmitoyl phosphatidylcholine
E/T: effector to target cell
GM3: monosialodihexosylganglioside
GP: generalized polarization
LUV: large unilamellar vesicle
MTOC: microtubule organizing center
NK: natural killer
PC: phosphatidylcholine
PI: propidium iodide
ROI: region of interest
RT: room temperature
shRNA: short hairpin RNA
siRNA: small interfering RNA
SM: sphingomyelin
TIRF: total internal reflection fluorescent
7KC: 7-ketocholesterol
