# Supplementary figures and images for: Degranulation enhances presynaptic membrane packing, which protects NK cells from perforin-mediated autolysis
Source: PLoS Biol. 2021 Aug 3;19(8):e3001328. doi: 10.1371/journal.pbio.3001328 (PMC8330931; doi:10.1371/journal.pbio.3001328)

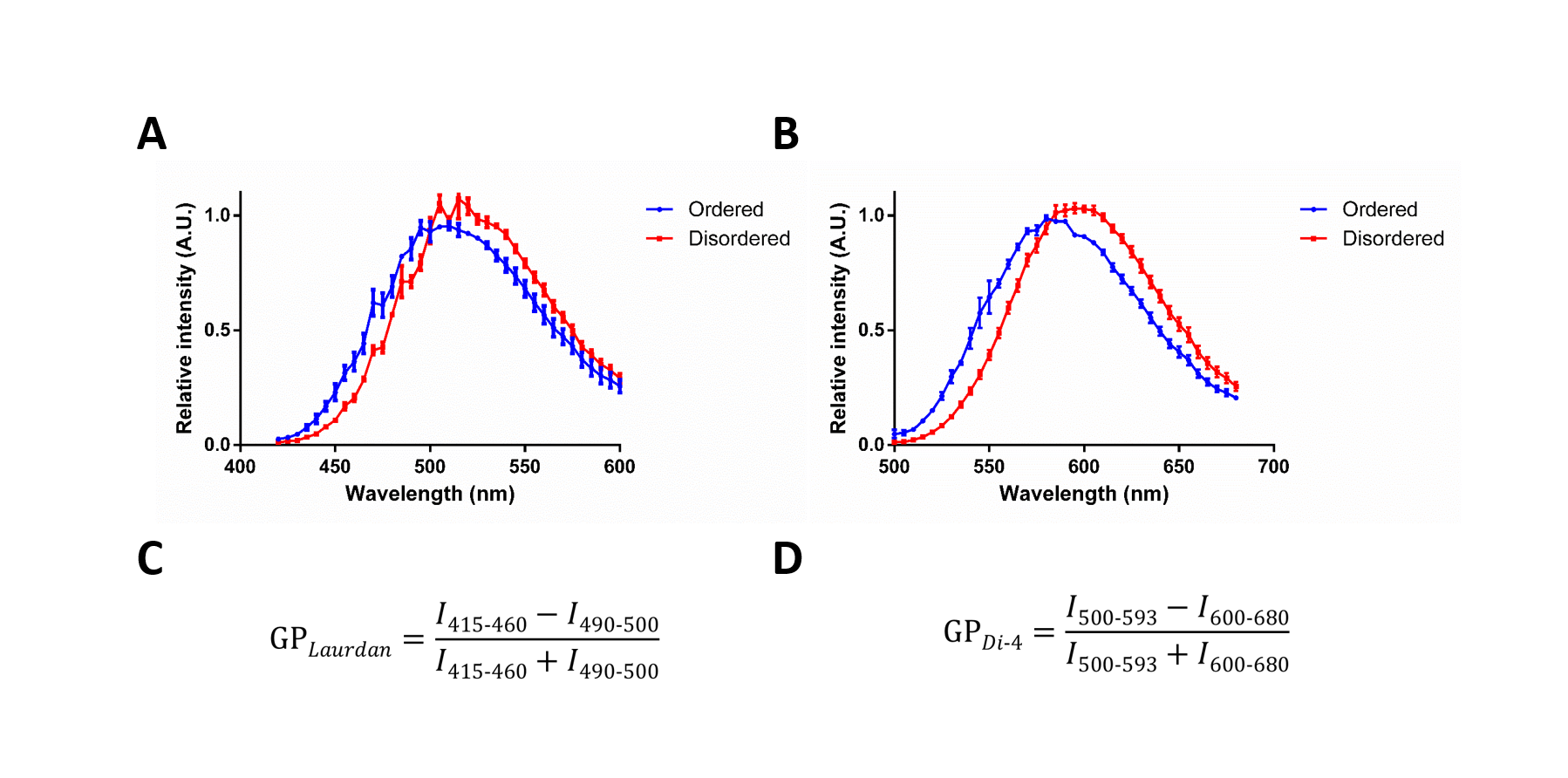

Supplement: S1 Fig — Fluorescence emission spectra of Laurdan (A) and Di-4-ANEPPDHQ (Di-4) (B) in ordered (blue) and disordered (red) liposome membranes were measured. Representative results from 10 distinct liposomes were plotted to demonstrate shifts in their emission wavelength indicating different membrane packing densities. For quantitative analysis, the GP value of Laurdan or Di-4 stained membranes were measured via microscopy in 2 separate fluorescent channels and calculated using the formula shown in (C) and (D), respectively. GP, generalized polarization. (TIF) [file pbio.3001328.s001.tif]

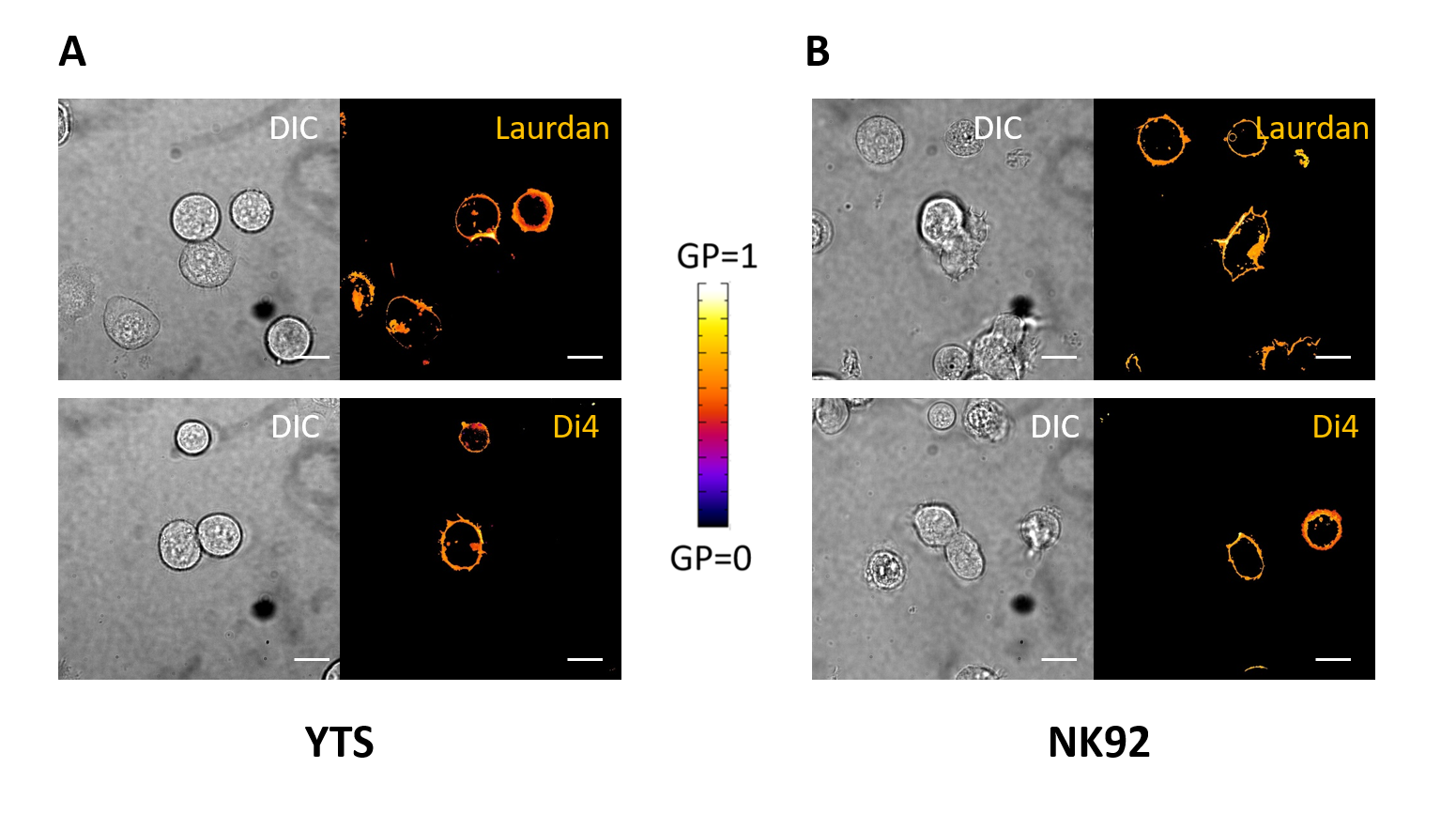

Supplement: S2 Fig — The lipid packing of NK cell membranes in fluorescent packing sensor (Laurdan, top; Di-4, bottom) labeled resting YTS (A) and NK92 (B) cells were measured using confocal microscopy. Representative images (of 15 evaluations) were overlaid with a pseudocolor scale to visually demonstrate the distribution of membrane packing densities. In each pair of images, the left shows a DIC image and the right the pseudocolor florescence. Images include both conjugated and unconjugated cells for comparison. Scale bar: 10 μm. DIC, differential interference contrast; GP, generalized polarization; NK, natural killer. (TIF) [file pbio.3001328.s002.tif]

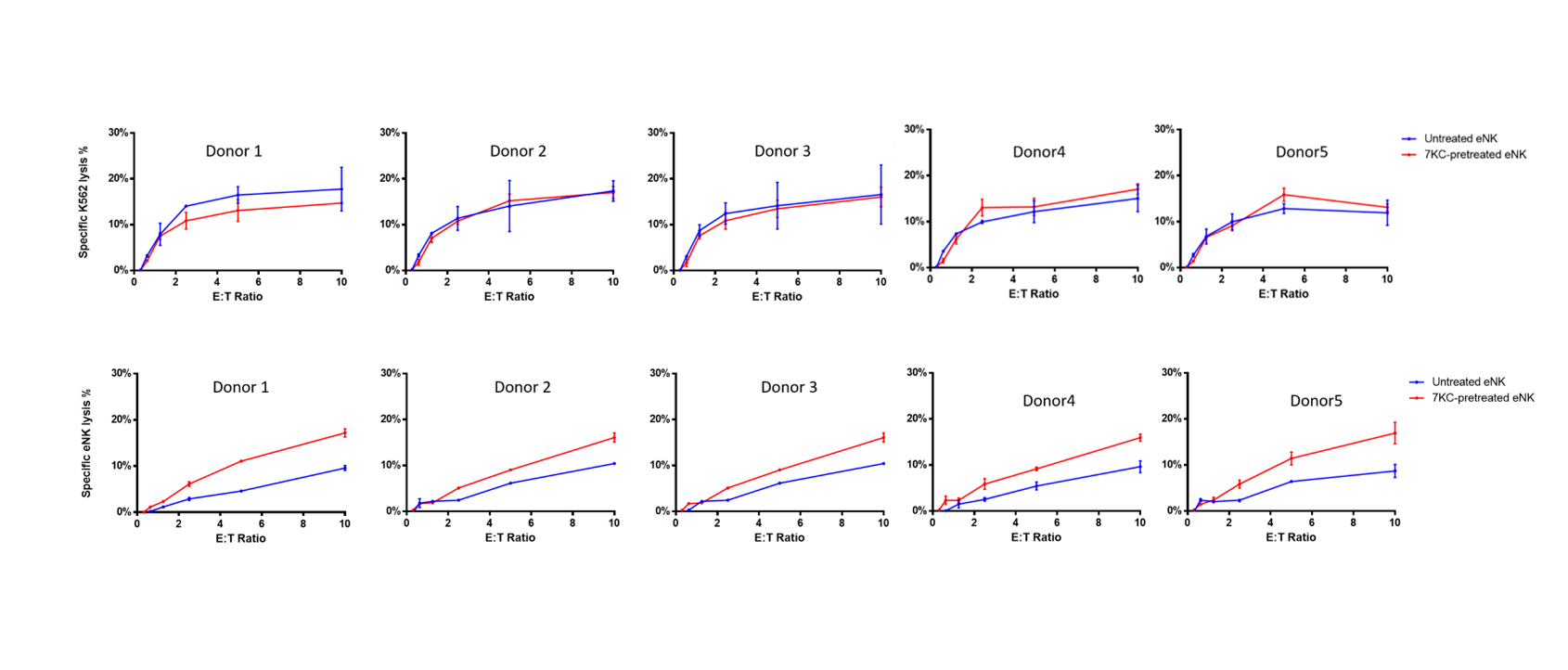

Supplement: S3 Fig — Cytotoxic function (A) and survival (B) of ex vivo human NK cells were measured after their labeling with 51Cr and when incubated with K562 cells with (red) or without (blue) 7KC pretreatment. Primary human NK cells were isolated from 5 distinct unrelated healthy donors (eNK cells) and used in these assays without additional propagation in vitro. The outcome of each assay across multiple NK cell to target cell ratios was plotted separately. All values presented represent averages of 3 replicated wells (i.e., technical repeats), and error bars display ± SD of these technical replicates. eNK, ex vivo NK; NK, natural killer; 7KC, 7-ketocholesterol. (TIF) [file pbio.3001328.s003.tif]

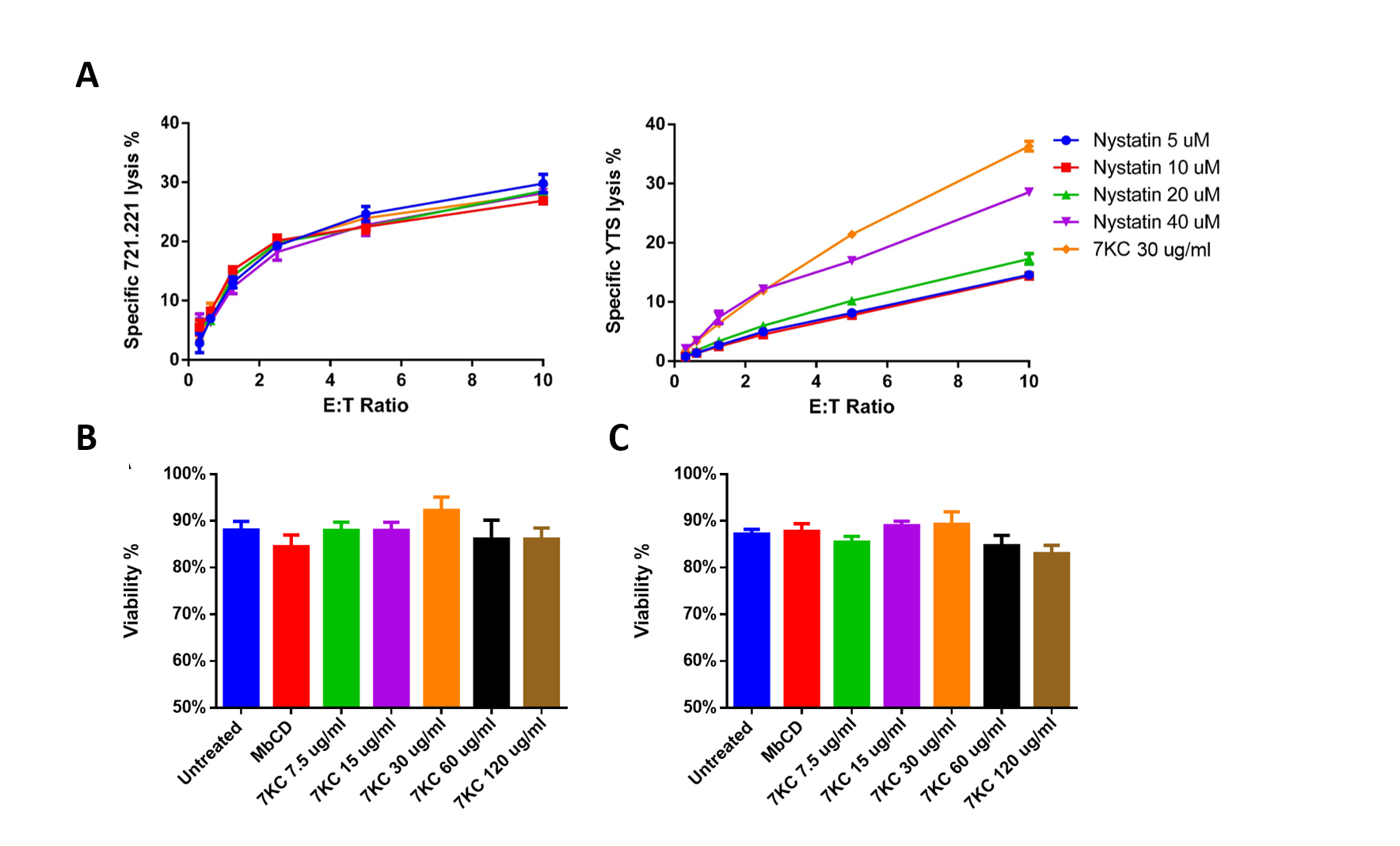

Supplement: S4 Fig — YTS cells were pretreated with Nystatin in the specified concentrations or 30 ug/ml 7KC. The cytotoxic function of these YTS cells against 51Cr-labeled K562 cells (A, left) or survival of these YTS cells that had been 51Cr labeled and incubated with unlabeled K562 cells (A, right) was measured in a 4-h assay. (B) Survival of YTS cells after a 4-h incubation in the absence of target cells over a range of 7KC concentrations was measured based on their release of calcein green dye to evaluate any direct toxicity of 7KC (or MbCD vehicle) measured using a fluorescence cell counter. (C) YTS cells were pretreated with PMA (25 ng/ml) and ionomycin (250 ng/ml) to induce their activation and then evaluated for survival based on their release of calcein green dye after a 4-h incubation in the absence of target cells over a range of 7KC concentrations. In all cases, experiments are representative of 3 independent repeats, and error bars show ± SD of technical replicates of an individual experiment. NK, natural killer; 7KC, 7-ketocholesterol. (TIF) [file pbio.3001328.s004.tif]

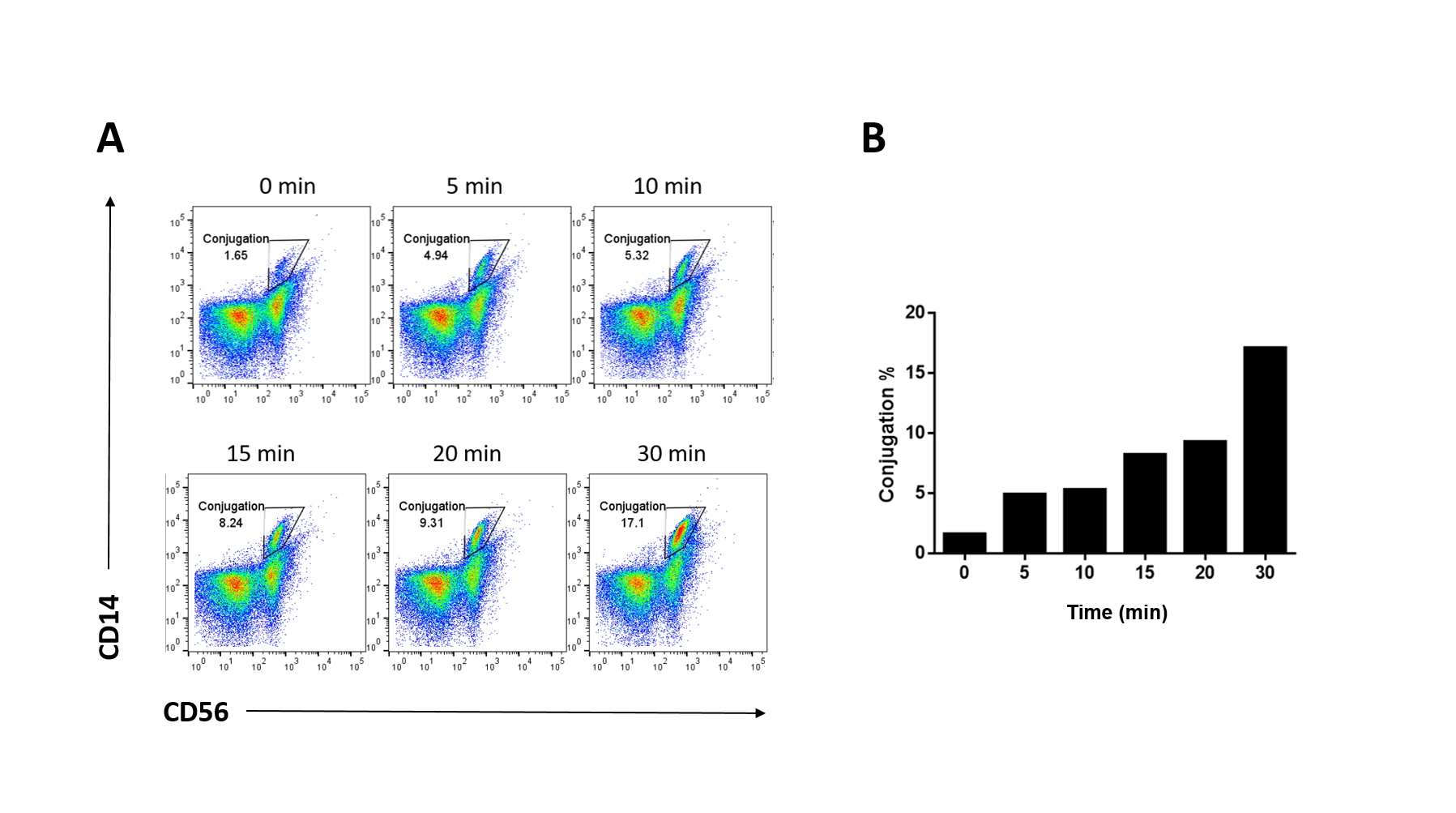

Supplement: S5 Fig — YTS cells were coincubated with K562 target cells for 0–30 min at 37°C and then stained with anti-CD14 and anti-CD56 antibodies prior to analysis via flow cytometry to measure conjugation as determined by double positivity (A). The gated region in each plot was selected to correspond to those dually positive for CD14 and CD56, and the percentage of total YTS cells in conjugates at each time was graphed (B) to demonstrate change with increasing effector–target cell incubation time. The data shown are from a single experiment but representative of 2 independent experiments. (TIF) [file pbio.3001328.s005.tif]

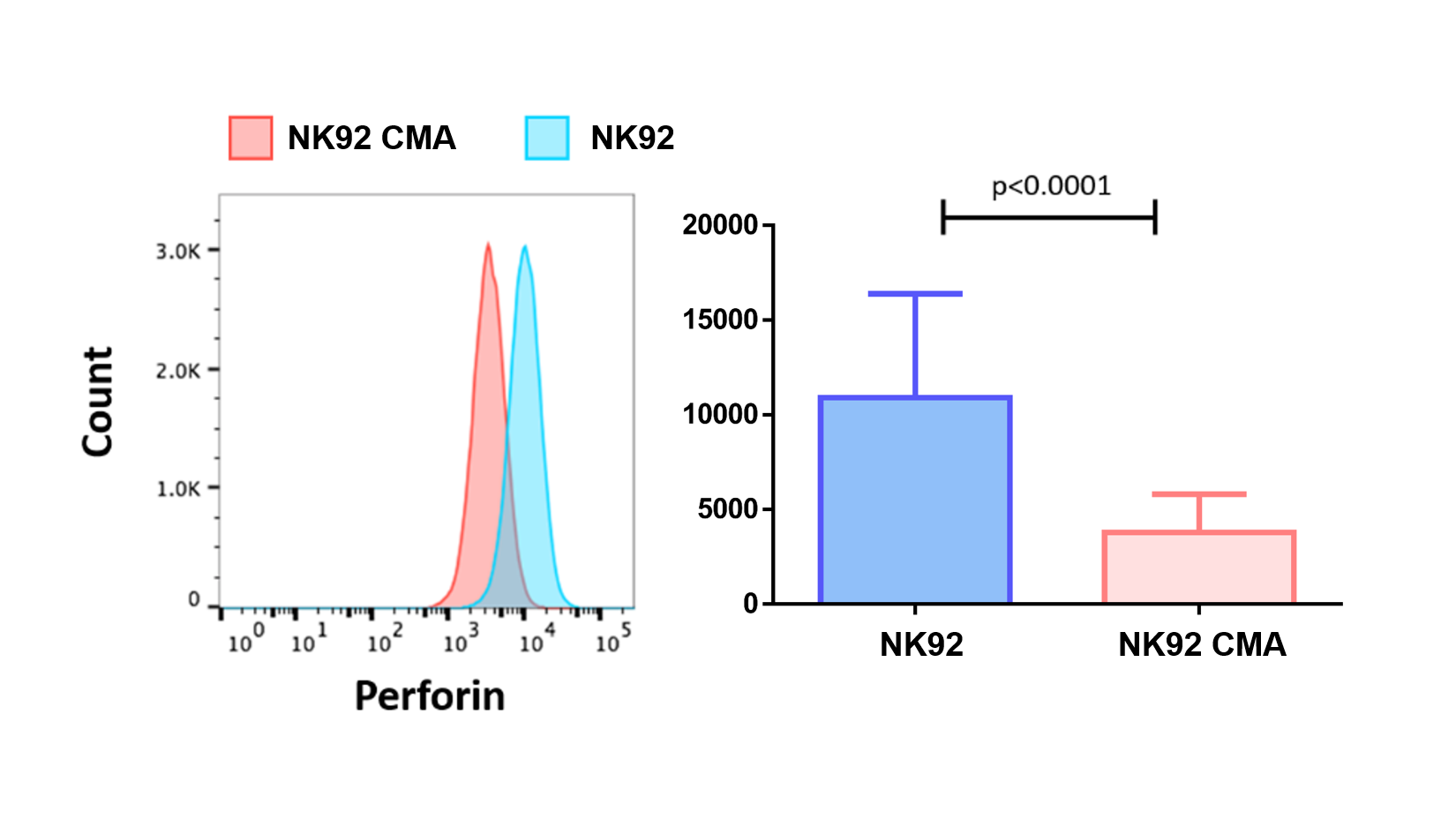

Supplement: S6 Fig — NK92 cells were treated with 100 nM CMA in media at 37°C for 1 h, fixed and permeabilized, and then stained with PE-conjugated anti-perforin antibody clone D48 (which recognizes total perforin as opposed to clones, which recognize more mature forms). Then, intracellular level of perforin was measured by flow cytometry. Data (left) results from 3 independent experiments from which mean values ± SD are shown (right, p < 0.0001, two-tailed t test). CMA, Concanamycin A; NK, natural killer; PE, phycoerythrin. (TIF) [file pbio.3001328.s006.tif]

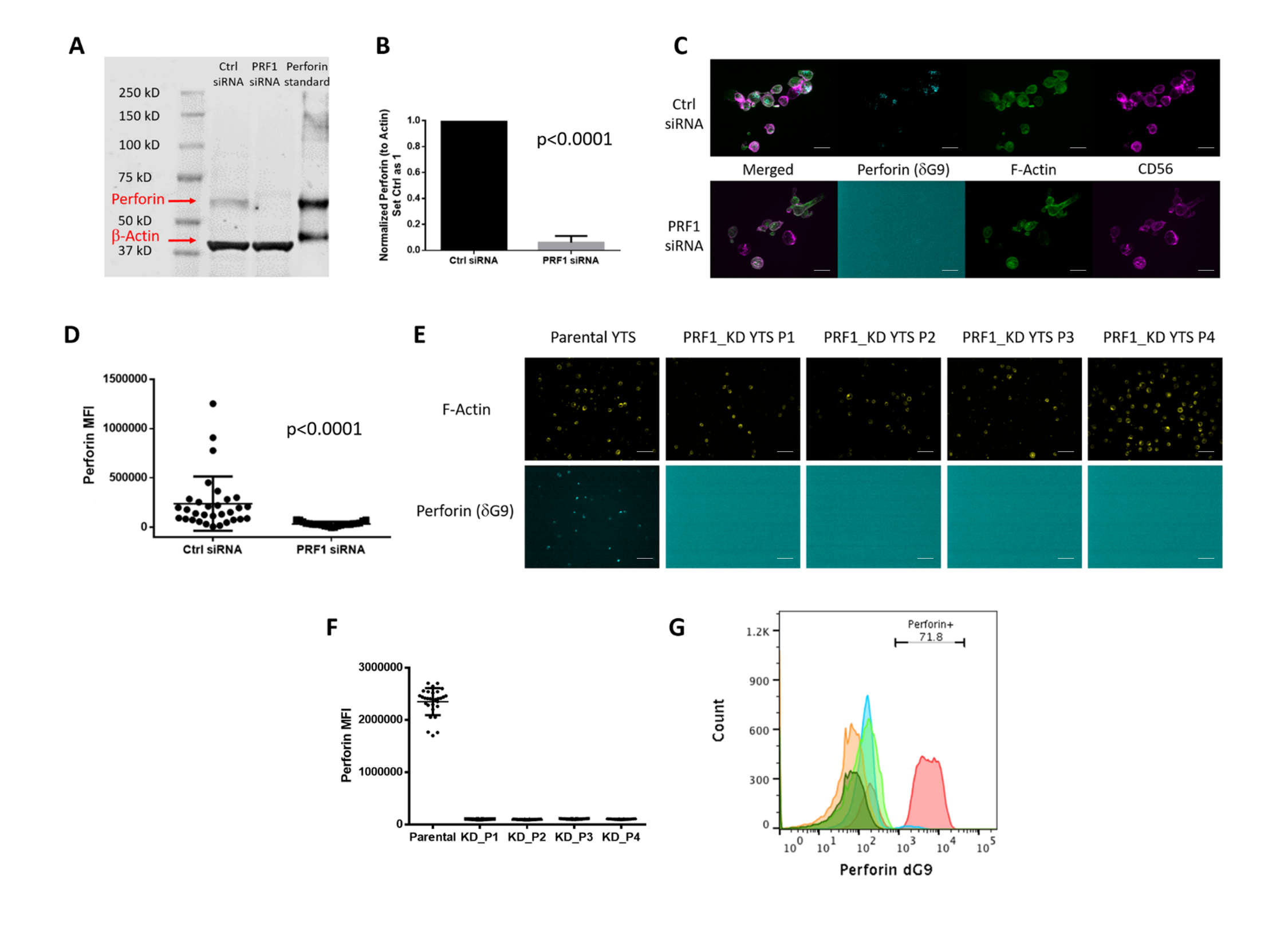

Supplement: S7 Fig — Perforin levels in scramble control siRNA- or PRF1 siRNA-transfected YTS cells were measured by western blot (A) using anti-perforin antibody (Clone D48) for detecting perforin, which was quantified using densitometry across independently repeated experiments using different cell preparations (B) in which means ± SD are shown (difference between means p < 0.0001 via Student t test). Levels of perforin were also measured using fixed cell confocal imaging (C) with anti-perforin antibody (clone δG9) for detecting perforin. The perforin fluorescence in individual cells was quantified and plotted (D) with the mean ± SD depicted (difference between means p < 0.0001 via Student t test). Perforin levels in shRNA-transfected and untransfected YTS cells were measured and quantified using fixed cell imaging (E and F) as they were for the siRNA. They were additionally evaluated for perforin levels by flow cytometry (G) after staining with PE-conjugated anti-perforin antibody (clone δG9) where the parental YTS cells are represented in pink, and each of the other histograms represents a different shRNA-transfected YTS cell culture (representative of 3 independent experiments of shRNA). Scale bar: 10 μm. MFI, mean fluorescence intensity; PE, phycoerythrin; shRNA, short hairpin RNA; siRNA, small interfering RNA. (TIF) [file pbio.3001328.s007.tif]

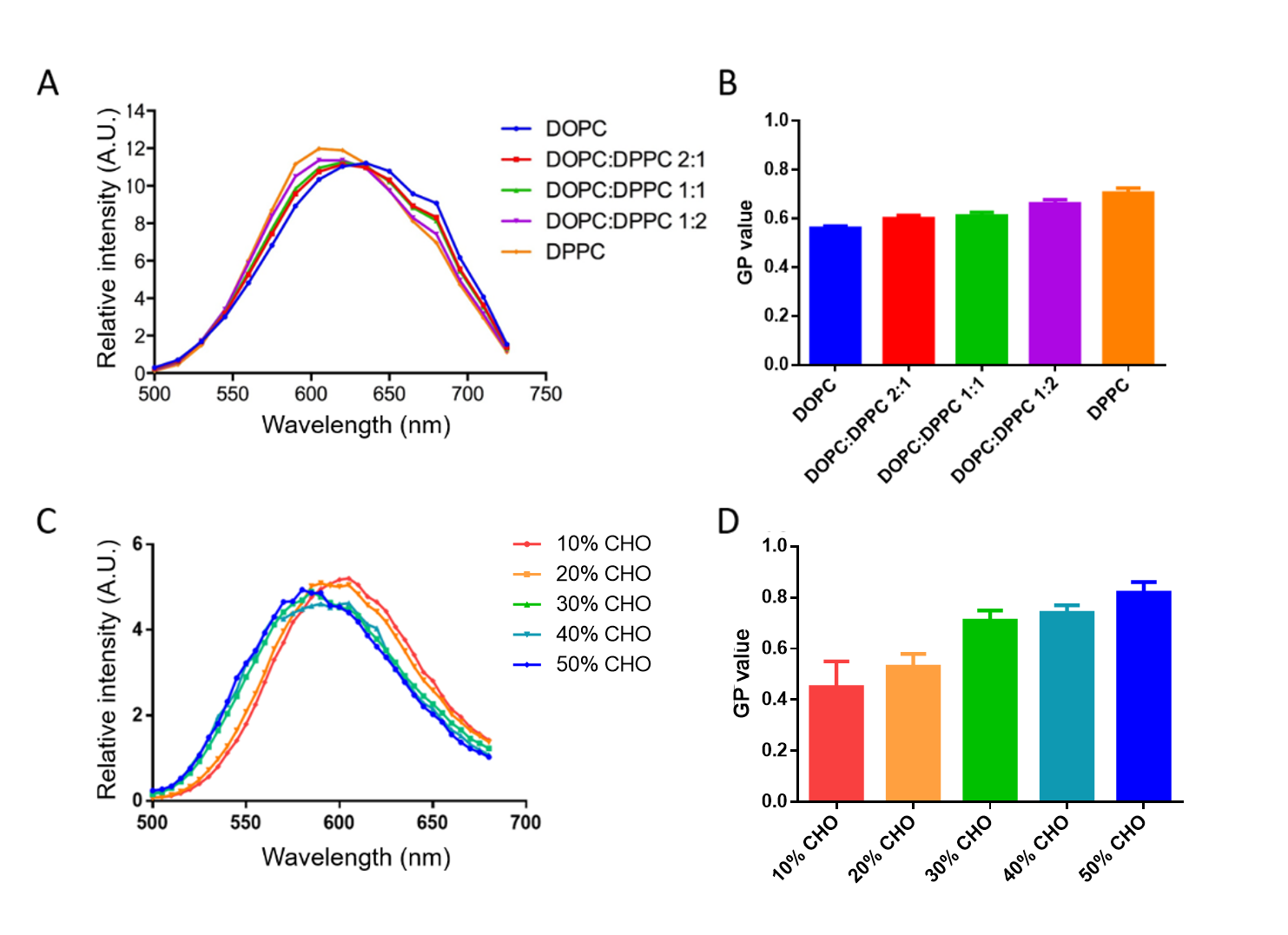

Supplement: S8 Fig — Fluorescence emission spectra of Di-4-ANEPPDHQ in different DOPC:DPPC liposomes (A) were measured. Results representative of 3 independent experiments were plotted as histograms to demonstrate the shifts of their emission wavelength for different membrane packing densities. (B) GP values of DOPC:DPPC liposome membranes were measured with Di-4-ANEPPDHQ by dual channel microscopy from 3 individual experiments and the mean ± SD plotted. (C) Fluorescence emission spectra of Di-4-ANEPPDHQ in different PC:SM:CHO liposomes are shown for a representative experiment of 3 and (D) the mean GP values of PC:SM:CHO liposome membranes from the independent experiments +SD graphed. CHO, cholesterol; DOPC, dioleoyl phosphatidylcholine; DPPC, dipalmitoyl phosphatidylcholine; GP, generalized polarization; PC, phosphatidylcholine; SM, sphingomyelin. (TIF) [file pbio.3001328.s008.tif]

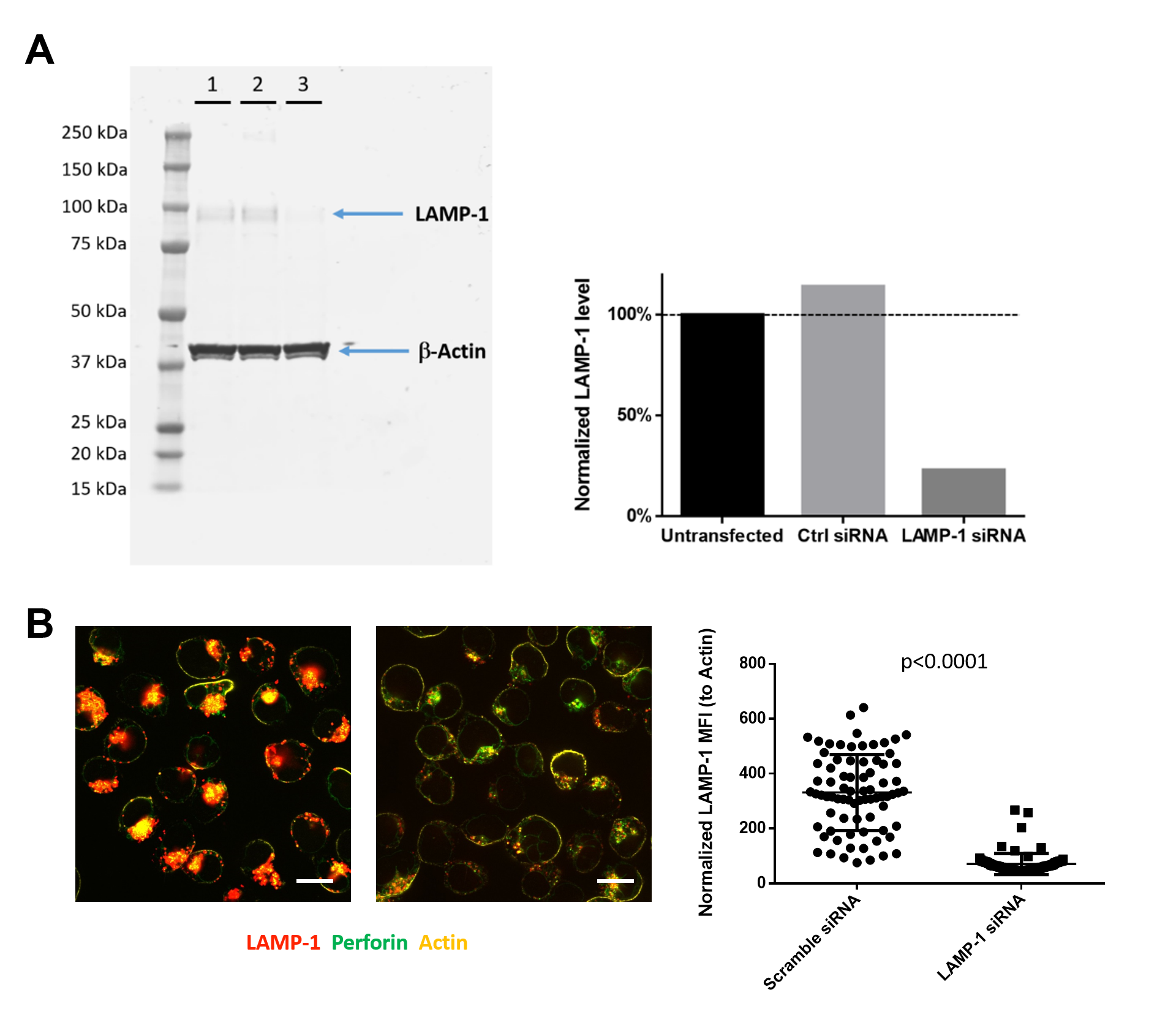

Supplement: S9 Fig — (A) Evaluation of siRNA-mediated knockdown of LAMP-1 in YTS cells from whole cell lysates of untransfected (Lane 1), scrambled control siRNA-transfected YTS cells (Lane 2), or LAMP-1 siRNA-transfected YTS cells (Lane 3) by western blot analysis. A representative blot (left) using the lysate from 2 × 10^5^ cells per lane was quantified via densitometry for LAMP1 and β-actin signal and plotted (right) with the former normalized to the latter. (B) Evaluation of siRNA-mediated knockdown of LAMP-1 in lytic granule regions of YTS cells by quantitative confocal imaging. Representative images of either control siRNA- (left) or LAMP-1 siRNA-treated (right) YTS cells costained with LAMP-1 (red), β-actin (yellow), and perforin (green) demonstrate an overall reduction of LAMP-1 in the LAMP-1 siRNA-treated cells. Notably, there is also reduction of LAMP-1 in the regions of perforin, which would correspond to the lytic granules since here peforin was stained using antibody clone δG9 recognizing mature perforin. (Right) Measurements of the LAMP-1 fluorescence intensity were taken in the regions of perforin fluorescence (lytic granules) within an individual cell and normalized to the fluorescence intensity of β-actin in that cell. Quantifications from cells derivative from 3 biological repeats were pooled and plotted together (n = 79 for scramble siRNA and n = 84 for LAMP-1 siRNA-transfected YTS). An unpaired t test was used for the comparison of the means, and the difference was significant p < 0.0001. Scale bar: 10 μm. siRNA, small interfering RNA. (TIF) [file pbio.3001328.s009.tif]
