# Supplementary material for: Degranulation enhances presynaptic membrane packing, which protects NK cells from perforin-mediated autolysis
Source: PLoS Biol. 2021 Aug 3;19(8):e3001328. doi: 10.1371/journal.pbio.3001328 (PMC8330931; doi:10.1371/journal.pbio.3001328)
Supplement: S1 Table — (DOCX) [file pbio.3001328.s010.docx]

**Table. S1 Lipid composition of whole YTS cell membranes and isolated**

**YTS lytic granule membranes.**

|  | Independent repeat 1 | | Independent repeat 2 | |
| --- | --- | --- | --- | --- |
| Lipids | Whole Cell (Mol%) | Granule (Mol%) | Whole Cell (Mol%) | Granule (Mol%) |
| FC | 32.2 | 7.908 | 34.6 | 8.67 |
| CE | 5.56 | 0.409 | 6.01 | 0.42 |
| AC | 0.098 | 0.057 | 0.1 | 0.06 |
| MG | 0.193 | 24.61 | 0.2 | 26.06 |
| DG | 0.812 | 2.879 | 0.84 | 3.18 |
| TG | 1.038 | 0.371 | 1.12 | 0.39 |
| Cer | 0.559 | 0.775 | 0.56 | 0.85 |
| dhCer | 0.019 | 0.087 | 0.02 | 0.09 |
| SM | 5.561 | 13.61 | 5.73 | 15.08 |
| dhSM | 3.789 | 12.45 | 4.14 | 12.86 |
| MhCer | 0.137 | 0.486 | 0.14 | 0.52 |
| Sulf | 0.009 | 0.019 | 0.01 | 0.02 |
| LacCer | 0.029 | 0.036 | 0.03 | 0.04 |
| GM3 | 0.065 | 1.185 | 0.07 | 1.22 |
| GB3 | 0 | 0 | 0 | 0 |
| PA | 0.188 | 0.379 | 0.19 | 0.41 |
| PC | 8.513 | 7.128 | 8.86 | 7.91 |
| PCe | 3.461 | 7.889 | 3.71 | 7.97 |
| PE | 11.52 | 1.779 | 12.62 | 1.83 |
| PEp | 11.27 | 1.514 | 11.9 | 1.55 |
| PS | 2.95 | 1.685 | 2.99 | 1.84 |
| PI | 4.842 | 4.594 | 4.95 | 4.71 |
| PG | 0.078 | 0.046 | 0.08 | 0.05 |
| BMP | 0.326 | 0.316 | 0.36 | 0.32 |
| AcylPG | 0 | 0.009 | 0 | 0.01 |
| LPC | 0.285 | 2.034 | 0.3 | 2.23 |
| LPCe | 0.019 | 0.292 | 0.02 | 0.31 |
| LPE | 0.174 | 0.529 | 0.19 | 0.58 |
| LPEp | 0.122 | 0.019 | 0.13 | 0.02 |
| LPI | 0.055 | 0.21 | 0.06 | 0.22 |
| LPS | 0.055 | 0.566 | 0.06 | 0.58 |
| NAPE | 0 | 0 | 0 | 0 |
| NAPS | 0 | 0 | 0 | 0 |
| NSer | 0 | 0.018 | 0 | 0.02 |

Lipid composition of whole YTS cell membranes and isolated YTS lytic granule membranes were determined by LC-MS in 2 independent experiments. Lipid levels are expressed as average Mol% of the total sum of moles of lipids detected. The nomenclature abbreviations are: FC: free cholesterol, PE: phosphatidylethanolamines, CE: cholesteryl ester, SM: sphingomyelin, dhSM: dihyodrosphingomyelin, MG: monoglycerols, PEp: phosphatidylethanolamine-based plasmalogens, PC: phosphatidylcholine, PI: phosphatidylinositols, PCe: alkylacyl phosphatidylcholine, PS: phosphatidylserines, Cer: ceramides, AC: acyl carnitines, DG: diglycerides, TG: triglycerides, dhCer:  dihydroceramide, MhCer: monohexosylceramide, Sulf: sulfatide, LacCer: lactosylceramide, GM3: monosialodihexosylganglioside, GB3: globotriaosylceramide, PA: phosphatidic acids, PI: phosphatidylinositols, PG: phosphatidylgylcerols, BMP: bis[monoacylglycero]phosphate, AcylPG: acylphosphatidyl glycerol, LPC: lysophosphatidylcholine, LPCe: ether lysophosphatidylcholine, LPE: lysophosphatidylethanolamine, LPEp: plasmalogen lysophosphatidylethanolamine, LPI: lysophosphatidylinositol, NAPE: N-acyl phosphatidylethanolamine, NAPS: N-acyl phosphatidylserine, Nser: N-Acyl Serine.
