# Supplementary material for: Degranulation enhances presynaptic membrane packing, which protects NK cells from perforin-mediated autolysis
Source: PLoS Biol. 2021 Aug 3;19(8):e3001328. doi: 10.1371/journal.pbio.3001328 (PMC8330931; doi:10.1371/journal.pbio.3001328)
Supplement: S1 Raw Images — (PDF) [file pbio.3001328.s011.pdf]

# Raw pictures for Figure 5A

Three raw gel images used in Figure 5A are attached below:

Proteins in each samples: perforin only

Capture method: Odyssey CLx scanner

Molecular weight markers and loading order have been annotated without obscuring any data or background bands.

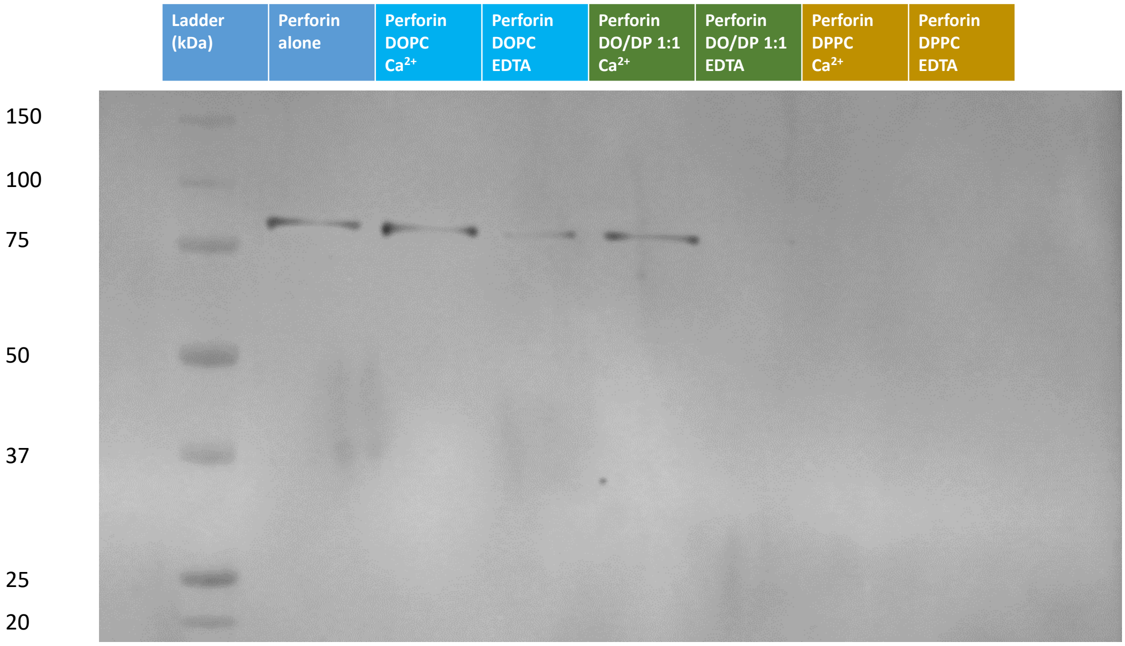

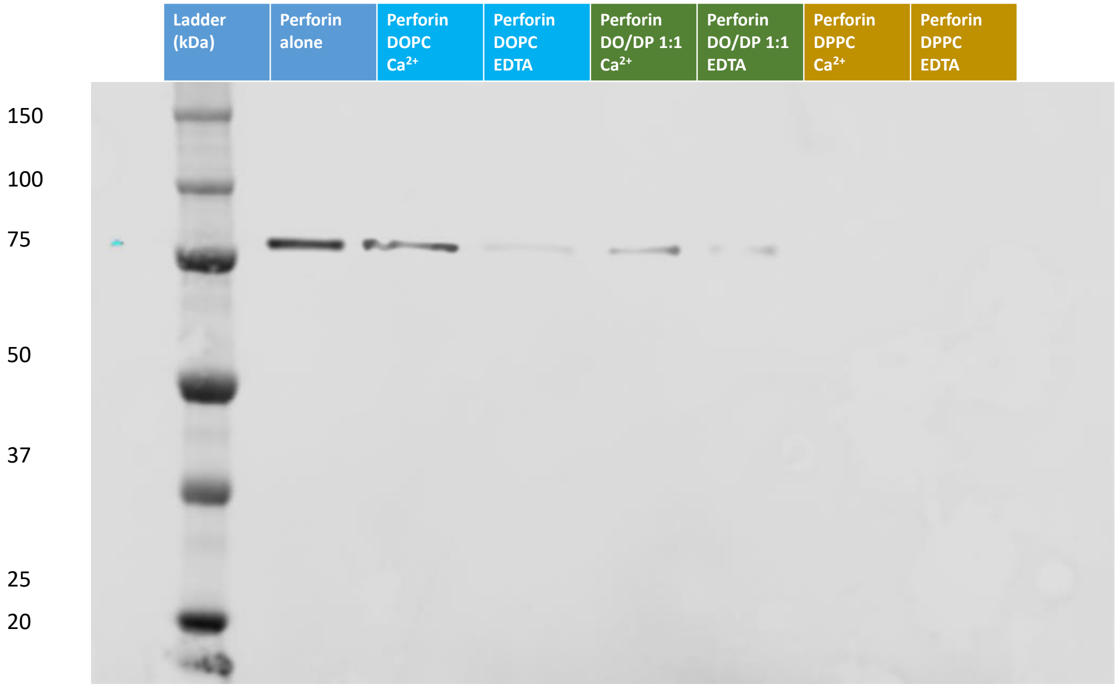
Ladder (kDa)
Perforin alone
Perforin DOPC Ca2+
Perforin DOPC EDTA
Perforin DO/DP 1:1 Ca2+
Perforin DO/DP 1:1 EDTA
Perforin DPPC Ca2+
Perforin DPPC EDTA
150
100
75
50
37
25
20

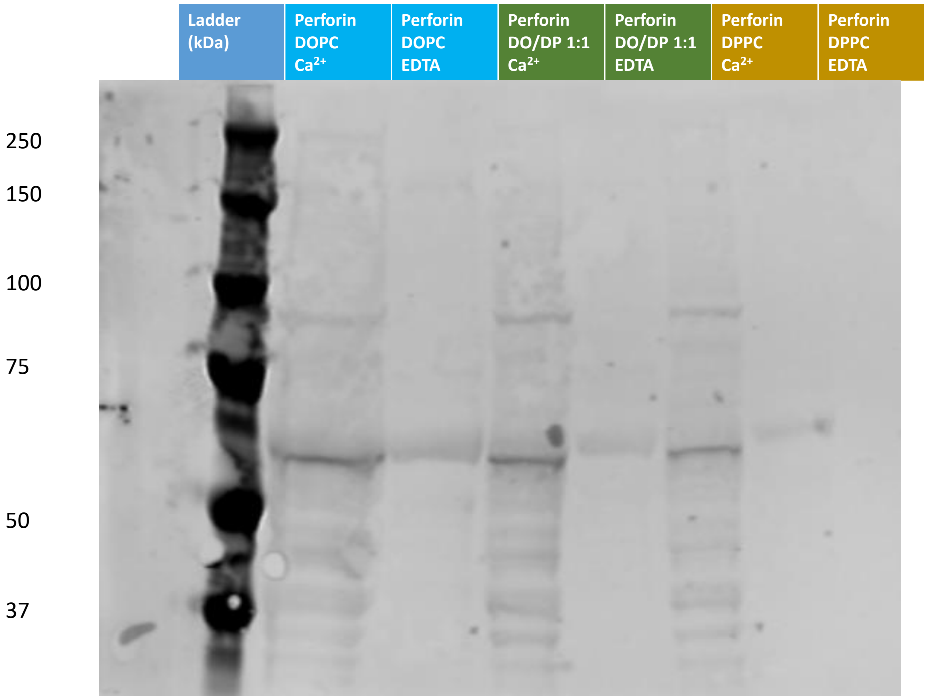
Ladder (kDa)
Perforin DOPC Ca2+
Perforin DOPC EDTA
Perforin DO/DP 1:1 Ca2+
Perforin DO/DP 1:1 EDTA
Perforin DPPC Ca2+
Perforin DPPC EDTA
250
150
100
75
50
37

# Raw pictures for Figure 5C

Three raw gel images used in Figure 5C are attached below:

Proteins in each samples: perforin only

Capture method: Odyssey CLx scanner

Molecular weight markers and loading order have been annotated without obscuring any data or background bands.

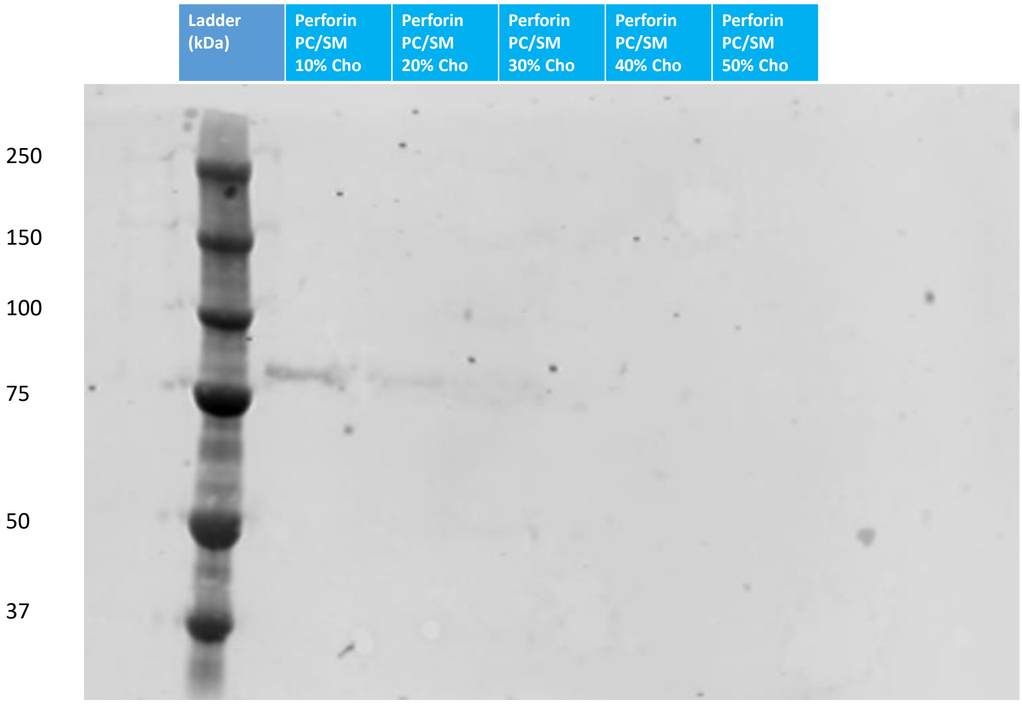

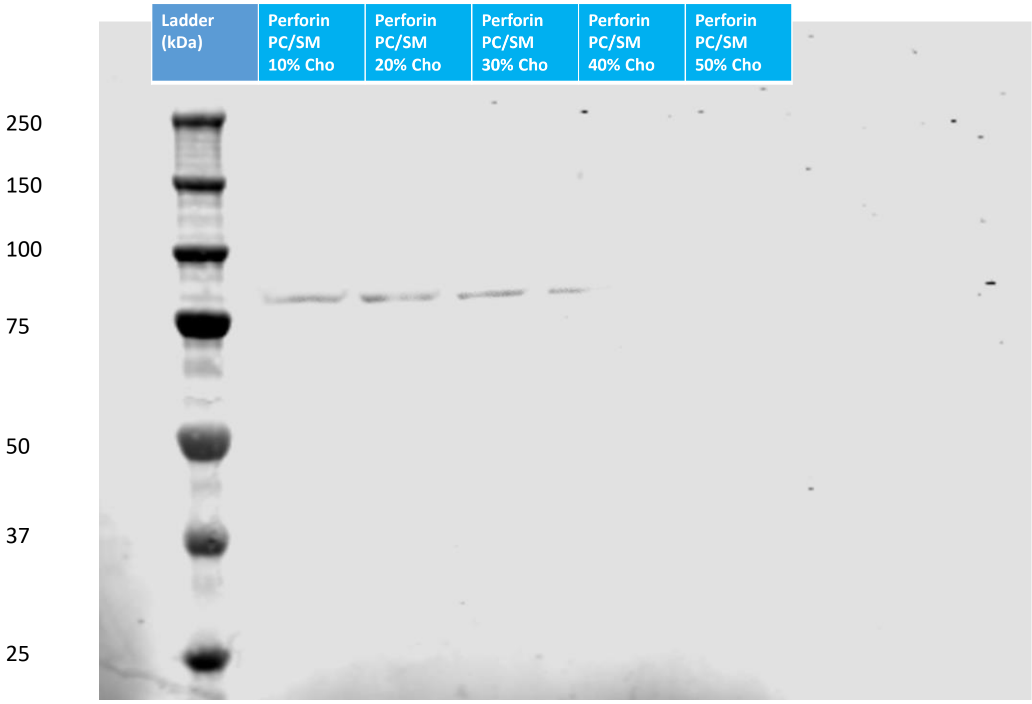
Ladder (kDa)
Perforin PC/SM 10% Cho
Perforin PC/SM 20% Cho
Perforin PC/SM 30% Cho
Perforin PC/SM 40% Cho
Perforin PC/SM 50% Cho
250
150
100
75
50
37
25

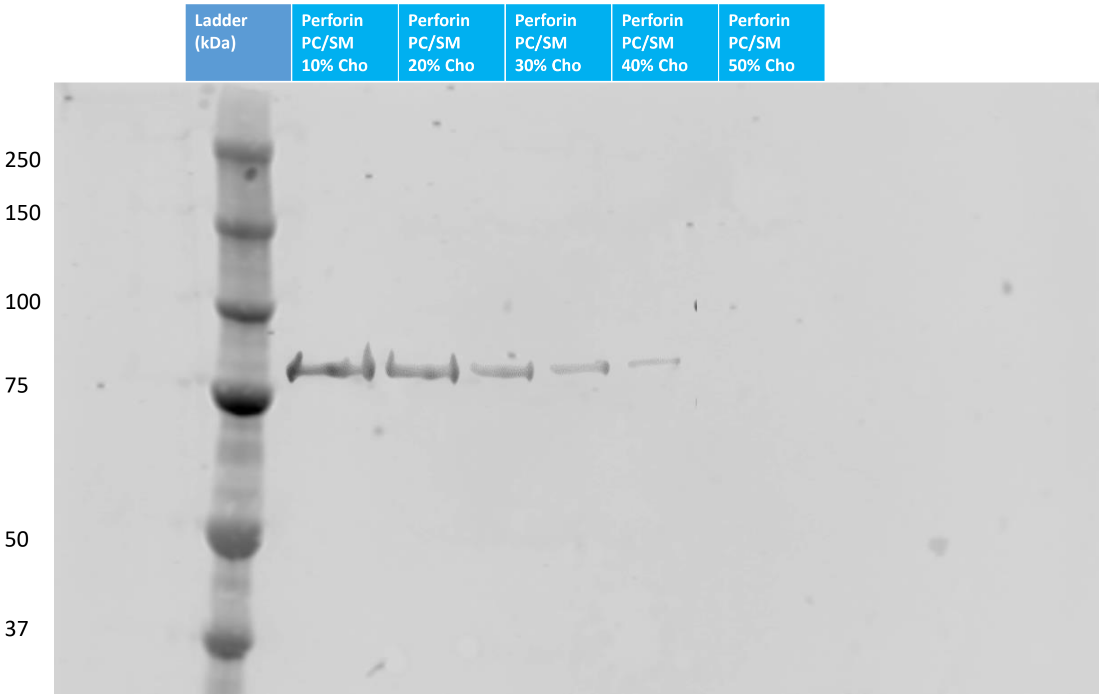
Ladder (kDa)
Perforin PC/SM 10% Cho
Perforin PC/SM 20% Cho
Perforin PC/SM 30% Cho
Perforin PC/SM 40% Cho
Perforin PC/SM 50% Cho
250
150
100
75
50
37

# Raw pictures for Figure S9

Raw and annotated gel images used in Figure S9 are attached below:

Samples: cell lysates

Capture method: Odyssey CLx scanner

Molecular weight markers and loading order have been annotated without obscuring any data or background bands.

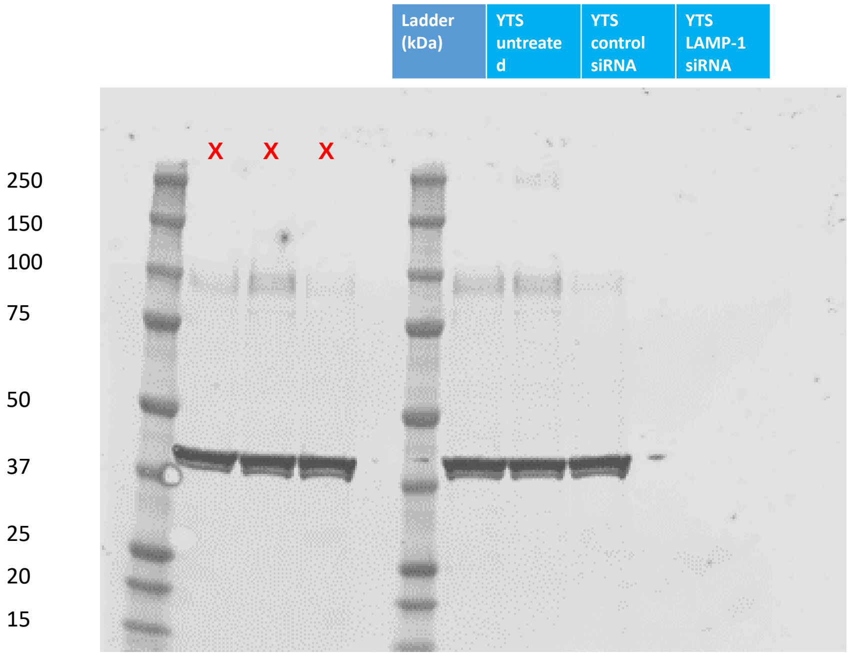

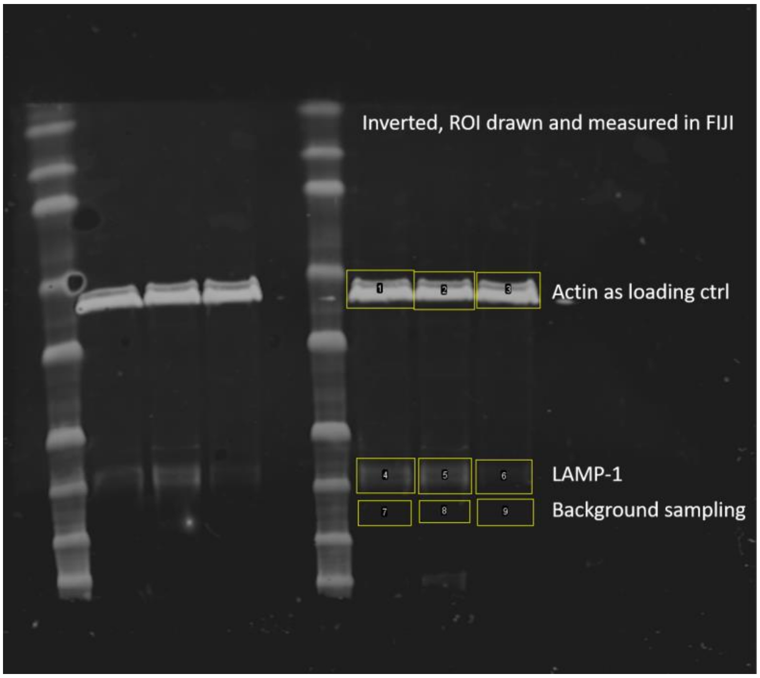
Inverted, ROI drawn and measured in FIJI
Actin as loading ctrl
LAMP-1
Background sampling
1
2
3
4
5
6
7
8
9
