# Supplementary material for: Degranulation enhances presynaptic membrane packing, which protects NK cells from perforin-mediated autolysis
Source: PLoS Biol. 2021 Aug 3;19(8):e3001328. doi: 10.1371/journal.pbio.3001328 (PMC8330931; doi:10.1371/journal.pbio.3001328)
Supplement: Attachment — Submitted filename: 20210421_Reply to reviewers.docx [file pbio.3001328.s012.docx]

**RESPONSE TO COMMENTS:**

We are grateful for the opportunity to respond to the comments provided. The comments are copied below verbatim and our responses are provided in blue text.

**EDITOR’S COMMENTS:**

Thank you very much for submitting your manuscript "Degranulation-enhanced presynaptic membrane packing protects NK cells from perforin-mediated autolysis" for consideration as an Initial Research Submission at PLOS Biology. Your manuscript has been evaluated by the PLOS Biology editors, an Academic Editor with relevant expertise, and by several independent reviewers.

In light of the reviews (below), we will not be able to accept the current version of the manuscript, but we would welcome re-submission of a much-revised version that takes into account the reviewers' comments. We cannot make any decision about publication until we have seen the revised manuscript and your response to the reviewers' comments. Your revised manuscript is also likely to be sent for further evaluation by the reviewers.

We are truly grateful for the opportunity to revise our work and appreciate the meaningful comments and reviewer feedback. We have worked hard over the past few months to improve our manuscript and have done our best to improve and revise it in response to all concerns and requests. We are grateful for all of the extremely useful points and suggestions and believe we are now able to present a substantially better manuscript.

In particular, reviewer #1 wants to see the results from 2 cell lines in separate graphs and with a minimum of 10 data points/cell line, has several questions about figure 2, target cell lysis rate, NK cell death at 16 h time point, statistics, and presentation of 5 donors separately. Reviewer #1 also asks about the lipid composition of target cells, whether NK cells form conjugates with target cells in the presence of EDTA, and wants you to tone down the conclusions about tumor cell scape, saying that you should use a second resistant tumor cell line to validate your results. Reviewer #1 and #3 have concerns about the NK cell resistance to perforin in the absence of degranulation, and about the specificity of the 7KC treatment, suggesting to repeat the experiment with target cells. Reviewer #2 wants you to discuss more thoroughly previous similar work. Reviewer #2 and #3 have concerns due to 7KC not only impacting membrane order. Reviewer #3 has concerns with the small effect of 7KC on NK-cell autolysis, asks what happens with a flipped ratio of E:T, says that you should present data for PI uptake in CD107a-negative cells, and have further questions about 7KC-treated cells. Reviewer #3 also says that the data ruling out LAMP-1 is weak, and asks whether you can add a control to figure 5. Please address also the rest of the reviewers' comments.

We appreciate editor’s effort in highlighting the critical comments and concerns from reviewers. We have designed and performed additional experiments, expanded our sample sizes, performed new analyses and added supporting data. All in an effort to answer these key questions as well as the others. Rather than summarize those key changes here we address them responsively below, but believe we have been able to meaningfully cover all of these subjects. Please note in extensive review of and in some cases additions to our spreadsheets (all provided via Dryad) some error bars in revised have changed although no changes in directionality or significance have. We are especially excited about an entirely new experiment that addresses many of the individual concerns in demonstrating the ability of one NK cell line to now be able to kill another that has been depleted of lipid ordering. We have additionally modified our manuscript, figures and legends, to further clarify ambiguities and address any grammatic and typographic errors.

We expect to receive your revised manuscript within 3 months. Please email us (plosbiology@plos.org) if you have any questions or concerns, or would like to request an extension. At this stage, your manuscript remains formally under active consideration at our journal; please notify us by email if you do not intend to submit a revision so that we may end consideration of the manuscript at PLOS Biology.

We would again like to thank the editor and reviewers for the thorough feedback and very thoughtful suggestions. We are motivated to further strengthen our work and look deeper to the biological mechanism behind the self-protection of NK cells during target engagement. We are hopeful that with these suggestions that our work will have obtained a priority suitable for the readership of PLoS Biology.

**REVIEWERS’ COMMENTS:**

Reviewer #1:

This articles addresses the very important question of how NK cells (and possibly other cytotoxic lymphocytes) are protected against their own weapons, in particular perforin which, once released to the immunological synapse, promotes the formation of holes in the target cell membrane.

Overall this is a very well written and interesting article. The introduction is well documented and focused on the question addressed in the subsequent experimental work. The methodology is appropriate and the experimental work is of great quality. The conclusion (and title) that « Degranulation-enhanced presynaptic membrane packing protects NK cells from perforin-mediated autolysis » are supported by a significant amount of results. I listed below several concerns that need to be addressed before publication.

We are grateful to the reviewer for their careful review of our work and the positive perception. The feedback provided was extremely helpful and has motivated us to meaningfully improve our manuscript.

Figure 1.

a) The data obtained with YTS and NK-92 are depicted in the same graphs (panels C and F and possibly panel I). It would be nice to see the results of the two cell lines in separate graphs.

b) With this regard, a minimum of 10 data points/cell line for proper statistical testing would be appreciated.

We apologize for having created any confusion and for lacking in clarity. Figure 1C, F and I had only contained measurements using YTS cells (n>10). We have now clarified our presentation and to address the reviewer’s request we have added new subfigures that separately present data from NK92 cells, with a minimum of 10 data points.

c) Is the unpaired t-test appropriate here?

We thank the reviewer for asking this question and are able to provide additional clarification. In Figure 1C and F, for each NK cell, we obtained lipid packing densities from its synaptic and non-synaptic regions, resulting in pairs of observations. Thus, we used a paired t-test in comparing these dependent measurements from the same cell. We have now clarified this in the legend.

In Figure 1 I, for each NK cell, we only acquired the lipid packing density of its pre-synaptic membrane. Thus, to compare the independent measurements among different cells, we utilized the unpaired t-test test.

Figure 2.

a) Is a target cell lysis rate of 30% the max. cytolytic capacity of NK cells even at high E:T ratios?

In our hands, using our particular methods and in a 4-hour co-incubation NK cells kill about 20~30% target cells at a high E:T ratio. If longer co-incubation times are allowed, the maximum cytolytic capacity can reach to about 50% (as shown in figure 2D). These findings are consistent with those in our other publications using these NK cell lines (such as reference #2). We do appreciate that some laboratories will report either higher or lower maximal cytotoxic capacity and we believe this relates to different technical approaches to the assay.

b) Fig. 2D, the values for the 16 hours coculture are surprisingly lower than 4 or 8 hours and this is not reported in the result section. Could this be explained by the higher rate of YTS death shown for the same time point in Fig. 2E? By the way, how do you explain the increase of NK cell death in the 16 hours cytotoxicity assay (while other incubation times are all similar)? Alternatively, did the authors account for the increase in NK cell death and adjust the target cell lysis accordingly?

We appreciate the careful review of this subfigure. The decrease of cytotoxicity in the 16-hour co-culture was reproducible via Cr^51^ release assay. When we measured cytotoxicity of NK cells using flow cytometry-based assay, the cytolytic capacity in 16-hour co-incubation was actually higher than the 8-hour one. We presume that the difference is specific to the ^51^Cr assay and conditions used in that assay with that particular extended timeframe. This might be one of the reasons that shorter timeframes in this particular assay are most frequently utilized. We have now added this observation and additional explanation.

We did not adjust target cell lysis for increased effector cell death rates in our manuscript. While in longer timeframes should also dampen increases in target cell lysis, one could conceive a time point where the effector cell death disproportionally affects serial target cell killing. The effect of NK cell autolysis on serial killing is an interesting and potentially relevant concept that we would like to study further. We have raised this possibility in our revised manuscript.

c) Healthy donor derived NKs: because there is significant heterogeneity between donor NK cells, I suggest to show each set of data (i.e. for each donor) in addition to (as a supplementary Figure?) or instead of the averages values. Ideally, five donors should be used. Finally, it would be important to check and report target cell killing with eNKs as well (similar to what has been shown with NK cell lines, Figure 2A and B).

We agree with the reviewer regarding inter-donor heterogeneity and are happy to expand the sample size in response to this particular request. We have now performed new experiments with additional donors to be able to present the results from 5 different donors. We have also created a new supplementary figure (Fig. S3) to separately show each set of data as requested. The experiments were performed with purified NK cells and not whole PBMC and we have clarified this and added the cytotoxicity data of the purified NK cells to Figure 2C to be in parallel with Figure 2A and 2B as requested.

d) Statistical significance is sometimes missing. Not sure which test should be used.

We appreciate the careful review. We now have added more details into figure legends to clarify the tests used and statistical significance. We also have worked with biostatisticians to have all statistical tests further checked. Specifically, Wilcoxon signed rank test was used for the comparison between curves in 51Cr release assays. Paired and unpaired student t test were used for group comparisons in other assays.

Figure 3.

Do NK cells form conjugates with target cells in the presence of EDTA?

We thank the reviewer for this question and we have revisited the effect of EDTA on conjugation formation in response. Specifically, we measured conjugation between NK and target cells with or without presence of EDTA (the same concentration with Figure. 3C and D) and did not find any significant difference. We have provided this negative result below for the reviewer, and added a citation in the manuscript. We would be happy to add this result to figure 3 or supplementary data if desired.

Figure 4.

a) Panel D, NK cells were exposed to free perforin. Without degranulation events, the NK cell membrane shows no increase in lipid order (which was proposed to drive resistance). Thus, how the authors explain NK cell resistance to perforin (in the absence of degranulation)? Is it possible that 7KC treatment destroys the overall cell membrane structure of resting NK cells rendering them more vulnerable to perforin? Testing of membrane leakiness after 7KC treatment with cytoplasmic dyes could provide more insight into the specificity of this treatment on perforin susceptibility.

We are grateful for the reviewer’s thoughtful consideration of this experiment and for the suggestion of this important control experiment. Importantly, we did notice that membranes of resting NK cells have higher packing densities than target cell membranes, implying they should have a higher baseline resistance to free perforin. This relatively resistant membrane has also been observed in both resting NK cells and CTLs, using patch clamp technique*. While historical, Persechini and Young reported a ~10-fold higher working threshold of free perforin on the resting NK and CTL membranes. We have now accentuated our observation and this reference and have provided additional discussion to the manuscript.

The reviewer’s question regarding any altering osmotic effect of 7KC on NK cell membrane is also well taken as is the reviewers’ experimental suggestion. Using Calcein Green (a cytoplasmic dye) as leakage indicator, we have re-examined the membrane integrity of NK cells after 7KC-pretreatment. We found within the concentration range shown in Figure 3 and including that used throughout our work, 7KC-treated NK cells have similar membrane integrities to those of untreated cells. We have now included this finding in a new Figure S4 and in our revised text.

** Persechini PM, Young JD, Almers W. Membrane channel formation by the lymphocyte pore-forming protein: comparison between susceptible and resistant target cells. J Cell Biol. 1990 Jun;110(6):2109-16.*

b) A repetition of this experiment with target cells would give indications whether NK cells have intrinsically a cell membrane of higher lipid order than other cells, providing them with a resistance "bonus".

We appreciate the suggestion and are compelled by the “resistance bonus” concept. Thuys, we have repeated this experiment as requested with the target cell line 721.221. Here we found that 7KC did not further sensitize these target cells to free perforin. We have added this result to Figure 4 as a new panel, E. Furthermore, the recent CTL study reported a similar phenomenon in target cell lines EL4 and P815**. Taken together this indicates that NK cells do have intrinsically higher resistance to free perforin. In addition to the new result we have also added discussion and underscored the result of Rudd-Schmidt, et. al.

Following the resistance bonus theme and despite their somewhat inherent resistance to free perforin, NK cells and CTLs are still interestingly still somewhat susceptible to perforin attack from other cytolytic cells. In addition to publications regarding NK cells*** and CTL**** fratricide, which we have now added and discussed in this context, we have now also designed a new experiment which addresses the resistance bonus as well (see also below in response to the Figure 7 discussion). Specifically we now show that NK92 can kill YTS cells in co-incubation experiments to a slight degree which is enhanced by treating the YTS with 7KC (New Figure 7, H). Thus it is likely that the advanced self-protection mechanism of NK cells against secreted perforin during degranulation is still of practical benefit. While this particular experiment is also meant to address some improvement to Figure 7, we think it also contributes to the reviewer’s point and added discussion of it in this regard to the manuscript.

******** *Rudd-Schmidt JA, Hodel AW, Noori T, Lopez JA, Cho HJ, Verschoor S, Ciccone A, Trapani JA, Hoogenboom BW, Voskoboinik I. Lipid order and charge protect killer T cells from accidental death. Nat Commun. 2019 Nov 27;10(1):5396.*

********* *Binder C, Sellberg F, Cvetkovski F, Berg S, Berglund E, Berglund D. Siplizumab Induces NK Cell Fratricide Through Antibody-Dependent Cell-Mediated Cytotoxicity. Front Immunol. 2021 Feb 11;12:599526.*

***** Fleischer LC, Spencer HT, Raikar SS. Targeting T cell malignancies using CAR-based immunotherapy: challenges and potential solutions. J Hematol Oncol. 2019 Dec 29;12(1):141.*

Figure 6.

As previously requested for figure 2: could the authors provide the data obtained for each primary NK cell donor. Ideally, please include 2 additional donors to better take into account inter donor heterogeneity.

We again appreciate the reviewer’s point regarding inter-donor variability of human NK cells and in response we have expanded our sample size to 5 and generated a new supplementary figure (Fig S3) to separately show each individual donor’s ex vivo NK cell data as requested.

Figure 7.

a) The legend is confusing. K562 and MB-231 cells are used in both panels E and F. Please check the figure legend.

We apologize for confusion caused in the legend and we have edited the figure legend to clarify.

b) The susceptibility of MB-231 is only minimally increased following 7KC treatment. Accordingly the role of densely-packed post synaptic lipid membrane in tumor cell escape should not be overestimated. In the discussion section, the authors are invited to tone down their conclusions and clearly state that although lipid density might contribute to tumor cell resistance against NK cells, it can likely not account for most of the resistance.

The reviewer makes an important point and we have edited and toned down our conclusions. We are also hopeful that the new experiment performed in response to the reviewer’s next comment is additionally useful in this regard (new Figure 7, H).

c) Do other (resistant) cancer cells respond to NK cell attack in the same way? Ideally a second resistant tumor cell line should be used to validate the results obtained with MD-231, in particular since the increase in susceptibility is not as high.

d) What is the fate of target cells that generate densely packed postsynaptic membranes? Do they survive?

The reviewer’s point about generalizability of this mechanism is of course well taken. While we have been actively developing plans for this being part of our lab’s future work, we do want to try and address this meaningful comment more robustly as well as to be directly responsive to the request. As we alluded to above, we acknowledge that NK cells themselves while susceptible to some fratricide are of course resistant to their own lytic machinery as is the focus of our work. That said, the NK cell lines we use in our work are derivative from tumors themselves and can thus be considered as a resistant cancer cell. Thus, we performed new experiments in which we treated the YTS cell as a target cell and the NK92 cell as an effector. This has become very interesting to us and we wish that we had thought to do it a long time ago, but thank the reviewer for the prompts (we are also not aware of other examples of these particular experiments in the literature). Along these lines, we observed modest killing of YTS by NK92 after 4-hour co-incubation. These features allow YTS cells at least in part to be considered in this context as a NK-resistant cancer cell model to extend the findings with MB-231 cells. We therefore performed the parallel experiment and found that 7KC pretreatment did in fact sensitize YTS cells to NK92 killing akin to the effect of 7C upon MB-231 cells. We have added this new experiment and corresponding text to the revised manuscript.

e) Is there a higher autolysis of NK cells when cultured with MDA-MB-231 cells due to the denser postsynaptic membrane?

We again appreciate the reviewer’s insight and suggestion. We have now measured NK autolysis when they are attacking MB-231 cells. We did not find a significant increase of NK cell death, suggesting that the self-protective mechanism of NK cell remains effective even when facing a similarly dense postsynaptic membrane. We have added this result to Figure 7 (as Figure 7H) along with corresponding discussion. Presumably dense membranes are fundamentally more resistant and remain so even with perforin in the synaptic cleft.

Additional points:

a) I suggest the authors to more explicitly explain how LAMP-1 has previously been suggested to provide NK cells with perforin protection (line 80). Would it be relevant to refer to #34 here?

We thank the reviewer for the recommendation and in an effort to further clarify, we have now added more background regarding LAMP-1 and its proposed roles to the introduction, and cite the Cohnen, et al, reference as requested.

b) I don't know what PLOS Biology’s policy on siRNAs is but most journals request to validate the results from gene KD with at least two different siRNAs. The "RNA interference" subsection in the Methods section only refers to the siRNA strategy for PRF1, while additional genes are knocked down using siRNAs.

We appreciate the reviewer’s comment and agree that specificity of siRNA is vital for our knockdown experiments. For siRNA knockdown in this work, we used siRNA cocktails (Dharmacon siRNA SmartPool) that contain 4~6 separate siRNAs targeting different ORFs of targeted genes. We have clarified this approach in the methods of our revised submission.

c) Line 635: the authors should refer to the original study (not only to a review article).

We have edited the text to cite the original article as requested.

d) Please check the statistical tests used throughout the article.

We have worked with biostatisticians to have statistical tests further checked.

Reviewer #2:

Overall, this manuscript describes what appear to be carefully done and extensive experiments probing (using membrane polarity sensitive dyes) and manipulating (using 7KC) the membrane order at the immune synapse, tying membrane order to the lytic activity of perforin in NK T-Cells.

We thank the reviewer for the positive perception of our work and for all the helpful feedback, which we have utilized to improve our manuscript. We are grateful for the opportunity and hope that our revisions and additions are met with the enthusiasm with which they are provided.

I have 2 main comments/concerns:

The first being that the idea presented here is already present in the literature. This in itself is not a major problem, but I found it strange that it was not discussed more thoroughly. Namely, reference 36 of the manuscript is titled "Lipid order and charge protect killer T cells from accidental death" and has a similar thesis to the present submission, although many of the experimental approaches differ.

As we have been working on this question for many years we were disappointed that some of the originality of our work might be lost and appreciate in retrospect that we should have been more robust in discussing the work of Rudd-Schmidt, et. al. We apologize for this oversight. We are grateful, however, that the reviewer sees the benefit in many of our distinct approaches and emphasis upon NK cells. We have added more discussion of the reported findings in CTLs in our revised manuscript and their implications to our experiments.

My second main concern is the assertion that treatment with 7KC only impacts membrane order, which is certainly not the case. This lipid is added in a way that will also lead to the extraction of other lipids, especially cholesterol, and this could impact the observed processes in ways that do not depend exclusively on 'ordering'. This again is not necessarily a major problem as long as it is discussed appropriately. Ideally, experiments would also be done using other treatments that impact ordering in alternate ways, such as by adding bile acids or alcohols, feeding cells alternate fatty acids, or by altering lipid metabolism.

A possible way to address both concerns would be to narrow the scope of the current manuscript to more centrally focus on the specific inhibitory role of 7KC in the title and abstract.

The reviewer makes an excellent and well received point. We have added some additional discussion as requested, but also wanted to try and be experimentally responsive as well. Thus, we attempted an alternative experimental approach to manipulate membrane properties. In this regard we have applied Nystatin, a cholesterol sequestering agent, to alter the packing of NK cell membrane. Nystatin has been described to disrupt cholesterol-rich micro-domains on plasma membrane without depleting cholesterol from the membrane*. Therefore it provides an alternative way to allow us manipulating lipid packing without having the same effect as 7KC in changing lipid composition. Albeit with lower molar efficiency compared to 7KC, Nystatin induced autolysis of NK cells. We have added this new experiment as a new supplementary figure (Fig S4) along with discussion to the revised manuscript.

** Foster LJ, De Hoog CL, Mann M. Unbiased quantitative proteomics of lipid rafts reveals high specificity for signaling factors. Proc Natl Acad Sci U S A. 2003 May 13;100(10):5813-8.*

Reviewer #3:

This is a highly intriguing study from Li and Orange revealing formation of densely packed, highly ordered lipid membranes on NK cells at pre-synaptic sites of lytic granule fusion that prevent perforin binding and autolysis. They reveal that the unique lipid composition of lytic granules contributes to and/or reinforces this protection. Moreover, they show that an NK-cell resistant breast cancer cell line forms similar densely packed post-synaptic lipid membranes that contributes to evasion of NK-cell killing. The combination of fluorescent lipid packing sensors and microscopy is an asset that permits the additional strength of analysis across multiple NK-cell lines, primary NK cells, and tumor targets.

We thank the reviewer for the positive perception of our work and for the meaningful feedback with which we have worked to add needed insights, new data and improvements to our manuscript.

Concerns

1. While the effect of 7KC on high lipid order appears absolute in Figure 1, the effects on NK-cell autolysis in Figure 2 and elsewhere (notably Figure 7) are very small (this mechanism appears to protect 5% of NK cells). Intriguingly, these effects increase with effector cell density, potentially implicating fratricide over autolysis. What happens in an experimental setup with flipped ratio (1:10 E:T) that triggers greatest degranulation among large number of NK cells? While Figure 3G suggests degranulation cells appear to be most susceptible to death, data for PI uptake in CD107a-neagtive cells should be presented. In related queries, are the line colors mixed up in right graph in Figure 4A where NK92 survival would expected to be worst for 7KC-treated cells, which obfuscates the effect of CMA in this experiment. Finally, it is not clear why the measurement of 721.221 cell death in Figure 2D would decrease between 8 and 16 hours.

We thank reviewer for the very detailed feedback and will attempt to address each point raised.

With regards to the size of the protective effect of having ordered lipid, the lysis of NK cells after 4h coincubation with target cells is effectively doubled after 7KC treatment. While in theory this could go further, it is relevant to point out that target cells (which are not lipid ordered and highly susceptible) are only maximally lysed to about the same percentage. This suggests that the percent of cell destruction in this 4 hour assay at these levels is likely a feature of the assay itself and not of the susceptibility of the cell being killed. This point is accentuated when the incubation times were extended in in figure 2E, where the lysis of 7KC-treated NK cells underwent greater lysis after a 16-hour co-incubation (up to 70%). This contrast emphasizes the technical nature of the 4h assay and potentially the assay itself and suggests that lipid ordering can be a majority protective mechanism for the majority of NK cells during their degranulation.

The suggestion to further delineate autolysis vs fratricide using the ^51^Cr assay is similarly technically limited as the low numbers of labeled NK cells or the high numbers of target cells needed to perform the flipped ratio experiment challenge the sensitivity of the assay (in other words we would need so few NK cells as to challenge the limits of detection or too many target cells as to crowd the microwell). While we have great respect for the point being made, we hope that the CD107a experiment (below) at least partially addresses the concept of autolysis and we have added some discussion of limitations and fratricide. We have also adjusted our language in a number of places in the manuscript to be inclusive of additional explanations.

We appreciate the additional suggestion made by the reviewer regarding Figure 3G, especially in light of the technical limitations we face with the ^51^Cr-release assays. We have thus added data for PI uptake in CD107a-negative cells and have redrawn this subfigure accordingly. They do continue to lend support the point that the degranulating cells are the ones dying, providing some credence to autolysis. Again, we have also added some explanations and alternatives to our text as well.

We want to thank the reviewer for pointing out the error in labeling of Figure 4A, we apologize and have made the correction.

As for the decrease of cytotoxicity in the 16-hour co-culture, it is a reproducible result and speaks again to a likely technical limitation of the ^51^Cr release assay and is perhaps why shorter timeframes are standard practice. That said, when we measured cytotoxicity of NK cells using flow cytometry-based assay, the cytolytic capacity in 16-hour co-incubation was indeed higher than the 8-hour one and we have further explained this result in our revised manuscript.

2. There is confusion about how Figure 4D fits with overall mechanism. If the synaptic formation of dense ordered lipid membranes that is enhanced by degranulation and is protective versus perforin, then what is the mechanism at play in resting NK cells treated with recombinant perforin? Is this different in activated NK cells?

We appreciate reviewer’s comment and are grateful the opportunity to further discuss the result and how we believe it relates to the nature of NK cell membranes. We did notice that membranes of resting NK cells have higher packing densities than target cell membranes, implying they should have a higher baseline resistance to free perforin (a result corroborated in CTL by Rudd-Schmidt, et. al*). In addition to adding some discussion and clarification of this, to further accentuate this inherent characteristic of NK cells, we have now repeated the free perforin experiment with the target cell line 721.221 for contrast. Here we found that 7KC didn’t further sensitize these susceptible target cells to free perforin (a result further corroborated in in EL4 and P815 cells by Rudd-Schmidt, et. al*). We have added this result (New Fig. 4D) to our revised manuscript. We take these results indicate that NK cells do have intrinsically higher resistance to free perforin. This relatively resistant membrane has also been observed in both resting NK cells and CTLs, using patch clamp technique**. Persechini and Young reported a ~10-fold higher working threshold of free perforin on resting NK and CTL membrane. We have added this reference and related discussion to our revision as well. Finally, despite having a baseline resistance to free perforin, NK cells and CTLs are still susceptible, albeit minimally, to perforin attack from other effector cells (i.e., fratricide). We have now added citations to previous reports of fratricide of NK cells*** and CTLs****, but suggest that while inherently resistant, the advanced protection that derives from increased lipid ordering at the presynaptic membrane and focal increases in lipid ordering at sites of degranulation are still relevant contributions to NK cell protection. We have added additional discussion to this effect in an effort to delineate and further clarify inherent and enhanced resistance.

******* *Rudd-Schmidt JA, Hodel AW, Noori T, Lopez JA, Cho HJ, Verschoor S, Ciccone A, Trapani JA, Hoogenboom BW, Voskoboinik I. Lipid order and charge protect killer T cells from accidental death. Nat Commun. 2019 Nov 27;10(1):5396.*

*** Persechini PM, Young JD, Almers W. Membrane channel formation by the lymphocyte pore-forming protein: comparison between susceptible and resistant target cells. J Cell Biol. 1990 Jun;110(6):2109-16.*

********* *Binder C, Sellberg F, Cvetkovski F, Berg S, Berglund E, Berglund D. Siplizumab Induces NK Cell Fratricide Through Antibody-Dependent Cell-Mediated Cytotoxicity. Front Immunol. 2021 Feb 11;12:599526.*

***** Fleischer LC, Spencer HT, Raikar SS. Targeting T cell malignancies using CAR-based immunotherapy: challenges and potential solutions. J Hematol Oncol. 2019 Dec 29;12(1):141.*

3. On line 135, the results in Figure S2 are said to be similar to non-synaptic regions in Figure 1, but this is not readily apparent from the visible spectra representing general polarization values (bright yellow in unconjugated cells and dark orange to red in non-synaptic regions). Minimally this could be clarified with presentation of mean/SD values of GP for unconjugated cells. Is it feasible to capture images of lipid order in synapse and 7KC disruption (Figure 1) in primary NK cells (only tested in killing)? This would be a major plus but understandable if primary cells are less amenable to the imaging techniques employed.

We are grateful for this careful review and appreciate that there were some ambiguities and confusion in the previous approach to Figure S2. Specifically, with regards to the LUTs which were arbitrary and not linked between the figures. To ameliorate we have revised Figure S2, where we now show conjugated and unconjugated cells in the same field of view. This hopefully removes a need for relative comparisons between Figures 1 and S2 and emphasizes the difference in membrane packing between conjugated and unconjugated cells of the same type in the same experiment. Thus, any difference in their membrane packing can be presented uniformly and accurately. As the reviewer alludes, primary NK cells are indeed less tolerant to the conditions used for lipid packing imaging (multi-dye labeling, unfavorable imaging medium, etc.). Despite this we have tried our best to fulfill reviewer’s suggestion. That said, we were unable to maintain sufficient robustness in the ex vivo NK cells themselves under these conditions and collect enough repeats in the provided time window to allow for meaningful downstream analysis.

4. The data ruling out LAMP-1 is weak. How do we know CD107a neutralizing antibodies worked as advertised? What is a positive control for this reagent? Likewise, in LAMP-1 knock-down cells, the LAMP-1 expression levels within granules (highly enriched) should be assessed as well as cell surface levels of CD107a after degranulation to ensure the knockdown was effective where it matters.

We appreciate the focus of the reviewer and want to emphasize that it was not our intent to rule out a role for LAMP-1 in protection, but more to provide relative comparison to the protection provided by lipid ordering. To this effect we have altered our language along these lines. While we do not have a better means of validating the antibody given that we obtained a negative result for both the antibody and knock-down we did attempt a variation of the validation experiment recommended by the reviewer. Specifically, we measured LAMP-1 levels in lytic granule using quantitative confocal imaging. After siRNA transfection, we found that LAMP-1 levels were reduced substantively both globally and inside the lytic granules specifically. We have now added this result (image and quantitation) to Figure S9.

5. The approach to dissecting the role of dense ordered lipid membranes is restricted to use of 7-ketocholesterol. This is somewhat problematic given the toxicity of this drug in cells and the difficulty of determining that the only relevant effect of 7KC is the desired disruption of dense lipid formations rather than anti-mitotic or effects on mitochondrial membrane potential. The authors make several attempts to rule out direct toxicity of 7KC, which are good but could be strengthened. For example, not clear what cell density is used in Figure 3A (incubation of NK cells without targets), but would be important to use NK cells at same density as used in 10:1 E:T wells and to include controls of activated NK cells (without targets) to determine if a combination of 7KC and activation signaling is problematic for NK cells. Ideally, a second non-7KC method for disrupting these dense lipid formations if possible would be a huge improvement. A related question is whether the smaller size of 7KC-treated cells in Figure 1D/E is a consistent feature and how this might impact other measurements in this study?

We thank the reviewer for this critical and helpful feedback regarding ways to improve our use and presentation of 7KC treatments. As for the density of cells most experiments were performed using the same conditions but this was not possible in the imaging experiments owing to the need for spacing cells to allow for visualization and similarly for flow cytometry to allow for acquisition without clogging fluidics. As for the activation state of the NK cells, we appreciate this suggestion and we have now performed and include controls in which we treated resting NK cells with both 7KC and activating reagents (PMA+Ionomycin). This should address the concern of 7KC effects as a feature of activating state as PMA+Ionomycin is very strongly activating. Here we did not find increased death as measured by leakage of Calcein dye in activated 7KC-treated NK cells, suggesting that the combination of 7KC and NK cell activation signaling did not contribute to NK cell death (in the absence of a lytic synapse/synaptic cleft, or very high free perforin concentrations). We have added this result to Fig. S4, C and corresponding text to the manuscript.

We also appreciate the reviewer’s concern over our reliance upon 7KC and its potential unique effects on the NK cell membrane including ones we might not be aware of. We have added some additional discussion to this effect but agree with the reviewer that it would be ideal to present an alternative method to manipulate membrane properties. In an attempt to address this directly we have utilized Nystatin, a cholesterol sequestering agent, to alter the packing of NK cell membrane. It has been reported that Nystatin can disrupt cholesterol-rich micro-domains on plasma membrane without removing cholesterol from the membrane*. Thus, Nystatin provides an alternative means of manipulating lipid packing without changing lipid composition. Although with less molar efficiency compared to 7KC, Nystatin treatment similarly induced the death of NK cells when they were combined with susceptible target cells. We have added these new experiments in to Figure S4 along with corresponding text and discussion.

As to the reviewer’s final question about effects of 7KC on NK cell size, we performed analysis and measurements of 7KC-treated cells. We did not find any significant trend in impact upon cells size caused by 7KC. We have added this result as part of the text in the methods section in 7KC treatment.

* Foster LJ, De Hoog CL, Mann M. Unbiased quantitative proteomics of lipid rafts reveals high specificity for signaling factors. Proc Natl Acad Sci U S A. 2003 May 13;100(10):5813-8.

6. Is there any potential control (i.e. loading control) for Figure 5? Disconcerting to see the shrinking numerator without any denominator.

We appreciate the reviewer’s concern regarding potential control of blots for Figure 5. As the Western blots in Figure 5 are liposome binding assays using purified perforin, there is no other protein that can serve as loading control. Specifically because in this assay only proteins that are precipitated with the liposome can be measured and perforin was the only added protein. If a protein were to be intentionally added as an external loading control, it must bind to any liposome membranes uniformly. While this is theoretically possible it would require advancement of the known biology of a selected control protein and was not something were able to accomplish during the time of this response period. Hopefully the two different types of liposomes used and varying content of the liposomes provide the specificity to the point made about perforin binding best in a reductionist system to lipid ordered membranes. Additionally our presentation of this assay without an additional added protein to allow for a Western blot loading control is consistent with published methodlogical recommendations for this particular variation of this assay*. We have now added additional discussion and the citation of this practice with methodological reference.

**Julkowska MM, Rankenberg JM, Testerink C. Liposome-binding assays to assess specificity and affinity of phospholipid-protein interactions. Methods Mol Biol. 2013;1009:261-71.*

Minor points

1. There is a lot of interpretation and discussion contained within the results section that should ideally be restricted to the discussion.

We have now reorganized those discussions and interpretations as suggested.

2. Typo in Figure 1F legend (p=12, presumably meant to be 0.12). This value should be presented in place of ns on the figure image itself. All ns should be replaced with actual values.

We appreciate this careful review. This typo has been fixed.

3. If p<0.0001 is used a 'lower limit', then p<0.001 (Fig 1C), p<0.01 (Fig 4C), and p<0.05 (multiple spots) should be replaced with actual p values.

We have updated our figures and added additional details to figure legends to clarify the tests used and statistical significance.

4. Colors in line graphs a bit hard to discern, not to mention for any readers who are colorblind.

We agree with reviewer’s suggestion and have now reselected colors and adjusted line thickness to better distinguish different lines.

5. Please put a label distinguishing the two pie charts in Figure 6C.

We thank this careful review have now added those labels for Fig.6C.

6. Statement on line 539 about GM3 should be cited to reference Table S1 since this information is not clear in Figure 6.

We have now redirected this statement to table S1.

7. Control I and II labels in Figure 6E should be better defined (these are two non-synaptic regions apparently?)

We have now changed these labels to “Non-degranulation site” to clarify this ambiguity.

8. Are bars switched in right bar graph in Figure S4?

We appreciate this careful review, and we have corrected this error.

9. Several images in Figure S5C and E are not discernable (appear teal)

Those images were auto-contrasted using image J during processing. This contrast setting was kept to show readers that there is indeed no perforin signal even under the most extreme contrast setting.
