# Supplementary material for: Degranulation enhances presynaptic membrane packing, which protects NK cells from perforin-mediated autolysis
Source: PLoS Biol. 2021 Aug 3;19(8):e3001328. doi: 10.1371/journal.pbio.3001328 (PMC8330931; doi:10.1371/journal.pbio.3001328)
Supplement: Peer review history

# Editor report: Decision Letter 0

**DOI:** 10.1371/journal.pbio.3001328.r001

**Authors:** Paula Jauregui, PhD

7 Dec 2020

Dear Dr. Orange, 

Thank you for submitting your manuscript entitled "Degranulation-enhanced presynaptic membrane packing protects NK cells from perforin-mediated autolysis" for consideration as a Research Article by PLOS Biology.

Your manuscript has now been evaluated by the PLOS Biology editorial staff [as well as by an academic editor with relevant expertise] and I am writing to let you know that we would like to send your submission out for external peer review.

However, before we can send your manuscript to reviewers, we need you to complete your submission by providing the metadata that is required for full assessment. To this end, please login to Editorial Manager where you will find the paper in the 'Submissions Needing Revisions' folder on your homepage. Please click 'Revise Submission' from the Action Links and complete all additional questions in the submission questionnaire.

Please re-submit your manuscript within two working days, i.e. by Dec 09 2020 11:59PM.

Login to Editorial Manager here: https://www.editorialmanager.com/pbiology

During resubmission, you will be invited to opt-in to posting your pre-review manuscript as a bioRxiv preprint. Visit http://journals.plos.org/plosbiology/s/preprints for full details. If you consent to posting your current manuscript as a preprint, please upload a single Preprint PDF when you re-submit. 

Once your full submission is complete, your paper will undergo a series of checks in preparation for peer review. Once your manuscript has passed all checks it will be sent out for review. 

Given the disruptions resulting from the ongoing COVID-19 pandemic, please expect delays in the editorial process. We apologise in advance for any inconvenience caused and will do our best to minimize impact as far as possible.

Feel free to email us at plosbiology@plos.org if you have any queries relating to your submission.

Kind regards,

Paula

---

Paula Jauregui, PhD,

Associate Editor

PLOS Biology

# Peer review: Decision Letter 1

**DOI:** 10.1371/journal.pbio.3001328.r002

**Authors:** Paula Jauregui, PhD

13 Jan 2021

Dear Dr. Orange,

Thank you very much for submitting your manuscript "Degranulation-enhanced presynaptic membrane packing protects NK cells from perforin-mediated autolysis" for consideration as a Initial Research Submission at PLOS Biology. Your manuscript has been evaluated by the PLOS Biology editors, an Academic Editor with relevant expertise, and by several independent reviewers.

In light of the reviews (below), we will not be able to accept the current version of the manuscript, but we would welcome re-submission of a much-revised version that takes into account the reviewers' comments. We cannot make any decision about publication until we have seen the revised manuscript and your response to the reviewers' comments. Your revised manuscript is also likely to be sent for further evaluation by the reviewers.

In particular, reviewer #1 wants to see the results from 2 cell lines in separate graphs and with a minimum of 10 data points/cell line, has several questions about figure 2, target cell lysis rate, NK cell death at 16 h time point, statistics, and presentation of 5 donors separately. Reviewer #1 also asks about the lipid composition of target cells, whether NK cells form conjugates with target cells in the presence of EDTA, and wants you to tone down the conclusions about tumor cell scape, saying that you should use a second resistant tumor cell line to validate your results. Reviewer #1 and #3 have concerns about the NK cell resistance to perforin in the absence of degranulation, and about the specificity of the 7KC treatment, suggesting to repeat the experiment with target cells. Reviewer #2 wants you to discuss more thoroughly previous similar work. Reviewer #2 and #3 have concerns due to 7KC not only impacting membrane order. Reviewer #3 has concerns with the small effect of 7KC on NK-cell autolysis, asks what happens with a flipped ratio of E:T, says that you should present data for PI uptake in CD107a-negative cells, and have further questions about 7KC-treated cells. Reviewer #3 also says that the data ruling out LAMP-1 is weak, and asks whether you can add a control to figure 5. Please address also the rest of the reviewers' comments.

We expect to receive your revised manuscript within 3 months. 

Please email us (plosbiology@plos.org) if you have any questions or concerns, or would like to request an extension. At this stage, your manuscript remains formally under active consideration at our journal; please notify us by email if you do not intend to submit a revision so that we may end consideration of the manuscript at PLOS Biology.

**IMPORTANT - SUBMITTING YOUR REVISION**

Your revisions should address the specific points made by each reviewer. Please submit the following files along with your revised manuscript:

1. A 'Response to Reviewers' file - this should detail your responses to the editorial requests, present a point-by-point response to all of the reviewers' comments, and indicate the changes made to the manuscript. 

*NOTE: In your point by point response to the reviewers, please provide the full context of each review. Do not selectively quote paragraphs or sentences to reply to. The entire set of reviewer comments should be present in full and each specific point should be responded to individually, point by point.

You should also cite any additional relevant literature that has been published since the original submission and mention any additional citations in your response. 

2. In addition to a clean copy of the manuscript, please also upload a 'track-changes' version of your manuscript that specifies the edits made. This should be uploaded as a "Related" file type. 

*Re-submission Checklist*

When you are ready to resubmit your revised manuscript, please refer to this re-submission checklist: https://plos.io/Biology_Checklist

To submit a revised version of your manuscript, please go to https://www.editorialmanager.com/pbiology/ and log in as an Author. Click the link labelled 'Submissions Needing Revision' where you will find your submission record. 

Please make sure to read the following important policies and guidelines while preparing your revision:

*Published Peer Review*

Please note while forming your response, if your article is accepted, you may have the opportunity to make the peer review history publicly available. The record will include editor decision letters (with reviews) and your responses to reviewer comments. If eligible, we will contact you to opt in or out. Please see here for more details:

https://blogs.plos.org/plos/2019/05/plos-journals-now-open-for-published-peer-review/

*PLOS Data Policy*

Please note that as a condition of publication PLOS' data policy (http://journals.plos.org/plosbiology/s/data-availability) requires that you make available all data used to draw the conclusions arrived at in your manuscript. If you have not already done so, you must include any data used in your manuscript either in appropriate repositories, within the body of the manuscript, or as supporting information (N.B. this includes any numerical values that were used to generate graphs, histograms etc.). For an example see here: http://www.plosbiology.org/article/info%3Adoi%2F10.1371%2Fjournal.pbio.1001908#s5

*Blot and Gel Data Policy*

We require the original, uncropped and minimally adjusted images supporting all blot and gel results reported in an article's figures or Supporting Information files. We will require these files before a manuscript can be accepted so please prepare them now, if you have not already uploaded them. Please carefully read our guidelines for how to prepare and upload this data: https://journals.plos.org/plosbiology/s/figures#loc-blot-and-gel-reporting-requirements

*Protocols deposition*

To enhance the reproducibility of your results, we recommend that if applicable you deposit your laboratory protocols in protocols.io, where a protocol can be assigned its own identifier (DOI) such that it can be cited independently in the future. For instructions see: https://journals.plos.org/plosbiology/s/submission-guidelines#loc-materials-and-methods

Thank you again for your submission to our journal. We hope that our editorial process has been constructive thus far, and we welcome your feedback at any time. Please don't hesitate to contact us if you have any questions or comments.

Sincerely,

Paula

---

Paula Jauregui, PhD,

Associate Editor,

pjaureguionieva@plos.org,

PLOS Biology

*****************************************************

REVIEWS:

Reviewer expertise:

Reviewer #1: Innate immunity and cancer.

Reviewer #2: Membrane structure/lipids.

Reviewer #3: Stephen Waggoner. NK cells.

Reviewer #1: This articles adresses the very important question of how NK cells (and possibly other cytotoxic lymphocytes) are protected against their own weapons, in particular perforin which, once released to the immunological synapse, promotes the formation of holes in the target cell membrane. 

Overall this is a very well written and interestig article. The introduction is well documented and focused on the question adressed in the subsequent experimental work. The methodology is appropriate and the experimental work is of great quality. The conclusion (and title) that « Degranulation-enhanced presynaptic membrane packing protects NK cells from perforin-mediated autolysis » are supported by a significant amount of results. I listed below several concerns that need to be adressed before publication.

Figure 1.

a) The data obtained with YTS and NK-92 are depicted in the same graphs (panels C and F and possibly panel I). It would be nice to see the results of the two cell lines in separate graphs.

b) With this regard, a minimum of 10 data points/cell line for proper statistical testing would be appreciated.

c) Is the unpaired t-test appropriate here?

Figure 2.

a) Is a target cell lysis rate of 30% the max. cytolytic capacity of NK cells even at high E:T ratios?

b) Fig. 2D, the values for the 16 hours coculture are suprisingly lower than 4 or 8 hours and this is not reported in the result section. Could this be explained by the higher rate of YTS death shown for the same time point in Fig. 2E?. By the way, how do you explain the increase of NK cell death in the 16 hours cytotoxicity assay (while other incubation times are all similar)? Alternatively, did the authors account for the increase in NK cell death and adjust the target cell lysis accordingly?

c) Healthy donor derived NKs: because there is significant heterogeneity between donor NK cells, I suggest to show each set of data (i.e. for each donor) in addition to (as a supplementary Figure?) or instead of the averages values. Ideally, five donors should be used. Finally, it would be important to check and report target cell killing with eNKs as well (similar to what has been shown with NK cell lines, Figure 2A and B). 

d) Statistical significance is sometimes missing. Not sure which test should be used. 

Figure 3. Do NK cells form conjugates with target cells in the presence of EDTA?

Figure 4.

a) Panel D, NK cells were exposed to free perforin. Without degranulation events, the NK cell membrane shows no increase in lipid order (which was proposed to drive resistance). Thus, how the authors explain NK cell resistance to perforin (in the absence of degranulation)? Is it possible that 7KC treatment destroys the overall cell membrane structure of resting NK cells rendering them more vulnerable to perforin? Testing of membrane leakiness after 7KC treatment with cytoplasmic dyes could provide more insight into the specificity of this treatment on perforin susceptibility

b) A repetition of this experiment with target cells would give indications whether NK cells have intrinsically a cell membrane of higher lipid order than other cells, providing them with a resistance "bonus".

Figure 6.

- What is the lipid composition of target cells?

- As previously requested for figure 2: could the authors provide the data obtained for each primary NK cell donor. Ideally, please include 2 additional donors to better taker into account inter donor heterogeneity. 

Figure 7

a) The legend is confusing. K562 and MB-231 cells are used in both panels E and F. Please check the figure legend. 

b) The susceptibility of MB-231 is only minimaly increased following 7KC treatment. Accordingly the role of densely-packed post synaptic lipid membrane in tumor cell escape should not be overestimated. In the discussion section, the authors are invited to tone down their conlcusions and clearly state that although lipid density might contribute to tumor cell resistance against NK cells, it can likely not account for most of the resistance. 

c) Do other (resistant) cancer cells respond to NK cell attack in the same way? Ideally a second resistant tumor cell line should be used to validate the results obtained with MD-231, in particular since the increase in susceptibility is not as high. 

d) What is the fate of target cells that generate densely packed postsynaptic membranes? Do they survive?

e) Is there a higher autolysis of NK cells when cultured with MDA-MB-231 cells due to the denser postsynaptic membrane?

Additionnal points:

a) I suggest the authors to more explicitely explain how LAMP-1 has previously been suggested to provide NK cells with perforin protection (line 80). Would it be relevant to refer to #34 here? 

b) I don't know what is PLOS Biology's policy oan siRNAs but most journals request to validate the results from gene KD with at least two different siRNAs. The "RNA interference" subsection in the Methods section only refers to the siRNA strategy for PRF1, while additional genes are knocked down using siRNAs. 

c) Line 635: the authors should refer to the original study: https://pubmed.ncbi.nlm.nih.gov/30104240/ (not only to a review article). 

d) Please check the statistical tests used throughout the article.

Reviewer #2: Overall, this manuscript describes what appear to be carefully done and extensive experiments probing (using membrane polarity sensitive dyes) and manipulating (using 7KC) the membrane order at the immune synapse, tying membrane order to the lytic activity of perforin in NK T-Cells.

I have 2 main comments/concerns:

The first being that the idea presented here is already present in the literature. This in itself is not a major problem, but I found it strange that it was not discussed more thoroughly. Namely, reference 36 of the manuscript is titled "Lipid order and charge

protect killer T cells from accidental death" and has a similar thesis to the present submission, although many of the experimental approaches differ.

My second main concern is the assertion that treatment with 7KC only impacts membrane order, which is certainly not the case. This lipid is added in a way that will also lead to the extraction of other lipids, especially cholesterol, and this could impact the observed processes in ways that do not depend exclusively on 'ordering'. This again is not necessarily a major problem as long as it is discussed appropriately. Ideally, experiments would also be done using other treatments that impact ordering in alternate ways, such as by adding bile acids or alcohols, feeding cells alternate fatty acids, or by altering lipid metabolism. 

A possible way to address both concerns would be to narrow the scope of the current manuscript to more centrally focus on the specific inhibitory role of 7KC in the title and abstract. 

Reviewer #3: This is a highly intriguing study from Li and Orange revealing formation of densely packed, highly ordered lipid membranes on NK cells at pre-synaptic sites of lytic granule fusion that prevent perforin binding and autolysis. They reveal that the unique lipid composition of lytic granules contributes to and/or reinforces this protection. Moreover, they show that an NK-cell resistant breast cancer cell line forms similar densely packed post-synaptic lipid membranes that contributes to evasion of NK-cell killing. The combination of fluorescent lipid packing sensors and microscopy is an asset that permits the additional strength of analysis across multiple NK-cell lines, primary NK cells, and tumor targets.

Concerns

1. While the effect of 7KC on high lipid order appears absolute in Figure 1, the effects on NK-cell autolysis in Figure 2 and elsewhere (notably Figure 7) are very small (this mechanism appears to protect 5% of NK cells). Intriguingly, these effects increase with effector cell density, potentially implicating fratricide over autolysis. What happens in an experimental setup with flipped ratio (1:10 E:T) that triggers greatest degranulation among large number of NK cells? While Figure 3G suggests degranulating cells appear to be most susceptible to death, data for PI uptake in CD107a-neagtive cells should be presented. In related queries, are the line colors mixed up in right graph in Figure 4A where NK92 survival would expected to be worst for 7KC-treated cells, which obfuscates the effect of CMA in this experiment. Finally, it is not clear why the measurement of 721.221 cell death in Figure 2D would decrease between 8 and 16 hours.

2. There is confusion about how Figure 4D fits with overall mechanism. If the synaptic formation of dense ordered lipid membranes that is enhanced by degranulation and is protective versus perforin, then what is the mechanism at play in resting NK cells treated with recombinant perforin? Is this different in activated NK cells?

3. On line 135, the results in Figure S2 are said to be similar to non-synaptic regions in Figure 1, but this is not readily apparent from the visible spectra representing general polarization values (bright yellow in unconjugated cells and dark orange to red in non-synaptic regions). Minimally this could be clarified with presentation of mean/SD values of GP for unconjugated cells. Is it feasible to capture images of lipid order in synapse and 7KC disruption (Figure 1) in primary NK cells (only tested in killing)? This would be a major plus but understandable if primary cells are less amenable to the imaging techniques employed.

4. The data ruling out LAMP-1 is weak. How do we know CD107a neutralizing antibodies worked as advertised? What is a positive control for this reagent? Likewise, in LAMP-1 knock-down cells, the LAMP-1 expression levels within granules (highly enriched) should be assessed as well as cell surface levels of CD107a after degranulation to ensure the knockdown was effective where it matters.

5. The approach to dissecting the role of dense ordered lipid membranes is restricted to use of 7-ketocholesterol. This is somewhat problematic given the toxicity of this drug in cells and the difficulty of determining that the only relevant effect of 7KC is the desired disruption of dense lipid formations rather than anti-mitotic or effects on mitochondrial membrane potential. The authors make several attempts to rule out direct toxicity of 7KC, which are good but could be strengthened. For example, not clear what cell density is used in Figure 3A (incubation of NK cells without targets), but would be important to use NK cells at same density as used in 10:1 E:T wells and to include controls of activated NK cells (without targets) to determine if a combination of 7KC and activation signaling is problematic for NK cells. Ideally, a second non-7KC method for disrupting these dense lipid formations if possible would be a huge improvement. A related question is whether the smaller size of 7KC-treated cells in Figure 1D/E is a consistent feature and how this might impact other measurements in this study?

6. Is there any potential control (i.e. loading control) for Figure 5? Disconcerting to see the shrinking numerator without any denominator. 

Minor points

1. There is a lot of interpretation and discussion contained within the results section that should ideally be restricted to the discussion.

2. Typo in Figure 1F legend (p=12, presumably meant to be 0.12). This value should be presented in place of ns on the figure image itself. All ns should be replaced with actual values.

3. If p<0.0001 is used a 'lower limit', then p<0.001 (Fig 1C), p<0.01 (Fig 4C), and p<0.05 (multiple spots) should be replaced with actual p values.

4. Colors in line graphs a bit hard to discern, not to mention for any readers who are colorblind.

5. Please put a label distinguishing the two pie charts in Figure 6C.

6. Statement on line 539 about GM3 should be cited to reference Table S1 since this information is not clear in Figure 6.

7. Control I and II labels in Figure 6E should be better defined (these are two non-synaptic regions apparently?)

8. Are bars switched in right bar graph in Figure S4?

9. Several images in Figure S5C and E are not discernable (appear teal)

10. Does green NK cell label on images in Figure 7 refer to YTS? B and C should be quantified like A and D.

# Author comment: Author response to Decision Letter 1

**DOI:** 10.1371/journal.pbio.3001328.r003

28 Apr 2021

Attachment Submitted filename: 20210421_Reply to reviewers.docx Click here for additional data file.

# Peer review: Decision Letter 2

**DOI:** 10.1371/journal.pbio.3001328.r004

**Authors:** Paula Jauregui, PhD

27 May 2021

Dear Dr. Orange,

Thank you for submitting your revised Research Article entitled "Degranulation-enhanced presynaptic membrane packing protects NK cells from perforin-mediated autolysis" for publication in PLOS Biology. I have now obtained advice from the original reviewers and have discussed their comments with the Academic Editor. 

Based on the reviews, we will probably accept this manuscript for publication, provided you satisfactorily address the following data and other policy-related requests.

ETHICS STATEMENT:

Since you are using cells from donors, please include information about the form of consent (written/oral) given for research involving human participants. All research involving human participants must have been approved by the authors' Institutional Review Board (IRB) or an equivalent committee, and all clinical investigation must have been conducted according to the principles expressed in the Declaration of Helsinki.

DATA POLICY:

You may be aware of the PLOS Data Policy, which requires that all data be made available without restriction: http://journals.plos.org/plosbiology/s/data-availability. For more information, please also see this editorial: http://dx.doi.org/10.1371/journal.pbio.1001797 

Note that we do not require all raw data. Rather, we ask that all individual quantitative observations that underlie the data summarized in the figures and results of your paper be made available in one of the following forms:

1) Supplementary files (e.g., excel). Please ensure that all data files are uploaded as 'Supporting Information' and are invariably referred to (in the manuscript, figure legends, and the Description field when uploading your files) using the following format verbatim: S1 Data, S2 Data, etc. Multiple panels of a single or even several figures can be included as multiple sheets in one excel file that is saved using exactly the following convention: S1_Data.xlsx (using an underscore).

2) Deposition in a publicly available repository. Please also provide the accession code or a reviewer link so that we may view your data before publication. 

Regardless of the method selected, please ensure that you provide the individual numerical values that underlie the summary data displayed in the following figure panels as they are essential for readers to assess your analysis and to reproduce it: Figures 1C1, 1F, 1I, 2A, 2B, 2C, 2D, 3A, 3B, 3C, 3D, 3E, 3F, 3G, 4A, 4B, 4C, 4D, 5B, 5D, 6B, 6C, 6E, 6F, 6G, 7F, 7G, 7H, Supplementary figures 1A, 1B, 3, 4A, 4B, 4C, 5B, 6, 7B, 7D, 7F, 8A, 8B, 8C, 8D, 9A, 9B.

NOTE: the numerical data provided should include all replicates AND the way in which the plotted mean and errors were derived (it should not present only the mean/average values).

**Please also ensure that figure legends in your manuscript include information on where the underlying data can be found, and ensure your supplemental data file/s has a legend.**

Please ensure that your Data Statement in the submission system accurately describes where your data can be found.

BLOT AND GEL REPORTING REQUIREMENTS:

We require the original, uncropped and minimally adjusted images supporting all blot and gel results reported in an article's figures or Supporting Information files. We will require these files before a manuscript can be accepted so please prepare and upload them now. Please carefully read our guidelines for how to prepare and upload this data: https://journals.plos.org/plosbiology/s/figures#loc-blot-and-gel-reporting-requirements. We will need this for the figures 5A, 5C, and supplementary figures 7A, 9A.

Please also add size bars to the microscopy pictures in figures 1A, 1B, 1D, 1E, 1G, 1H, 7A, 7B, 7C, 7D, supplementary figures 2A, 2B, 7C, 7E, and 9B.  

As you address these items, please take this last chance to review your reference list to ensure that it is complete and correct. If you have cited papers that have been retracted, please include the rationale for doing so in the manuscript text, or remove these references and replace them with relevant current references. Any changes to the reference list should be mentioned in the cover letter that accompanies your revised manuscript.

We expect to receive your revised manuscript within two weeks.

To submit your revision, please go to https://www.editorialmanager.com/pbiology/ and log in as an Author. Click the link labelled 'Submissions Needing Revision' to find your submission record. Your revised submission must include the following:

-  a cover letter that should detail your responses to any editorial requests, if applicable, and whether changes have been made to the reference list

-  a Response to Reviewers file that provides a detailed response to the reviewers' comments (if applicable)

-  a track-changes file indicating any changes that you have made to the manuscript. 

NOTE: If Supporting Information files are included with your article, note that these are not copyedited and will be published as they are submitted. Please ensure that these files are legible and of high quality (at least 300 dpi) in an easily accessible file format. For this reason, please be aware that any references listed in an SI file will not be indexed. For more information, see our Supporting Information guidelines:

https://journals.plos.org/plosbiology/s/supporting-information  

*Published Peer Review History*

Please note that you may have the opportunity to make the peer review history publicly available. The record will include editor decision letters (with reviews) and your responses to reviewer comments. If eligible, we will contact you to opt in or out. Please see here for more details:

https://blogs.plos.org/plos/2019/05/plos-journals-now-open-for-published-peer-review/

*Early Version*

Please note that an uncorrected proof of your manuscript will be published online ahead of the final version, unless you opted out when submitting your manuscript. If, for any reason, you do not want an earlier version of your manuscript published online, uncheck the box. Should you, your institution's press office or the journal office choose to press release your paper, you will automatically be opted out of early publication. We ask that you notify us as soon as possible if you or your institution is planning to press release the article.

*Protocols deposition*

To enhance the reproducibility of your results, we recommend that if applicable you deposit your laboratory protocols in protocols.io, where a protocol can be assigned its own identifier (DOI) such that it can be cited independently in the future. Additionally, PLOS ONE offers an option for publishing peer-reviewed Lab Protocol articles, which describe protocols hosted on protocols.io. Read more information on sharing protocols at https://plos.org/protocols?utm_medium=editorial-email&utm_source=authorletters&utm_campaign=protocols

Please do not hesitate to contact me should you have any questions.

Sincerely,

Paula

---

Paula Jauregui, PhD,

Associate Editor,

pjaureguionieva@plos.org,

PLOS Biology

 ------------------------------------------------------------------------

Reviewer remarks:

Reviewer #1: Clément Thomas

Reviewer #3: Stephen Waggoner

Reviewer #1: Most of my concerns have been properly addressed and I am in favor of publication of the revised version of the manuscript.

Reviewer #3: The authors were thorough and highly responsive in addressing the previous concerns of the referees. The revised manuscript is greatly improved.

# Author comment: Author response to Decision Letter 2

**DOI:** 10.1371/journal.pbio.3001328.r005

8 Jun 2021

Attachment Submitted filename: 20210421_Reply to reviewers.docx Click here for additional data file.

# Editor report: Decision Letter 3

**DOI:** 10.1371/journal.pbio.3001328.r006

**Authors:** Paula Jauregui, PhD

16 Jun 2021

Dear Dr. Orange,

On behalf of my colleagues and the Academic Editor, Hans-Uwe Simon, I am pleased to say that we can in principle offer to publish your Research Article "Degranulation enhances presynaptic membrane packing, which protects NK cells from perforin-mediated autolysis" in PLOS Biology, provided you address any remaining formatting and reporting issues. These will be detailed in an email that will follow this letter and that you will usually receive within 2-3 business days, during which time no action is required from you. Please note that we will not be able to formally accept your manuscript and schedule it for publication until you have made the required changes.

Please take a minute to log into Editorial Manager at http://www.editorialmanager.com/pbiology/, click the "Update My Information" link at the top of the page, and update your user information to ensure an efficient production process.

PRESS

We frequently collaborate with press offices. If your institution or institutions have a press office, please notify them about your upcoming paper at this point, to enable them to help maximise its impact. If the press office is planning to promote your findings, we would be grateful if they could coordinate with biologypress@plos.org. If you have not yet opted out of the early version process, we ask that you notify us immediately of any press plans so that we may do so on your behalf.

We also ask that you take this opportunity to read our Embargo Policy regarding the discussion, promotion and media coverage of work that is yet to be published by PLOS. As your manuscript is not yet published, it is bound by the conditions of our Embargo Policy. Please be aware that this policy is in place both to ensure that any press coverage of your article is fully substantiated and to provide a direct link between such coverage and the published work. For full details of our Embargo Policy, please visit http://www.plos.org/about/media-inquiries/embargo-policy/.

Thank you again for choosing PLOS Biology for publication and supporting Open Access publishing. We look forward to publishing your study. 

Sincerely, 

Paula 

---

Paula Jauregui, PhD 

Associate Editor 

PLOS Biology

pjaureguionieva@plos.org
